# Reference Values for Body Composition and Anthropometric Measurements in Athletes

**DOI:** 10.1371/journal.pone.0097846

**Published:** 2014-05-15

**Authors:** Diana A. Santos, John A. Dawson, Catarina N. Matias, Paulo M. Rocha, Cláudia S. Minderico, David B. Allison, Luís B. Sardinha, Analiza M. Silva

**Affiliations:** 1 Exercise and Health Laboratory, CIPER, Faculdade de Motricidade Humana, Universidade de Lisboa, Cruz-Quebrada, Portugal; 2 Office of Energetics, Nutrition Obesity Research Center, School of Public Health, University of Alabama at Birmingham, Birmingham, Alabama, United States of America; 3 Sport Medicine and Training Control Unit, Portuguese Institute of Sport and Youth, Cruz-Quebrada, Portugal; NIDDK/NIH, United States of America

## Abstract

**Background:**

Despite the importance of body composition in athletes, reference sex- and sport-specific body composition data are lacking. We aim to develop reference values for body composition and anthropometric measurements in athletes.

**Methods:**

Body weight and height were measured in 898 athletes (264 female, 634 male), anthropometric variables were assessed in 798 athletes (240 female and 558 male), and in 481 athletes (142 female and 339 male) with dual-energy X-ray absorptiometry (DXA). A total of 21 different sports were represented. Reference percentiles (5^th^, 25^th^, 50^th^, 75^th^, and 95^th^) were calculated for each measured value, stratified by sex and sport. Because sample sizes within a sport were often very low for some outcomes, the percentiles were estimated using a parametric, empirical Bayesian framework that allowed sharing information across sports.

**Results:**

We derived sex- and sport-specific reference percentiles for the following DXA outcomes: total (whole body scan) and regional (subtotal, trunk, and appendicular) bone mineral content, bone mineral density, absolute and percentage fat mass, fat-free mass, and lean soft tissue. Additionally, we derived reference percentiles for height-normalized indexes by dividing fat mass, fat-free mass, and appendicular lean soft tissue by height squared. We also derived sex- and sport-specific reference percentiles for the following anthropometry outcomes: weight, height, body mass index, sum of skinfold thicknesses (7 skinfolds, appendicular skinfolds, trunk skinfolds, arm skinfolds, and leg skinfolds), circumferences (hip, arm, midthigh, calf, and abdominal circumferences), and muscle circumferences (arm, thigh, and calf muscle circumferences).

**Conclusions:**

These reference percentiles will be a helpful tool for sports professionals, in both clinical and field settings, for body composition assessment in athletes.

## Introduction

Assessment of body composition in athletes may help to optimize competitive performance and monitor the success of training regimens and thus is of considerable interest to sports professionals.[1], [2], [3] It has been stated that improved body composition in athletes is associated with enhancements in cardiorespiratory fitness [4], [5] and strength.[6], [7], [8] Body composition may also be related to health complications, because medical problems may arise in athletes with very low body mass, extreme mass changes due to dehydration, or eating disorders.[9], [10]

Body composition can be organized according to a comprehensive model that consists of five levels of increasing complexity: I, atomic; II, molecular; III, cellular; IV, tissue-system; and V, whole-body [11]. Most studies of athletic populations are focused mainly on estimation of molecular compartments and the description of whole-body parameters.

The whole-body level of body composition characterizes body size and configuration, which is often described by anthropometric measures such as body weight, skinfold thicknesses, circumferences, and body mass index (BMI) among others. [12]

On the other hand, the molecular level consists of six main components: water, lipid, protein, carbohydrates, bone minerals, and soft tissue minerals. Several models ranging from two to six components can be created at this level of analysis.[12] Owing to its good precision, availability, and low radiation dose, dual-energy X-ray absorptiometry (DXA) is a convenient and useful tool for body composition assessment.[13] For athletes, DXA measurement presents an excellent alternative to reference methods because of its speed (fan-beam densitometers), but also because the measurement is minimally influenced by fluctuations in the water component.[1], [14], [15] Furthermore, DXA allows both regional and total body composition estimates, characterizing fat mass (FM) and dividing fat-free mass (FFM) into two components, lean soft tissue (LST) and bone mineral content (BMC).[12], [13], [16]

Reference values for DXA results have been developed for North Americans aged 8 to 85 years old by use of the NHANES dataset.[17] To our knowledge, however, no study has presented reference values for body composition in athletic populations within sports. Thus, the aim of the current study was to provide reference data for anthropometry and DXA outputs for male and female athletes from different sports during the in-season training period.

## Materials and Methods

### Ethics Statement

All subjects and their parents or guardians were informed about the possible risks of the investigation before giving written informed consent to participate. All procedures were approved by the ethics committee of the Faculty of Human Kinetics, Technical University of Lisbon, and were conducted in accordance with the declaration of Helsinki for human studies of the World Medical Association. [18]

### Participants

With the use of a cross-sectional design, body weight and height were measured in 898 athletes (264 females, 634 males), anthropometric variables (skinfolds and circumferences) were assessed in 798 athletes (240 females and 558 males), and 481 athletes (142 females and 339 males) were evaluated with DXA during the in-season period. From these athletes, a total of 381 (119 females and 262 males) were assessed for both measurements, whereas anthropometric or DXA measures were only obtained for the remaining participants. The sample included athletes involved in a total of 21 sports. In Table 1, the sample sizes and descriptive statistics for age are provided for the three general classifications of outcomes listed above, stratified by sex and sport.

**Table 1:** Number of participants and respective age by sport and sex.

		Common Athletes^c)^	Weight and Height			Skinfolds and Circumferences			DXA		
Sport	sex	n	n	Age (range)	Age (mean ± SD)	n	Age (range)	Age (mean ± SD)	N	Age (range)	Age (mean ± SD)
Archery and Shooting	Female	0	4#	25–45	33.5	4#	25–45	33.5	0	NA	NA
	Male	0	9	16–50	30.9±13.1	9	16–50	30.9±13.1	0	NA	NA
Athleticsa)	Female	9	32	16–30	21.8±4.1	25	17–30	22.0±3.9	16	16–30	21.3±4.1
	Male	4	30	17–31	21.6±3.5	23	17–31	21.9±3.6	11	17–26	20.1±3.0
Basketball	Female	31	43	16–34	17.3±2.7	39	16–34	17.4±2.8	34	16–19	16.9±0.8
	Male	44	47	16–18	16.8±0.7	46	16–18	16.8±0.7	45	16–18	16.8±0.7
Fencing	Female	0	4#	18–25	20.5	4#	18–25	20.5	0	NA	NA
	Male	0	12	17–24	20.6±2.5	12	17–24	20.6±2.5	0	NA	NA
Gymnastics	Female	12	18	16–23	18.3±2.4	18	16–23	18.3±2.4	12	16–19	17.1±1.1
	Male	2	20	16–31	21.2±4.3	20	16–31	21.2±4.3	2#	16–17	16.5
Handball	Female	4	4#	19–31	25.3	4#	19–31	25.3	4#	19–31	25.3
	Male	20	37	17–38	21.4±4.8	20	17–21	19.1±1.1	37	17–38	21.5±4.8
Hockey Rink	Female	0	0	NA	NA	0	NA	NA	0	NA	NA
	Male	1	49	16–36	20.5±5.4	48	16–36	20.4±5.4	2	17–25	21.0±5.7
Korfball	Female	0	9	18–30	21.2±3.6	9	18–30	21.2±3.6	0	NA	NA
	Male	0	11	16–31	22.7±5.3	11	16–31	22.7±5.3	0	NA	NA
Modern Pentathlon	Female	1	9	16–23	18.6±2.6	8	16–23	18.8±2.7	2#	17–17	17.0
	Male	5	14	16–28	19.9±4.4	14	16–28	19.9±4.4	5#	16–24	18.8
Motorsport	Female	0	0	NA	NA	0	NA	NA	0	NA	NA
	Male	0	7#	17–33	26.0	7#	17–33	26.0	0	NA	NA
Other combat sports^b)^	Female	0	15	16–24	18.5±2.4	11	16–23	18.5±2.2	4#	17–24	18.8
	Male	8	34	16–29	21.1±4.1	29	16–29	21.6±4.2	13	17–29	22.5±4.2
Rowing	Female	1	8	16–31	23.4±6.7	8	16–31	23.4±6.7	1#	16–16	16.0
	Male	6	27	16–32	21.1±4.5	27	16–32	21.1±4.5	6#	16–17	16.8
Rugby	Female	0	0	NA	NA	0	NA	NA	0	NA	NA
	Male	39	62	16–33	20.4±4.0	62	16–33	20.4±4.0	39	16–28	18.2±2.1
Sailing	Female	0	7#	16–27	20.6	7#	16–27	20.6	0	NA	NA
	Male	3	38	16–40	25.0±7.6	37	16–40	25.1±7.7	4#	19–35	26.0
Soccer	Female	0	22	16–37	22.5±5.7	22	16–37	22.5±5.7	0	NA	NA
	Male	3	42	17–36	19.7±4.1	17	18–36	22.3±5.5	28	17–19	18.0±0.8
Surf	Female	1	1#	33–33	33.0	1#	33–33	33.0	1#	33–33	33.0
	Male	0	1#	31–31	31.0	1#	31–31	31.0	0	NA	NA
Swimming	Female	22	26	16–20	17.2±1.3	26	16–20	17.2±1.3	22	16 – 20	17.0±1.2
	Male	34	44	16–30	19.6±3.4	42	16–30	19.4±3.3	36	16 -30	19.1±3.4
Tennis	Female	4	11	16–24	18.0±2.7	10	16–24	18.1±2.8	5#	16–24	19.0
	Male	7	23	16–34	20.4±5.2	19	16–34	19.8±5.4	11	1 6–34	23.6±5.3
Triathlon	Female	6	11	16–27	21.0±3.5	8	16–27	21.7±4.0	10	16–26	20.4±3.1
	Male	30	41	16–35	23.0±5.4	33	16–35	23.1±5.5	38	16–35	22.9±5.4
Volleyball	Female	16	16	18–36	25.9±5.9	16	18–36	25.9±5.9	16	18–36	25.9±5.9
	Male	17	17	23–33	27.8±2.5	17	23–33	27.8±2.5	17	23–33	27.8±2.5
Wrestling and Judo^c)^	Female	12	24	16–33	20.4±5.3	21	16–33	19.7±5.2	15	16–33	22.3±5.8
	Male	39	69	16–45	21.0±5.0	64	16–37	20.6±4.1	45	16–45	21.8±5.1

a) Athletics: includes sprinters, hurdlers, and jumpers (long and triple jump); ^b)^ other combat sports: includes karate, taekwondo, and kickboxing.

Abbreviations: DXA, dual-energy X-ray absorptiometry; NA, data not available; ^c)^Participants with crossover measures of skinfolds and circumferences and DXA.

# standard deviation was not presented for n<8.

Athletes involved in this study were subject to the following inclusion criteria: 1) in at least Tanner stage V [determined by self-evaluation [19]], 2) 10 or more hours of training per week, 3) negative test outcomes for performance-enhancing drugs, and 4) not taking any medications. The results of a medical screening indicated that all subjects were in good health.

### Measurements

#### Anthropometry

Subjects came to the laboratory refraining from exercise and alcohol or stimulant beverages and fasting for at least 3 hours. Body weight was measured with a scale without shoes and wearing minimal clothes, to the nearest 0.01 kg and height was measured to the nearest 0.1 cm with a stadiometer (Seca, Hamburg, Germany) according to standardized procedures described elsewhere[20]. Skinfold thickness measurements were made according to standardized procedures [20] by use of a Slim Guide calliper (Creative Health Products, Ann Arbor, MI, USA). Skinfold measurement included triceps, subscapular, biceps, suprailiac, abdominal, thigh, and medial calf. The sum of the 7 skinfolds (∑7SKF); the sum of the appendicular (triceps, biceps, thigh, and medial calf) skinfolds (∑_app_SKF); the sum of the trunk (subscapular, suprailiac, and abdominal) skinfolds (∑_trunk_SKF); the sum of the arm (triceps and biceps) skinfolds (∑_arm_SKF); and the sum of the leg (thigh and medial calf) skinfolds (∑_leg_SKF) were used. Hip, arm, midthigh, calf, [20] and abdominal [21] circumferences were measured according to standard procedures by using an anthropometric tape (Lufkin W606PM; Apex Tool Group, Sparks, MD, USA). Arm, thigh, and calf circumferences were converted into muscle circumferences by correcting the circumferences for the respective skinfold by use of the following formula: Circumference – (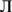 SKF).[22] Arm, thigh, and calf muscle circumferences were corrected for triceps, thigh, and calf skinfolds, respectively.

Two-way, mixed–model, single measures intra-class correlation coefficients [ICC(3,1)] were used to assess reliability between two certified anthropometrists for skinfolds and circumferences for five active male subjects.[23] The ICC ranged from 0.776 to 1.00 for skinfolds and from 0.875 to 0.992 for circumferences. An ICC of 1.00 was observed for weight and height.

#### Dual-energy X-ray absorptiometry

The subjects came to the laboratory after an overnight fast (12 h fast), refraining from vigorous exercise at least 15 h, no caffeine and alcohol during the preceding 24 h, and consuming a normal evening meal the night before. Athletes underwent a whole-body DXA scan according to the procedures recommended by the manufacturer on a Hologic Explorer-W fan-beam densitometer (Hologic, Waltham, MA, USA). The equipment measures the attenuation of X-rays pulsed between 70 and 140 kV synchronously with the line frequency for each pixel of the scanned image. According to the protocol described by the manufacturer, a step phantom with six fields of acrylic and aluminium of varying thicknesses and known absorptive properties was scanned to serve as an external standard for the analysis of different tissue components. For athletes who were taller than the scan area, we used a validated procedure that consisted of the sum of a head and a trunk plus limbs scans. [24] The same technician positioned the participants, performed the scan, and executed the analysis (QDR for Windows software version 12.4; Hologic, Waltham, MA, USA) according to the operator's manual by using the standard analysis protocol.

The DXA measurements included whole-body measurements of BMC (g), bone mineral density (BMD, g/cm^2^), absolute FM (kg), percentage FM (%FM), FFM (kg), and LST (kg). With the exception of BMD, the remaining variables were also presented for predefined subregions, including trunk, appendicular (arms+legs), and subtotal (whole body minus the head) regions. Additionally, from these measures the following variables were calculated: fat mass index (FMI) = FM/height^2^ (kg/m^2^), fat-free mass index (FFMI) = FFM/height^2^ (kg/m^2^), and appendicular lean soft tissue index (ALSTI) = ALST/height^2^ (kg/m^2^).

Two-way, mixed-model single measures ICCs [ICC(3,1)] [23] were used to assess reliability between measurements for DXA outputs on the basis of 10 young active adults (5 males and 5 females). These coefficients are shown in Table 2.

**Table 2:** Coefficients of variation in our laboratory for dual-energy X-ray absorptiometry measurements.

	Whole-body	Subtotal	Appendicular	Trunk
**BMC**	1.3%	0.9%	0.9%	2.5%
**BMD**	1.4%			
**Absolute FM**	1.7%	1.8%	2.8%	4.3%
**Percent FM**	1.6%	1.7%	2.1%	3.6%
**FFM**	0.8%	0.6%	1.6%	1.2%
**LST**	0.8%	0.6%	1.2%	1.3%

Abbreviations: BMC, bone mineral content; BMD, bone mineral density; FM, fat mass; FFM, fat-free mass; LST, lean soft tissue.

### Statistical Analysis

Analyses were performed to complete three tasks: 1) estimate the reference percentiles for each outcome, stratified by sex and sport; 2) test whether the mean for each outcome differed by sex, stratified by sport; and 3) within each outcome, identify sports for which the mean value differed from the others (if any), stratified by sex.

#### Estimating reference percentiles

Because the sample sizes were very low in many of the outcome/sex/sport combinations, the reference percentiles were estimated through a parametric, empirical Bayesian framework that allowed the sharing of information across sports to augment our inference whenever we had at least two athletes' values. Within a given sex and sport, the athletes' outcome values were assumed to follow a normal (Gaussian) distribution that could be characterized through its mean and precision (inverse variance). If the mean and precision were known, all quantiles followed immediately from the normal assumption. We note that in some cases, raw outcome values were decidedly non-normal, in that they were not unimodal and were skewed strongly to the right and thus were not even roughly symmetric about the mean. Therefore, for BMD, FM, %FM, FMI, FFMI, and ALSTI, raw values were log transformed before running the approach for all subjects and then transformed back afterward, allowing us to gain unimodality and rough symmetry about the mean while maintaining the original units for all results.

The sport-specific means and variances were modelled as arising from a normal-gamma, which serves as the prior and forms a conjugate family with our observational model. The hyperparameters of the prior were informed empirically through maximum-likelihood by using all athletes' data for this outcome, restricted by sex. Once this was done, joint posterior distributions for the mean and precision were generated for every sport, giving rise to point estimates and 95% joint confidence regions for the mean and precision, which in turn were used to calculate simultaneous 95% confidence intervals for the reference percentiles of interest.

Percentiles (5^th^, 25^th^, 50^th^, 75^th^, and 95^th^ were presented for all sex/sport subsamples with n≥8)

All computations were performed in R, version 2.14.2.[25]

#### Comparisons between the sexes by sports

Descriptive statistics (means and 95% confident intervals of the mean) for the main outcome variables were calculated with IBM SPSS Statistics version 21.0, 2012 (IBM, Chicago, IL, USA). Normality was tested by using the Shapiro-Wilk test. Sex comparisons were performed with unpaired t-tests or the non-parametric equivalent, the Mann-Whitney U test. Comparisons across sports were made by sex by using the Kruskal-Wallis test with pairwise comparisons performed by using the Dunn test. For both sex and sport comparisons, the p-values presented are nominal, (i.e. unadjusted for multiple comparisons across outcomes and either sex or sports, as appropriate).

## Results

The variables derived from the DXA and anthropometry measures are listed in Table 3. The reference values for each of the DXA and anthropometry outputs described in Table 3 are provided as Supporting Information (Files S1 and S2) by sport and sex. The percentiles presented include the 5^th^, 25^th^, 50^th^, 75^th^, and 95^th^ percentiles.

**Table 3:** List of quantiles derived from dual-energy X-ray absorptiometry (DXA) and anthropometry measures.

Supplemental Digital Content (File S1)		Supplemental Digital Content (File S2)	
Anthropometry variable	Table	DXA variable	Table
Weight (kg)	TABLE S1	WB BMC (g)	TABLE S1
Height (cm)	TABLE S2	WB BMD (g/cm^2^)	TABLE S2
BMI (kg/m^2^)	TABLE S3	WB FM (kg)	TABLE S3
∑7SKF (mm)	TABLE S4	WB FMI (kg/m^2^)	TABLE S4
∑Appendicular SKF (mm)	TABLE S5	WB FM (%)	TABLE S5
∑Arm SKF (mm)	TABLE S6	WB FFM (kg)	TABLE S6
∑Leg SKF (mm)	TABLE S7	WB FFMI (kg/m^2^)	TABLE S7
∑Trunk SKF (mm)	TABLE S8	WB LST (kg)	TABLE S8
Arm circumference (cm)	TABLE S9	Subtot BMC (kg)	TABLE S9
Arm muscle circumference (cm)	TABLE S10	Subtot FM (kg)	TABLE S10
Thigh circumference (cm)	TABLE S11	Subtot FM (%)	TABLE S11
Thigh muscle circumference (cm)	TABLE S12	Subtot FFM (kg)	TABLE S12
Calf circumference (cm)	TABLE S13	Subtot LST (kg)	TABLE S13
Calf muscle circumference (cm)	TABLE S14	Appendicular BMC (kg)	TABLE S14
Abdominal circumference (cm)	TABLE S15	Appendicular FM (kg)	TABLE S15
Hip circumference (cm)	TABLE S16	Appendicular FM (%)	TABLE S16
		Appendicular FFM (kg)	TABLE S17
		Appendicular LST (kg)	TABLE S18
		Appendicular LSTI (kg/m^2^)	TABLE S19
		Trunk BMC (kg)	TABLE S20
		Trunk FM (kg)	TABLE S21
		Trunk FM (%)	TABLE S22
		Trunk FFM (kg)	TABLE S23
		Trunk LST (kg)	TABLE S24

Abbreviations: BMI, body mass index; SKF, skinfolds; WB, whole body; BMC, bone mineral content; BMD, bone mineral density; FM, fat mass; FMI, fat mass index; FFM; fat-free mass; FFMI; fat-free mass index; LST, lean soft tissue; Subtot; subtotal; LSTI; lean soft tissue index.

Descriptive statistics for the main anthropometry and DXA variables are presented in Tables 4 and 5, respectively. Significant differences were observed between the sexes for almost all variables and the differences varied by sport. In addition, the means of some variables differed across all sports, as described in Tables 4 and 5.

**Table 4:** Body composition for the main anthropometry outputs.

Sport	Sex	n	Weight (kg) ^b, c^	Height (cm)^b, c^	BMI (kg/m^2^) ^b, c^	n	∑7SKF (mm) ^b, c^	Arm MC* (cm) ^b, c^	Thigh MC* (cm) ^b, c^	Calf MC* (cm) ^b, c^
1. Archery and Shooting	female	4^#^	58.3^a^	162.7^a^	22.0	4^#^	143.9^a^	20.5^a^	40.6^a^	32.5 (31.2; 33.9)^a^
	male	9	73.1 (65.4; 80.8)	177.6 (174.4; 180.8)	23.2 (20.5; 26.0)	9	91.6 (69.2; 114.1)	26.0 (24.0; 27.9)	44.3 (42.6; 45.9)	33.0 (31.7; 34.3)
2. Athletics	female	32	59.1 (56.8; 61.4)^a^	166.3 (164.1; 168.6)^a^	21.3 (20.8; 21.8)^a^	25	71.4 (61.1; 81.6)^a^	22.6 (21.9; 23.3)^a^	46.4 (45.0; 47.9)^a^	30.7 (29.5; 31.9)^a^
	male	30	73.9 (71.5; 76.3)	182.1 (180.1; 184.2)	22.3 (21.7; 22.8)	23	46.6 (41.6; 51.6)	27.1 (26.3; 27.9)	51.2 (49.6; 52.8)	35.9 (35.0; 36.9)
3. Basketball	female	43	68.9 (66.0; 71.7)^a^	176.7 (174.2; 179.3)^a^	22.0 (21.4; 22.6)	39	126.8 (114.8; 138.7)^a^	21.8 (21.2; 22.3)^a^	45.9 (44.9; 46.8)^a^	31.8 (30.9; 32.7)^a^
	male	47	81.9 (78.8; 85.0)	190.6 (187.7; 193.5)	22.5 (21.9; 23.1)	46	73.3 (66.3; 80.3)	27.1 (26.4; 27.7)	50.8 (49.8; 51.8)	34.9 (33.5; 36.3)
4. Fencing	female	4^#^	61.7^a^	166.3^a^	22.3	4^#^	121.5^a^	20.7^a^	45.3^a^	30.4^a^
	male	12	72.6 (68.0; 77.1)	180.0 (176.4; 183.6)	22.4 (21.3; 23.5)	12	63.8 (49.9; 77.7)	25.0 (24.2; 25.9)	52.3 (50.4; 54.2)	33.4 (32.3; 34.6)
5. Gymnastics	female	18	53.2 (50.4; 56.0)^a^	160.7 (157.4; 164.1)^a^	20.6 (19.8; 21.4)^a^	18	91.4 (79.9; 103.0)^a^	21.1 (20.2; 22.0)^a^	45.4 (43.4; 47.4)	29.7 (28.7; 30.7)^a^
	male	20	65.9 (62.7; 69.1)	169.9 (167.1; 172.7)	22.9 (21.6; 24.1)	20	58.5 (49.1; 68.0)	26.8 (25.9; 27.8)	45.2 (43.6; 46.7)	34.4 (33.6; 35.1)
6. Handball	female	4^#^	67.9^a^	167.3^a^	24.2	4^#^	128.1^a^	24.2^a^	49.2	30.7^a^
	male	37	83.7 (80.1; 87.3)	183.4 (181.4; 185.5)	24.8 (24.0; 25.7)	20	86.7 (72.0; 101.3)	27.7 (26.7; 28.8)	48.9 (47.3; 50.6)	35.4 (34.5; 36.4)
7. Hockey Rink	female	0	NA	NA	NA	0	NA	NA	NA	NA
	male	49	74.9 (72.6; 77.2)	174.8 (173.4; 176.3)	24.5 (23.8; 25.2)	48	80.7 (72.6; 88.9)	26.4 (25.8; 27.1)	49.2 (48.3; 50.1)	34.9 (34.1; 35.8)
8. Korfball	female	9	58.5 (54.0; 63.1)^a^	162.4 (155.7; 169.1)^a^	22.2 (20.7; 23.7)	9	114.5 (90.1; 138.9)^a^	20.9 (20.4; 21.5)^a^	40.0 (38.0; 42.0)^a^	36.3 (35.5; 37.1)^a^
	male	11	72.3 (67.1; 77.5)	181.2 (177.1; 185.4)	22.0 (20.6; 23.4)	11	61.7 (49.6; 73.8)	25.3 (23.6; 27.0)	45.4 (43.9; 46.9)	35.7 (34.5; 36.8)
9. Modern Pentathlon	female	9	60.8 (53.6; 68.1)^a^	170.6 (165.6; 175.7)^a^	20.8 (18.8; 22.9)	8	87.0 (64.2; 109.9)^a^	21.8 (20.1; 23.5)^a^	44.7 (43.4; 46.0)^a^	31.3 (30.3; 32.3)^a^
	male	14	69.0 (64.2; 73.7)	176.9 (173.8; 179.9)	22.1 (20.6; 23.5)	14	56.3 (45.9; 66.7)	26.7 (25.4; 28.0)	50.5 (46.1; 54.9)	34.6 (33.5; 35.8)
10. Motorsport	female	0	NA	NA	NA	0	NA	NA	NA	NA
	male	7^#^	73.9	175.5	23.9	7^#^	81.9	26.3	49.3	34.0
11. Other combat sports	female	15	59.0 (55.5; 62.5)^a^	162.8 (159.3; 166.4)^a^	22.3 (21.0; 23.5)	11	107.3 (80.8; 133.8)^a^	22.6 (21.4; 23.7)^a^	44.7 (42.8; 46.6)^a^	28.5 (26.4; 30.6)^a^
	male	34	70.3 (67.3; 73.2)	175.9 (173.8; 178.0)	22.7 (21.9; 23.5)	29	62.6 (54.9; 70.4)	26.9 (26.2; 27.6)	49.9 (48.8; 51.0)	34.4 (32.8; 36.0)
12. Rowing	female	8	66.1 (60.9; 71.2)^a^	169.9 (165.2; 174.6)^a^	22.9 (21.8; 24.0)	8	104.5 (87.1; 122.0)^a^	22.1 (21.0; 23.2)^a^	47.2 (44.9; 49.6)^a^	32.4 (26.4; 38.4)^a^
	male	27	78.5 (75.0; 82.1)	183.0 (180.8; 185.2)	23.4 (22.6; 24.2)	27	60.5 (52.9; 68.2)	27.8 (27.2; 28.4)	50.1 (49.0; 51.1)	10.2 (36.8; 36.0)
13. Rugby	female	0	NA	NA	NA	0	NA	NA	NA	NA
	male	62	92.2 (88.1; 96.3)	182.8 (180.9; 184.7)	27.6 (26.4; 28.7)	62	110.5 (96.0; 125.1)	31.1 (30.2; 32.0)	52.5 (51.3; 53.7)	36.8 (36.0; 37.5)
14. Sailing	female	7^#^	62.5^a^	170.2^a^	21.5^a^	7^#^	90.9	23.2^a^	43.9^a^	32.5
	male	38	76.1 (72.1; 80.2)	177.9 (175.3; 180.4)	23.9 (23.1; 24.8)	37	89.7 (77.9; 101.5)	26.7 (25.6; 27.8)	48.1 (46.0; 50.1)	33.9 (32.8; 34.9)
15. Soccer	female	22	59.7 (56.8; 62.6)^a^	164.1 (161.5; 166.6)^a^	22.2 (21.3; 23.0)^a^	22	105.5 (93.6; 117.4)^a^	21.6 (20.7; 22.5)^a^	44.7 (43.2; 46.2)^a^	32.5 (29.8; 35.1)^a^
	male	42	73.8 (71.4; 76.2)	176.6 (174.8; 178.4)	23.6 (23.1; 24.2)	17	58.1 (52.0; 64.2)	27.7 (26.5; 28.9)	50.9 (49.4; 52.4)	35.2 (33.7; 36.6)
16. Surf	female	1				1				
	male	1				1				
17. Swimming	female	26	59.3 (57.1; 61.5)^a^	167.7 (165.3; 170.1)^a^	21.1 (20.5; 21.7)^a^	26	93.1 (82.6; 103.6)^a^	23.9 (23.1; 24.7)^a^	44.2 (43.1; 45.2)^a^	33.0 (32.2; 33.8)^a^
	male	44	72.0 (69.4; 74.7)	179.9 (177.8; 182.0)	22.2 (21.7; 22.8)	42	56.6 (50.4; 62.8)	30.9 (26.6; 35.1)	48.2 (47.3; 49.1)	34.4 (33.8; 34.9)
18. Tennis	female	11	64.2 (59.9; 68.5)^a^	168.5 (165.0; 172.0)^a^	22.6 (21.5; 23.7)	10	141.0 (123.4; 158.7)^a^	21.9 (19.4; 24.3)^a^	41.0 (38.0; 44.0)^a^	31.2 (30.4; 31.9)^a^
	male	23	71.3 (67.1; 75.5)	177.4 (174.8; 180.1)	22.6 (21.5; 23.8)	19	67.7 (59.3; 76.1)	25.6 (24.5; 26.7)	46.7 (45.0; 48.4)	34.1 (33.2; 35.0)
19. Triathlon	female	11	57.9 (53.5; 62.3)^a^	168.4 (163.8; 173.0)^a^	20.4 (19.1; 21.8)	8	86.3 (46.1; 126.5)	23.2 (20.8; 25.6)^a^	45.3 (42.2; 48.5)	31.6 (30.6; 32.6)^a^
	male	41	65.9 (64.6; 67.2)	175.8 (174.0; 177.6)	21.3 (20.9; 21.7)	33	49.6 (45.6; 53.6)	26.2 (25.7; 26.7)	47.1 (46.2; 48.0)	34.2 (33.6; 34.7)
20. Volleyball	female	16	67.7 (61.6; 73.8)^a^	174.5 (169.3; 179.8)^a^	22.1 (21.0; 23.2)^a^	16	118.0 (99.1; 136.8)^a^	23.0 (21.8; 24.3)^a^	46.5 (44.5; 48.6)^a^	29.8 (28.5; 31.2)^a^
	male	17	90.1 (86.3; 94.0)	195.0 (191.5; 198.4)	23.7 (22.9; 24.5)	17	70.0 (59.8; 80.1)	31.1 (29.6; 32.6)	52.5 (51.1; 53.9)	36.9 (35.8; 37.9)
21. Wrestling and Judo	female	24	59.455.6; 63.2)^a^	162.1 (159.2; 165.0)^a^	22.5 (21.6; 23.5)^a^	21	123.0 (104.0; 142.0)^a^	22.3 (21.1; 23.6)^a^	44.3 (42.6; 46.1)^a^	29.1 (27.6; 30.6)^a^
	male	69	71.5 (69.2; 73.7)	172.7 (171.1; 174.2)	23.9 (23.3; 24.5)	64	59.4 (53.6; 65.2)	29.0 (28.2; 29.8)	49.4 (48.7; 50.1)	34.5 (33.3; 35.7)
All samples	female	264	61.5 (60.5; 62.6)^a^	167.8 (166.8; 168.8)^a^	21.8 (21.5; 22.0)^a^	240	106.8 (102.1; 111.4)^a^	22.3 (22.0; 22.5)^a^	44.9 (44.4; 45.4)^a^	31.2 (30.8; 31.5)^a^
	male	634	76.1 (75.1; 77.0)	179.3 (178.6; 180.0)	23.6 (23.4; 23.8)	558	71.4 (68.6; 74.2)	27.9 (27.5; 28.3)	49.5; 49.1; 49.8)	35.0 (34.8; 35.2)
Sport Comparisons	female		^b)^5≠18, 12, 20, 3; 2, 21≠3	^b)^5, 21, 11≠20, 3; 8, 15≠3	NS		^b)^2≠15, 20, 21, 3, 18,1; 17≠3, 18; 5≠18	^b)^8, 5, 15≠17	^b)^8≠3, 2,20, 12, 6; 18≠2, 6	^b)^21≠20, 2, 3; 5≠2
	male		^c)^5, 19,≠7, 12, 3, 6, 20, 13; 19≠15, 2, 14; 9, 18, 11,≠3, 6, 20, 13	^c)^5, 21≠14, 17, 2, 13, 12, 6, 3, 20; 5≠8; 7≠2, 13, 12, 6, 3, 20; 10≠3, 20; 19≠2	^c)^19, 2≠3, 1, 14, 7, 6, 13; 2≠20; 9≠13; 17, 21≠14, 7, 6, 13		^c)^2, 19≠3, 7, 6, 14, 1, 13; 2≠20; 9≠13; 17, 21≠7, 6, 14, 13	^c)^4≠17; 4, 8, 18, 19,≠21, 20, 13; 1≠20, 13	^c)^1, 5≠21, 11; 5≠7; 1, 5, 8, 19,≠12, 3, 15, 2, 4, 20, 13; 18≠3	^c)^1, 5≠3, 13, 20; 18≠13

Abbreviations: BMI, body mass index; ∑7SKF, sum of seven skinfolds (triceps, subscapular, biceps, suprailiac, abdominal, thigh, and medial calf); MC, muscle circumference.

*MC: Arm muscle circumference  =  arm circumference - (π triceps skinfold); thigh muscle circumference  =  thigh circumference - (π thigh skinfold); Calf muscle circumference  =  medial calf circumference - (π medial skinfold). ^#^95% confident intervals were not presented for n<8. NA: data not presented for n<2. ^a)^ Significantly different from males (p<0.05); ^b)^ Significant differences between sports for females (p<0.05); ^c)^ Significant differences between sports for males (p<0.05).

**Table 5:** Body composition for the Dual-energy X-ray absorptiometry outputs.

Sport	g	n	WB BMC (g) ^b, c^	WB BMD (g/cm^2^) ^b, c^	WB FM (kg) ^b, c^	WB FM (%) ^b, c^	FMI (kg/m^2^) ^b, c^	WB FFM (kg) ^b, c^	FFMI (kg/m^2^) ^b, c^	WB LST (kg) ^b, c^	ALST (kg) ^b, c^	ALSTI (kg/m^2^) ^b, c^
1. Archery and Shooting	female	0	NA	NA	NA	NA	NA	NA	NA	NA	NA	NA
	male	0	NA	NA	NA	NA	NA	NA	NA	NA	NA	NA
2. Athletics	female	16	2504.8 (2275.0; 2734.6)^a^	1.256 (1.189;1.323)	11.1 (9.7; 12.4)^a^	18.0 (16.2; 19.8)^a^	3.90 (3.50; 4.30)	50.3 (47.0; 53.6)^a^	17.74 (17.02; 18.47)^a^	47.8 (44.7; 50.9)^a^	22.5 (20.8; 24.2)^a^	7.91 (7.53; 8.30)^a^
	male	11	3028.0 (2746.4; 3309.7)	1.328 (1.250;1.406)	7.4 (6.6; 8.3)	10.4 (9.5; 11.4)	2.25 (1.99; 2.51)	63.9 (60.8; 67.0)	19.33 (18.23; 20.42)	60.9 (58.0; 63.8)	29.2 (27.5; 30.9)	8.83 (8.20; 9.45)
3. Basketball	female	35	2620.4 (2447.8; 2793.0)^a^	1.220 (1.171;1.269)^a^	17.1 (15.6; 18.6)^a^	25.6 (23.9; 27.2)^a^	5.54 (5.11; 5.98)^a^	48.6 (46.8; 50.4)^a^	15.77 (15.28; 16.27)^a^	46.0 (44.3; 47.6)^a^	20.2 (19.3; 21.1)^a^	6.57 (6.31; 6.83)^a^
	male	45	3292.3 (3137.2; 3447.4)	1.299 (1.257;1.341)	12.1 (10.8; 13.3)	14.8 (13.6; 15.9)	3.32 (3.00; 3.63)	68.5 (66.0; 70.9)	18.80 (18.36; 19.25)	65.2 (62.9; 67.5)	30.9 (29.7; 32.2)	8.48 (8.24; 8.72)
4. Fencing	female	0	NA	NA	NA	NA	NA	NA	NA	NA	NA	NA
	male	0	NA	NA	NA	NA	NA	NA	NA	NA	NA	NA
5. Gymnastics	female	12	2017.8 (1807.1; 2228.5)	1.117 (1.049;1.185)	11.8 (9.6; 14.0)	22.7 (19.5; 25.8)^a^	4.59 (3.74; 5.44)	38.7 (35.9; 41.4)^a^	15.03 (14.29; 15.76)^a^	36.6 (34.0; 39.3)^a^	16.2 (15.0; 17.4)^a^	6.29 (5.96; 6.61)^a^
	male	2#	2472.4	1.190	7.8	12.0	2.79	55.0	19.71	52.5	24.5	8.76
6. Handball	female	4#	2544.0 (1849.5; 3238.5)^a^	1.270 (1.100;1.439)	18.5 (15.1; 21.9)^a^	27.3 (24.8; 29.8)	6.61 (5.39; 7.84)^a^	49.2 (44.2; 54.2)^a^	17.56 (16.13; 18.98)^a^	46.6 (42.1; 51.1)^a^	20.4 (18.0; 22.8)^a^	7.28 (6.46; 8.10)^a^
	male	37	3342.1 (3180.1; 3504.0)	1.346 (1.311;1.381)	13.6 (11.6; 15.6)	16.1 (14.4; 17.9)	4.05 (3.46; 4.63)	69.2 (66.6; 71.9)	20.52 (19.98; 21.07)	65.9 (63.4; 68.4)	31.1 (29.7; 32.4)	9.20 (8.91; 9.49)
7. Hockey Rink	female	0	NA	NA	NA	NA	NA	NA	NA	NA	NA	NA
	male	2#	2783.6	1.222	7.0	9.8	2.18	64.2	20.36	61.5	28.9	9.15
8. Korfball	female	0	NA	NA	NA	NA	NA	NA	NA	NA	NA	NA
	male	0	NA	NA	NA	NA	NA	NA	NA	NA	NA	NA
9. Modern Pentathlon	female	2#	1914.7	1.026	10.2	18.1)^a^	3.66	46.3	16.55	44.4	19.3	6.88
	male	5	2489.1 (1988.9; 2989.4)	1.159 (0.994;1.324)	8.2 (5.9; 10.5)	12.3 (9.6; 15.0)	2.58 (1.82; 3.34)	57.6 (50.6; 64.6)	18.16 (15.89; 20.42)	55.1 (48.6; 61.6)	25.5 (22.0; 29.0)	8.04 (6.95; 9.12)
10. Motorsport	female	0	NA	NA	NA	NA	NA	NA	NA	NA	NA	NA
	male	0	NA	NA	NA	NA	NA	NA	NA	NA	NA	NA
11. Other combat sports	female	4#	2184.4^a^	1.120	15.9^a^	27.6^a^	5.79^a^	42.2^a^	15.24^a^	40.0^a^	17.9^a^	6.41^a^
	male	13	2827.1 (2554.7; 3099.5)	1.261 (1.190;1.333)	9.0 (7.2; 10.8)	12.9 (11.0; 14.9)	2.89 (2.27; 3.50)	59.9 (55.4; 64.4)	19.06 (17.93; 20.19)	57.1 (52.8; 61.3)	26.4 (24.5; 28.3)	8.40 (7.93; 8.88)
12. Rowing	female	0	NA	NA	NA	NA	NA	NA	NA	NA	NA	NA
	male	6#	2861.4	1.201	10.8	14.1	3.18	65.4	19.14	62.5	29.3	8.58
13. Rugby	female	0	NA	NA	NA	NA	NA	NA	NA	NA	NA	NA
	male	39	3364.1 (3226.5; 3501.7)	1.381 (1.345;1.416)	17.1 (14.1; 20.2)	18.5 (16.3; 20.7)	5.23 (4.30; 6.16)	70.1 (66.8; 73.3)	21.33 (20.43; 22.22)	66.7 (63.6; 69.8)	31.3 (29.8; 32.7)	9.53 (9.12; 9.93)
14. Sailing	female	0	NA	NA	NA	NA	NA	NA	NA	NA	NA	NA
	male	4#	2902.5	1.246	9.1	11.8	2.74	66.7	20.27	63.8	29.5	8.95
15. Soccer	female	0	NA	NA	NA	NA	NA	NA	NA	NA	NA	NA
	male	28	2988.0 (2860.7; 3115.4)	1.341 (1.306;1.376)	8.8 (7.8; 9.7)	12.1 (11.0; 13.1)	2.82 (2.54; 3.11)	63.2 (61.0; 65.4)	20.43 (19.84; 21.01)	60.2 (58.1; 62.3)	28.1 (27.1; 29.1)	9.09 (8.80; 9.38)
15. Surf	female	1										
	male	0	NA	NA	NA	NA	NA	NA	NA	NA	NA	NA
16. Swimming	female	22	2111.8 (1992.8; 2230.7)^a^	1.095 (1.065;1.124)^a^	13.9 (12.8; 14.9)^a^	23.3 (22.1; 24.4)^a^	4.99 (4.63; 5.34)^a^	45.4 (43.1; 47.7)^a^	16.29 (15.84; 16.74)^a^	43.3 (41.1; 45.5)^a^	18.8 (17.9; 19.8)^a^	6.75 (6.56; 6.94)^a^
	male	36	2599.1 (2443.4; 2754.8)	1.167 (1.125;1.210)	8.9 (7.8; 10.0)	12.5 (11.3; 13.7)	2.76 (2.46; 3.07)	61.0 (58.5; 63.4)	18.97 (18.47; 19.48)	58.4 (56.0; 60.7)	26.7 (25.6; 27.8)	8.31 (8.07; 8.54)
17. Tennis	female	5#	2180.5^a^	1.136	16.4^a^	26.2^a^	5.84	46.6^a^	16.49^a^	44.5^a^	19.3 ^a^	6.82^a^
	male	11	2572.3 (2355.8; 2788.7)	1.197 (1.133;1.261)	12.7 (8.6; 16.7)	17.1 (13.4; 20.8)	4.06 (2.79; 5.33)	59.6 (54.0; 65.2)	19.03 (17.73; 20.32)	57.0 (51.6; 62.5)	26.8 (23.9; 29.7)	8.54 (7.86; 9.21)
18. Triathlon	female	10	1946.8 (1811.6; 2082.1)^a^	1.068 (1.021;1.114)^a^	11.4 (8.2; 14.7)^a^	20.0 (15.6; 24.4)^a^	4.17 (2.82; 5.52)^a^	44.7 (42.8; 46.6)^a^	16.10 (15.17; 17.03)^a^	42.7 (40.9; 44.6)^a^	18.8 (17.7; 20.0)^a^	6.78 (6.30; 7.26)^a^
	male	38	2448.0 (2352.1; 2544.0)	1.153 (1.123;1.183)	7.7 (7.3; 8.2)	11.9 (11.2; 12.5)	2.51 (2.34; 2.67)	57.4 (56.0; 58.8)	18.54 (18.18; 18.90)	55.0 (53.7; 56.3)	25.1 (24.3; 25.8)	8.08 (7.89; 8.28)
19. Volleyball	female	16	2518.1 (2310.4; 2725.8)^a^	1.207 (1.155;1.259)^a^	17.4 (14.5; 20.4)^a^	25.6 (23.1; 28.1)^a^	5.64 (4.90; 6.38)^a^	49.6 (46.0; 53.3)^a^	16.24 (15.52; 16.96)^a^	47.1 (43.6; 50.6)^a^	21.1 (19.1; 23.0)^a^	6.87 (6.47; 7.28)^a^
	male	17	3792.7 (3547.0; 4038.4)	1.399 (1.347;1.451)	12.9 (11.2; 14.5)	14.3 (12.6; 16.1)	3.39 (2.94; 3.84)	76.7 (73.2; 80.3)	20.18 (19.44; 20.93)	72.9 (69.6; 76.3)	35.0 (33.0; 37.0)	9.20 (8.79; 9.60)
20. Wrestling and Judo	Female	15	2389.7 (2166.8; 2612.5)^a^	1.238 (1.188;1.287)^a^	13.6 (11.1; 16.1)^a^	23.0 (19.6; 26.3)^a^	5.13 (4.24; 6.02)^a^	44.2 (40.4; 48.0)^a^	16.71 (15.58; 17.84)^a^	41.8 (38.2; 45.5)^a^	18.1 (16.3; 20.0)^a^	6.84 (6.30; 7.39)^a^
	male	45	3038.4 (2914.5; 3162.4)	1.365 (1.326;1.403)	8.7 (7.8; 9.6)	12.2 (11.3; 13.1)	2.92 (2.63; 3.21)	61.2 (58.9; 63.5)	20.66 (19.95; 21.36)	58.2 (55.9; 60.4)	26.6 (25.6; 27.7)	8.99 (8.67; 9.31)
All samples	female	143	2346.7 (2271.1; 2422.3)^a^	1.177 (1.156; 1.198)^a^	14.6 (13.9; 15.4)^a^	23.5 (22.6; 24.3)^a^	5.09 (4.86; 5.31)^a^	46.5 (45.5; 47.6)^a^	16.27 (16.01; 16.52)^a^	44.2 (43.2; 45.2)^a^	19.6 (19.0; 20.1)^a^	6.84 (6.70; 6.97)^a^
	male	339	3022.3 (2962.9; 3081.7)	1.291 (1.276; 1.306)	10.9 (10.3; 11.5)	13.9 (13.4; 14.4)	3.32 (3.15; 3.49)	64.6 (63.7; 65.6)	19.78 (19.56; 20.00)	61.6 (60.7; 62.5)	28.7 (28.3; 29.2)	8.79 (8.68; 8.89)
Sport Comparisons	female		^b)^18≠2, 19, 3; 5, 16≠3	^b)^18≠20, 2; 16≠3, 20, 2	^b)^2≠3, 19, 6; 18, 5≠3	^b)^2≠3, 19, 17, 6, 11	^b)^2≠3, 19, 6	^b)^5≠3, 19, 2	^b)^5, 3≠2	^b)^5≠3, 19, 2	^b)^5≠3, 19, 2; 20, 16≠2	^b)^5, 3, 16, 19≠2
	male		^c)^18≠15; 18, 16≠20, 3, 6, 13, 19; 9, 11≠19; 17≠3, 6, 13, 19	^c)^18≠2; 18, 16≠3, 15, 6, 20, 13, 19; 17≠20, 13, 19	^c)^2, 18, 20, 15, 16≠3, 19, 6, 13; 18≠17; 11≠13	^c)^2≠3, 6, 17, 13; 18≠6, 13; 15, 16≠13; 20≠3, 6, 13	^c)^2≠3, 6, 17, 13; 18≠3, 6, 13; 16≠6, 13; 15≠13	^c)^18, 20, 16≠3, 6, 13, 19; 17, 9, 11, 15≠19	^c)^18, 3≠15, 6, 20, 13; 16≠6, 20, 13	^c)^18, 20, 16≠3, 6, 13, 19; 9, 17, 11, 15≠19	^c)^18, 20, 16≠3, 6, 13, 19; 9, 17, 15≠19; 11≠3, 6, 19	^c)^18≠20, 15, 19, 6, 13; 16≠15, 6, 13; 3≠6, 13

Abbreviations: WB, whole body; BMC, bone mineral content; BMD, bone mineral density; FM, fat mass; FMI, fat mass index; FFM, fat-free mass; FFMI, fat-free mass index; LST, lean soft tissue; ALST, appendicular lean soft tissue.

# 95% confident intervals were not presented for n<8. NA: data not presented for n<2. ^a)^Significant different from males (p<0.05); ^b)^Significant differences between sports for females (p<0.05); ^c)^ Significant differences between sports for males (p<0.05).

## Discussion

Despite the recognized importance of body composition for athletic performance [1], [2], [3] and health.[9] appropriate reference values for the athletic population have been lacking. In this study, we developed sex- and sport-specific percentiles for body composition at both a molecular and a whole-body level by use of DXA and anthropometry, respectively.

DXA is a widespread method for the assessment of the athletic population because it permits the acquisition of both regional and total body composition without the need for costly or scarce medical imaging techniques.[1], [13] Concerning DXA outcomes, we have presented the 5^th^, 25^th^, 50^th^, 75^th^, and 90^th^ percentiles for total (whole-body scan) and regional (subtotal, trunk, and appendicular) body composition, including measures of BMD, BMC, absolute and percentage FM, FFM, and LST. Additionally, we also presented reference percentiles for height-normalized indexes such as BMI by dividing FM, FFM, and ALST by height squared. This approach has been suggested to allow comparisons among individuals;[26] in addition, reference values for the adult population have already been developed.[17] The percentiles for DXA measurements are presented in SI2. To aid in interpreting these percentiles, we give an example for a male handball player who has an FM of 13.0%. In SI2 (table 5) we can see that the estimate for the 5^th^ percentile is 8.0% (95% CI: 3.3–11.7%), the estimate for the 25^th^ percentile is 12.9% (95% CI: 9.7–15.7%), and the estimate for the 50^th^ percentile is 16.3% (95% CI: 14.2–18.5%). Thus, the male handball player falls in the bottom half of the distribution but is not in the lowest 5% of that distribution; we estimate that his FM measurement is at about the 25^th^ percentile for his sport.

Athletes as a rule have a lower %FM than do nonathletes of the same chronologic age.[3] An excess of FM may have a negative impact on sports performance and is often viewed as a major limiting factor in athletic achievements.[3] Alternatively, in athletics, a lower percentage of FM may be related to several heath complications of concern to sports professionals.[9] Malina et al.[3] reviewed fatness estimates of athletes from different sports; however most of the results were based on densitometric methods to estimate FM. These methods assume an established value of 1.1 g/cm^3^ for FFM density but observed deviations from this constant may occur.[27] In the present investigation, we developed reference percentiles by using DXA, a 3-compartment method that is a reliable and valid alternative to other recognized methods owing to its speed and convenience.[13]

It has been shown that athletes have a larger FFM than do nonathletes. Much of these differences may be related to an increased skeletal muscle mass.[20] A relatively large fraction of total-body skeletal muscle mass is in the appendages, and a high percentage of ALST is skeletal mass. As such, estimation of ALST by DXA is a potentially practical and accurate method of quantifying human skeletal mass in vivo.[28], [29] On the other hand, ALST divided by height^2^ (ALSTI) has been suggested as a proxy index for sarcopenia.[30] It is expected that an athletic population, particularly those engaged in high intensity training, would present an increased skeletal mass compared with nonathletes.[31] In this study, in addition to whole-body FFM and LST, we presented quantiles for both ALST and ALSTI for each sex and separately for several sports for which athletes' body composition profiles may differ.

Another advantage of using DXA in the athletic population is its ability to assess BMD. The female athlete triad syndrome (FTS) position of the American College of Sports Medicine (ACSM) states that BMD should be assessed with DXA whenever the athlete presents a history of stress fractures or fractures from minimal trauma.[9] In our study, we observed that BMD was lower among triathlon and swimming athletes. FTS is seen more often in endurance sports like triathlon along with sports that emphasize thinness (e.g., gymnastics, figure skating, and dancing). On the other hand, it has been suggested that a higher peak bone mass may be achieved by regularly performing weight-bearing exercises, particularly if associated with impacts.[32]

Body composition disturbances are an important issue in sports medicine. A position statement under the auspices of the IOC Medical Commission has acknowledged this concern and recommends that skinfolds or DXA are used to monitor changes in body composition with interventions that seek to induce weight loss or change body composition in athletes. [10] In our work we do not provide evidence based critical values associated with health complications. However, by providing sport specific percentiles using the above mentioned techniques we can help sports professionals understand if their athletes are in the bottom of the distribution, which may lead to severe health problems.

Despite its advantages, however, DXA may not be practical for field assessment. Furthermore, caution is necessary when using this method on multiple occasions, perhaps no more than four times per year,[1] not only because of the cumulative radiation dose but also because the error of measurement in detecting small changes in body composition [33] may lead to misinterpretation of data. Anthropometry provides a simple, relatively inexpensive, and non-invasive field method for estimating body composition.[1], [34] Thus, we have also presented percentiles for anthropometry outcomes (SI1). The interpretation of the percentiles for these variables is similar to the example given for %FM from DXA.

Inconsistency exists when using anthropometry to estimate molecular body composition compartments, particularly in the athletic population, because the equations rely on assumptions that may not be valid in athletes.[27], [35] The other source of error is the lack of standardization for the measurement of skinfolds and circumferences.[1] In order to solve these common issues, we obtained anthropometric data by using a standardized protocol.[20] On the other hand, by using the raw data instead of applying an equation, i.e., the use of sum of skinfolds (∑7SKF, ∑_app_SKF, ∑_trunk_SKF, ∑_arm_SKF, and ∑_leg_SKF) and body circumferences (hip, arm, midthigh, calf, and abdominal circumferences), we were able to reduce errors by avoiding assumptions that may not be valid in the athletic population. In this regard, Ackland et al. [1] and Marfell-Jones [36] highlighted the importance of individual and sums of skinfold thicknesses as valid proxy measurements of adiposity.

Although circumferences are not often used to assess body composition in the athletic population, it seems likely that muscle circumferences may be a useful anthropometric tool as a representation of skeletal muscle mass.[37] In this study, following the procedure suggested by Heymsfield et al.,[22] we included reference quantiles for arm, thigh, and calf muscle circumference in addition to the unadjusted measures for the skinfold thicknesses. At this regard it is important to mention that, although muscle circumferences offer an excellent field approach to muscle mass, there are a few limitations and inaccuracies associated, as the tissues (skin and subcutaneous adipose tissue) compressed when measuring skinfolds and compressibility depends largely on the anatomical site and on the person that is being assessed [38].

### Study limitations

The DXA reference values presented in this study are only comparable with those derived by use of the Hologic fan beam DXA scanner (software version 12.4 or higher). Another limitation of this investigation is that owing to the small sample size in each sport. The empirical Bayesian framework we employed allows us to ‘share’ information across sports whenever there are at least two individuals with recorded outcome measurements for a given sport. The benefit accrued from that communication strongly depends on the nature of the values for the other sports as well as the number of individuals measured for that specific sport. It may be helpful for the reader to think of any given calculation as a weighted estimate between the information available for a given sport and the other sports, with the weight for the latter depending on how consistent their values are across sports, ranging from the high single digits down to less than a tenth of a person. Therefore, if there are many individuals measured for a given sport (e.g., male rugby) their information will dominate that of the other sports. Conversely, estimates for sports with low sample sizes will be strongly influenced by the others. For this reason, we do not present confidence intervals and reference values for estimates when n<8. Additionally, we were not able to present position-specific reference percentiles for each body composition outcome. It is important to reinforce that the “athletics” sport comprised only sprinters, hurdlers, and jumpers (long and triple jump). Finally, ethnic variation should also be considered as a limitation of this study given that variation exists in body proportions and composition.[39]

## Conclusions

This study provides reference body composition percentiles for athletes by sex and sport. Sports professionals will benefit from using these reference percentiles for assessing and classifying body composition in athletes. We used DXA to derive total and regional body composition reference values. In addition, given their applicability in the field setting, we also used anthropometric methods (sum of skinfolds, circumferences, and muscle circumferences) to develop reference percentiles for whole-body composition. These reference values should be helpful in the evaluation of athletes from different sports regarding not only performance but also other health-related criteria and to establish directions for future research.

## Supporting Information

File S1 **Anthropometry variables percentiles by sport and sex.**
(PDF)

File S2 **Dual Energy X-ray Absorptiometry variables percentiles by sport and sex.**
(PDF)
