# Supplementary material for: Reference Values for Body Composition and Anthropometric Measurements in Athletes
Source: PLoS One. 2014 May 15;9(5):e97846. doi: 10.1371/journal.pone.0097846 (PMC4022746; doi:10.1371/journal.pone.0097846)
Supplement: File S1 — Anthropometry variables percentiles by sport and sex. (PDF) [file pone.0097846.s001.pdf]

Supporting Information 1 (SI1):

Anthropometry variables percentiles by sport and sex

Contents

Table 1 – Body weight (kg) percentiles by sport and sex 1
Table 2 – Height (cm) percentiles by sport and sex 2
Table 3 – Body mass index (kg/m^2) percentiles by sport and sex 3
Table 4 – Sum of seven skinfolds [triceps + subscapular + biceps + suprailiac + abdominal + thigh + medial calf (mm)] percentiles by sport and sex 4
Table 5 – Sum of appendicular skinfolds [triceps + biceps + thigh + medial calf (mm)] percentiles by sport and ..5
Table 7 – Sum of leg skinfolds [thigh + medial calf (mm)] percentiles by sport and sex 7
Table 8 – Sum of trunk skinfolds [subscapular + suprailiac + abdominal (mm)] percentiles by sport and sex........8
Table 9 – Arm circumference (cm) percentiles by sport and sex 9
Table 10 – Arm muscle circumference (cm) percentiles by sport and sex....10
Table 11 – Thigh circumference (cm) percentiles by sport and sex....11
Table 12 – Thigh muscle circumference (cm) percentiles by sport and sex....12
Table 13 – Calf circumference (cm) percentiles by sport and sex....13
Table 14 – Calf muscle circumference (cm) percentiles by sport and sex 14
Table 15 – Abdominal circumference (cm) percentiles by sport and sex 15
Table 16 – Hip circumference (cm) percentiles by sport and sex 16

Table 1 – Body weight (kg) percentiles by sport and sex

Sport	0.05			0.25			Median			0.75			0.95		
	Low	Estimate	High	Low	Estimate	High	Low	Estimate	High	Low	Estimate	High	Low	Estimate	High
**Females**															
Archery and Shooting	NA	**NA**	NA	NA	**NA**	NA	NA	**NA**	NA	NA	**NA**	NA	NA	**NA**	NA
Athletics	42.49	**48.54**	53.56	50.43	**54.80**	58.76	55.94	**59.16**	62.37	59.56	**63.51**	67.89	64.75	**69.77**	75.82
Basketball	47.24	**54.32**	60.25	57.73	**62.81**	67.42	65.03	**68.71**	72.40	70.00	**74.61**	79.69	77.17	**83.11**	90.18
Fencing	NA	**NA**	NA	NA	**NA**	NA	NA	**NA**	NA	NA	**NA**	NA	NA	**NA**	NA
Gymnastics	35.66	**43.30**	49.45	43.87	**49.42**	54.36	49.58	**53.67**	57.77	52.99	**57.93**	63.48	57.90	**64.05**	71.69
Handball	NA	**NA**	NA	NA	**NA**	NA	NA	**NA**	NA	NA	**NA**	NA	NA	**NA**	NA
Hockey Rink	NA	**NA**	NA	NA	**NA**	NA	NA	**NA**	NA	NA	**NA**	NA	NA	**NA**	NA
Korfball	37.76	**48.23**	56.53	46.73	**54.51**	61.40	52.97	**58.87**	64.77	56.35	**63.24**	71.01	61.21	**69.52**	79.98
Modern Pentathlon	35.92	**48.29**	58.09	46.60	**55.76**	63.86	54.03	**60.95**	67.87	58.04	**66.14**	75.30	63.81	**73.61**	85.98
Motorsport	NA	**NA**	NA	NA	**NA**	NA	NA	**NA**	NA	NA	**NA**	NA	NA	**NA**	NA
Other combat sports	39.87	**48.44**	55.32	48.50	**54.77**	60.35	54.50	**59.17**	63.84	57.99	**63.57**	69.84	63.02	**69.90**	78.47
Rowing	43.62	**54.80**	63.68	52.81	**61.17**	68.59	59.20	**65.60**	72.01	62.61	**70.03**	78.39	67.53	**76.41**	87.58
Rugby	NA	**NA**	NA	NA	**NA**	NA	NA	**NA**	NA	NA	**NA**	NA	NA	**NA**	NA
Sailing	NA	**NA**	NA	NA	**NA**	NA	NA	**NA**	NA	NA	**NA**	NA	NA	**NA**	NA
Soccer	41.64	**48.98**	54.95	50.05	**55.37**	60.14	55.89	**59.82**	63.74	59.50	**64.26**	69.59	64.69	**70.66**	78.00
Surf	NA	**NA**	NA	NA	**NA**	NA	NA	**NA**	NA	NA	**NA**	NA	NA	**NA**	NA
Swimming	43.45	**49.61**	54.68	50.91	**55.38**	59.41	56.09	**59.39**	62.69	59.37	**63.40**	67.88	64.10	**69.17**	75.33
Tennis	43.34	**53.20**	61.08	52.27	**59.58**	66.06	58.49	**64.01**	69.53	61.95	**68.44**	75.74	66.94	**74.82**	84.68
Triathlon	37.38	**47.31**	55.20	46.46	**53.77**	60.25	52.77	**58.26**	63.76	56.28	**62.75**	70.07	61.33	**69.21**	79.14
Volleyball	40.29	**52.05**	61.45	52.57	**61.08**	68.63	61.10	**67.36**	73.62	66.09	**73.64**	82.15	73.28	**82.67**	94.43
Wrestling and Judo	37.48	**46.11**	53.10	47.80	**54.01**	59.54	54.97	**59.51**	64.01	59.45	**65.01**	71.18	65.88	**72.91**	81.51
**Males**															
Archery and Shooting	41.70	**58.63**	71.01	55.50	**67.45**	77.55	65.08	**73.59**	82.09	69.63	**79.72**	91.68	76.17	**88.55**	105.48
Athletics	55.77	**62.83**	68.53	64.40	**69.44**	73.92	70.41	**74.03**	77.66	74.15	**78.62**	83.66	79.53	**85.23**	92.29
Basketball	57.13	**65.17**	71.87	69.26	**74.95**	80.09	77.70	**81.76**	85.81	83.42	**88.56**	94.25	91.65	**98.34**	106.38
Fencing	47.92	**60.40**	69.73	58.98	**67.81**	75.35	66.67	**72.96**	79.26	70.57	**78.11**	86.94	76.20	**85.52**	98.00
Gymnastics	44.43	**53.99**	61.43	54.58	**61.33**	67.22	61.63	**66.44**	71.24	65.66	**71.54**	78.29	71.45	**78.88**	88.44
Handball	56.62	**66.37**	74.29	69.69	**76.56**	82.69	78.77	**83.65**	88.53	84.61	**90.74**	97.61	93.01	**100.93**	110.68
Hockey Rink	55.33	**61.73**	67.07	64.97	**69.52**	73.64	71.67	**74.94**	78.21	76.23	**80.36**	84.91	82.80	**88.15**	94.55
Korfball	46.04	**59.62**	69.71	57.74	**67.35**	75.54	65.88	**72.73**	79.59	69.93	**78.11**	87.72	75.76	**85.85**	99.43
Modern Pentathlon	43.31	**55.86**	65.34	55.09	**63.94**	71.54	63.28	**69.56**	75.84	67.59	**75.18**	84.04	73.79	**83.27**	95.82
Motorsport	NA	**NA**	NA	NA	**NA**	NA	NA	**NA**	NA	NA	**NA**	NA	NA	**NA**	NA
Other combat sports	48.49	**56.58**	63.13	59.06	**64.77**	69.86	66.41	**70.47**	74.53	71.08	**76.16**	81.88	77.81	**84.36**	92.44
Rowing	54.76	**64.18**	71.69	65.98	**72.64**	78.51	73.79	**78.52**	83.26	78.53	**84.40**	91.06	85.35	**92.86**	102.28
Rugby	55.97	**66.58**	75.53	74.12	**81.56**	88.32	86.73	**91.97**	97.21	95.62	**102.38**	109.82	108.41	**117.37**	127.97
Sailing	46.73	**57.09**	65.52	61.09	**68.35**	74.82	71.08	**76.18**	81.28	77.54	**84.01**	91.27	86.84	**95.27**	105.63
Soccer	54.43	**61.14**	66.68	63.90	**68.67**	72.96	70.48	**73.90**	77.33	74.85	**79.14**	83.91	81.13	**86.67**	93.37
Surf	NA	**NA**	NA	NA	**NA**	NA	NA	**NA**	NA	NA	**NA**	NA	NA	**NA**	NA
Swimming	50.65	**57.91**	63.93	61.18	**66.33**	70.96	68.50	**72.18**	75.85	73.39	**78.02**	83.17	80.42	**86.44**	93.70
Tennis	45.32	**56.22**	64.75	57.61	**65.27**	71.96	66.15	**71.56**	76.97	71.16	**77.85**	85.51	78.37	**86.90**	97.80
Triathlon	52.60	**57.34**	61.26	59.16	**62.55**	65.60	63.73	**66.17**	68.62	66.74	**69.79**	73.18	71.08	**75.00**	79.74
Volleyball	65.18	**76.29**	84.88	76.15	**84.06**	90.94	83.77	**89.46**	95.15	87.98	**94.86**	102.77	94.04	**102.63**	113.74
Wrestling and Judo	50.18	**56.27**	61.48	60.98	**65.29**	69.24	68.49	**71.56**	74.64	73.89	**77.83**	82.15	81.65	**86.85**	92.95

NA: data not presented for n < 8.

Table 2 – Height (cm) percentiles by sport and sex

Sport	0.05			0.25			Median			0.75			0.95		
	Low	Estimate	High	Low	Estimate	High	Low	Estimate	High	Low	Estimate	High	Low	Estimate	High
**Females**															
**Archery and Shooting**	NA	**NA**	NA	NA	**NA**	NA	NA	**NA**	NA	NA	**NA**	NA	NA	**NA**	NA
**Athletics**	149.8	**155.3**	160.2	157.4	**161.7**	165.7	162.6	**166.1**	169.6	166.4	**170.5**	174.8	172.0	**176.9**	182.3
**Basketball**	158.3	**163.9**	168.9	166.9	**171.2**	175.3	172.9	**176.3**	179.8	177.3	**181.4**	185.8	183.7	**188.8**	194.4
**Fencing**	NA	**NA**	NA	NA	**NA**	NA	NA	**NA**	NA	NA	**NA**	NA	NA	**NA**	NA
**Gymnastics**	142.8	**149.7**	155.9	150.5	**156.1**	161.4	155.9	**160.6**	165.3	159.8	**165.1**	170.7	165.3	**171.5**	178.4
**Handball**	NA	**NA**	NA	NA	**NA**	NA	NA	**NA**	NA	NA	**NA**	NA	NA	**NA**	NA
**Hockey Rink**	NA	**NA**	NA	NA	**NA**	NA	NA	**NA**	NA	NA	**NA**	NA	NA	**NA**	NA
**Korfball**	141.5	**150.6**	158.8	149.7	**157.3**	164.5	155.4	**162.0**	168.5	159.4	**166.6**	174.2	165.1	**173.4**	182.5
**Modern Pentathlon**	149.3	**158.3**	166.5	157.3	**164.8**	172.0	162.9	**169.4**	175.9	166.8	**174.0**	181.5	172.3	**180.5**	189.5
**Motorsport**	NA	**NA**	NA	NA	**NA**	NA	NA	**NA**	NA	NA	**NA**	NA	NA	**NA**	NA
**Other combat sports**	144.5	**151.8**	158.3	152.3	**158.1**	163.7	157.6	**162.6**	167.5	161.4	**167.0**	172.9	166.9	**173.4**	180.6
**Rowing**	148.5	**157.7**	166.0	156.4	**164.1**	171.5	161.9	**168.6**	175.3	165.7	**173.1**	180.8	171.1	**179.5**	188.7
**Rugby**	NA	**NA**	NA	NA	**NA**	NA	NA	**NA**	NA	NA	**NA**	NA	NA	**NA**	NA
**Sailing**	NA	**NA**	NA	NA	**NA**	NA	NA	**NA**	NA	NA	**NA**	NA	NA	**NA**	NA
**Soccer**	147.1	**153.3**	158.9	154.6	**159.5**	164.2	159.7	**163.8**	167.9	163.4	**168.1**	173.1	168.8	**174.3**	180.5
**Surf**	NA	**NA**	NA	NA	**NA**	NA	NA	**NA**	NA	NA	**NA**	NA	NA	**NA**	NA
**Swimming**	150.8	**156.7**	162.0	158.3	**162.9**	167.4	163.5	**167.3**	171.2	167.2	**171.7**	176.4	172.7	**178.0**	183.8
**Tennis**	148.9	**156.9**	164.2	156.7	**163.3**	169.6	162.0	**167.7**	173.3	165.8	**172.1**	178.7	171.1	**178.4**	186.4
**Triathlon**	148.1	**156.5**	164.0	156.1	**163.0**	169.5	161.7	**167.6**	173.4	165.6	**172.1**	179.0	171.1	**178.6**	187.0
**Volleyball**	152.6	**160.9**	168.4	161.7	**168.4**	174.7	168.0	**173.6**	179.2	172.4	**178.8**	185.5	178.8	**186.3**	194.5
**Wrestling and Judo**	144.8	**150.9**	156.5	152.5	**157.4**	162.1	157.9	**161.9**	166.0	161.8	**166.4**	171.4	167.4	**172.9**	179.1
**Males**															
**Archery and Shooting**	157.6	**166.7**	174.6	165.2	**172.5**	179.3	170.5	**176.5**	182.6	173.8	**180.6**	187.9	178.5	**186.4**	195.5
**Athletics**	165.6	**171.5**	176.7	173.0	**177.5**	181.7	178.0	**181.6**	185.2	181.5	**185.8**	190.3	186.6	**191.7**	197.6
**Basketball**	168.5	**175.4**	181.4	179.0	**184.2**	189.0	186.3	**190.3**	194.3	191.5	**196.3**	201.5	199.1	**205.1**	212.0
**Fencing**	159.9	**168.6**	176.0	167.9	**174.7**	181.0	173.4	**179.0**	184.5	176.9	**183.2**	190.0	181.9	**189.4**	198.0
**Gymnastics**	153.0	**159.8**	165.7	160.4	**165.7**	170.6	165.5	**169.8**	174.0	168.9	**173.9**	179.2	173.8	**179.7**	186.6
**Handball**	166.6	**172.2**	177.1	174.1	**178.4**	182.3	179.3	**182.6**	186.0	182.9	**186.9**	191.2	188.1	**193.0**	198.6
**Hockey Rink**	161.2	**165.5**	169.3	167.7	**170.9**	173.9	172.2	**174.7**	177.2	175.4	**178.4**	181.7	180.1	**183.9**	188.1
**Korfball**	160.1	**169.3**	177.2	168.3	**175.6**	182.4	174.1	**180.0**	186.0	177.7	**184.4**	191.7	182.8	**190.8**	199.9
**Modern Pentathlon**	158.4	**166.2**	173.0	165.9	**172.1**	177.8	171.2	**176.2**	181.2	174.5	**180.3**	186.4	179.4	**186.1**	194.0
**Motorsport**	NA	**NA**	NA	NA	**NA**	NA	NA	**NA**	NA	NA	**NA**	NA	NA	**NA**	NA
**Other combat sports**	159.8	**165.4**	170.3	167.2	**171.4**	175.4	172.3	**175.6**	178.9	175.8	**179.8**	184.0	180.9	**185.8**	191.4
**Rowing**	166.0	**172.1**	177.5	173.5	**178.2**	182.6	178.7	**182.4**	186.1	182.2	**186.6**	191.3	187.3	**192.7**	198.8
**Rugby**	165.6	**170.6**	175.0	173.9	**177.7**	181.1	179.7	**182.6**	185.4	184.0	**187.5**	191.2	190.2	**194.6**	199.6
**Sailing**	159.6	**165.7**	171.1	168.1	**172.7**	177.1	174.0	**177.6**	181.2	178.1	**182.5**	187.1	184.1	**189.5**	195.6
**Soccer**	161.6	**166.5**	170.8	168.6	**172.3**	175.8	173.5	**176.4**	179.3	177.0	**180.4**	184.2	182.0	**186.3**	191.2
**Surf**	NA	**NA**	NA	NA	**NA**	NA	NA	**NA**	NA	NA	**NA**	NA	NA	**NA**	NA
**Swimming**	163.0	**168.4**	173.1	170.9	**175.0**	178.8	176.4	**179.6**	182.8	180.4	**184.2**	188.3	186.1	**190.8**	196.3
**Tennis**	160.0	**166.6**	172.4	167.6	**172.7**	177.5	172.9	**177.0**	181.1	176.5	**181.2**	186.4	181.6	**187.3**	194.0
**Triathlon**	161.0	**165.9**	170.1	167.9	**171.6**	175.0	172.7	**175.6**	178.5	176.1	**179.6**	183.3	181.0	**185.3**	190.2
**Volleyball**	171.9	**180.9**	188.7	181.3	**188.3**	194.8	187.8	**193.4**	199.0	192.0	**198.5**	205.5	198.1	**205.8**	214.9
**Wrestling and Judo**	158.0	**162.1**	165.8	165.2	**168.3**	171.2	170.2	**172.6**	174.9	174.0	**176.9**	179.9	179.4	**183.0**	187.2

NA: data not presented for n < 8.

Table 3 – Body mass index (kg/m^2) percentiles by sport and sex

**Sport**	**0.05**			**0.25**			**Median**			**0.75**			**0.95**		
	**Low**	**Estimate**	**High**	**Low**	**Estimate**	**High**	**Low**	**Estimate**	**High**	**Low**	**Estimate**	**High**	**Low**	**Estimate**	**High**
**Females**															
**Archery and Shooting**	NA	**NA**	NA	NA	**NA**	NA	NA	**NA**	NA	NA	**NA**	NA	NA	**NA**	NA
**Athletics**	17.32	**18.60**	19.76	19.21	**20.22**	21.18	20.52	**21.34**	22.16	21.51	**22.46**	23.47	22.92	**24.08**	25.37
**Basketball**	17.59	**18.89**	20.06	19.72	**20.72**	21.68	21.20	**22.00**	22.80	22.32	**23.27**	24.28	23.94	**25.11**	26.40
**Fencing**	NA	**NA**	NA	NA	**NA**	NA	NA	**NA**	NA	NA	**NA**	NA	NA	**NA**	NA
**Gymnastics**	16.12	**17.78**	19.28	18.15	**19.48**	20.75	19.56	**20.67**	21.78	20.58	**21.85**	23.19	22.06	**23.56**	25.21
**Handball**	NA	**NA**	NA	NA	**NA**	NA	NA	**NA**	NA	NA	**NA**	NA	NA	**NA**	NA
**Hockey Rink**	NA	**NA**	NA	NA	**NA**	NA	NA	**NA**	NA	NA	**NA**	NA	NA	**NA**	NA
**Korfball**	17.12	**19.27**	21.25	19.22	**21.02**	22.74	20.68	**22.23**	23.78	21.72	**23.44**	25.24	23.21	**25.18**	27.34
**Modern Pentathlon**	15.57	**17.84**	19.93	17.81	**19.70**	21.51	19.36	**20.99**	22.62	20.46	**22.28**	24.17	22.05	**24.13**	26.41
**Motorsport**	NA	**NA**	NA	NA	**NA**	NA	NA	**NA**	NA	NA	**NA**	NA	NA	**NA**	NA
**Other combat sports**	17.31	**19.19**	20.89	19.47	**21.00**	22.46	20.98	**22.26**	23.54	22.06	**23.52**	25.04	23.62	**25.33**	27.20
**Rowing**	17.73	**19.93**	21.94	19.78	**21.62**	23.39	21.20	**22.80**	24.40	22.20	**23.97**	25.82	23.65	**25.67**	27.86
**Rugby**	NA	**NA**	NA	NA	**NA**	NA	NA	**NA**	NA	NA	**NA**	NA	NA	**NA**	NA
**Sailing**	NA	**NA**	NA	NA	**NA**	NA	NA	**NA**	NA	NA	**NA**	NA	NA	**NA**	NA
**Soccer**	17.56	**19.17**	20.62	19.66	**20.94**	22.16	21.12	**22.17**	23.23	22.19	**23.40**	24.69	23.72	**25.18**	26.78
**Surf**	NA	**NA**	NA	NA	**NA**	NA	NA	**NA**	NA	NA	**NA**	NA	NA	**NA**	NA
**Swimming**	16.91	**18.32**	19.59	18.87	**19.98**	21.04	20.22	**21.13**	22.04	21.23	**22.28**	23.40	22.67	**23.94**	25.35
**Tennis**	17.67	**19.65**	21.45	19.72	**21.36**	22.92	21.15	**22.55**	23.94	22.17	**23.74**	25.37	23.63	**25.45**	27.42
**Triathlon**	15.56	**17.58**	19.44	17.69	**19.35**	20.96	19.17	**20.59**	22.02	20.22	**21.82**	23.50	21.74	**23.59**	25.63
**Volleyball**	17.29	**19.08**	20.71	19.40	**20.85**	22.24	20.87	**22.09**	23.30	21.94	**23.32**	24.77	23.47	**25.09**	26.89
**Wrestling and Judo**	17.79	**19.40**	20.85	19.96	**21.24**	22.45	21.47	**22.52**	23.56	22.59	**23.80**	25.07	24.19	**25.64**	27.25
**Males**															
**Archery and Shooting**	13.06	**18.62**	22.67	17.58	**21.50**	24.79	20.71	**23.49**	26.27	22.19	**25.49**	29.41	24.31	**28.36**	33.93
**Athletics**	17.73	**19.54**	21.00	19.93	**21.22**	22.37	21.45	**22.39**	23.32	22.40	**23.55**	24.85	23.77	**25.23**	27.04
**Basketball**	17.63	**19.24**	20.60	20.02	**21.17**	22.22	21.68	**22.52**	23.35	22.81	**23.86**	25.01	24.44	**25.79**	27.40
**Fencing**	16.02	**19.34**	21.83	18.92	**21.28**	23.31	20.94	**22.63**	24.33	21.96	**23.98**	26.34	23.43	**25.92**	29.24
**Gymnastics**	15.54	**18.79**	21.32	18.98	**21.28**	23.28	21.37	**23.00**	24.63	22.73	**24.73**	27.03	24.69	**27.21**	30.47
**Handball**	18.68	**20.97**	22.85	21.70	**23.33**	24.79	23.80	**24.97**	26.14	25.15	**26.61**	28.24	27.09	**28.96**	31.25
**Hockey Rink**	18.86	**20.71**	22.26	21.63	**22.95**	24.15	23.55	**24.51**	25.46	24.87	**26.07**	27.39	26.75	**28.31**	30.16
**Korfball**	14.70	**18.60**	21.49	18.03	**20.79**	23.14	20.35	**22.32**	24.29	21.50	**23.85**	26.60	23.15	**26.04**	29.94
**Modern Pentathlon**	14.57	**18.29**	21.09	18.03	**20.66**	22.91	20.44	**22.30**	24.17	21.70	**23.95**	26.57	23.51	**26.32**	30.03
**Motorsport**	NA	**NA**	NA	NA	**NA**	NA	NA	**NA**	NA	NA	**NA**	NA	NA	**NA**	NA
**Other combat sports**	16.85	**19.05**	20.83	19.69	**21.25**	22.63	21.66	**22.77**	23.89	22.91	**24.30**	25.86	24.72	**26.50**	28.70
**Rowing**	17.88	**20.14**	21.94	20.52	**22.13**	23.54	22.36	**23.51**	24.66	23.48	**24.89**	26.50	25.08	**26.89**	29.14
**Rugby**	17.52	**20.48**	22.98	22.55	**24.64**	26.53	26.05	**27.52**	29.00	28.51	**30.41**	32.49	32.06	**34.56**	37.53
**Sailing**	17.29	**19.68**	21.63	20.53	**22.22**	23.74	22.79	**23.99**	25.20	24.25	**25.76**	27.45	26.36	**28.31**	30.69
**Soccer**	18.85	**20.53**	21.93	21.18	**22.39**	23.48	22.81	**23.68**	24.56	23.88	**24.97**	26.18	25.44	**26.83**	28.52
**Surf**	NA	**NA**	NA	NA	**NA**	NA	NA	**NA**	NA	NA	**NA**	NA	NA	**NA**	NA
**Swimming**	17.52	**19.14**	20.50	19.84	**21.00**	22.05	21.45	**22.29**	23.13	22.53	**23.58**	24.74	24.08	**25.44**	27.06
**Tennis**	15.58	**18.58**	20.94	18.92	**21.04**	22.90	21.25	**22.75**	24.26	22.61	**24.46**	26.59	24.57	**26.93**	29.93
**Triathlon**	17.45	**18.85**	20.02	19.37	**20.38**	21.29	20.70	**21.44**	22.17	21.59	**22.50**	23.51	22.86	**24.02**	25.43
**Volleyball**	18.30	**20.85**	22.82	20.79	**22.61**	24.19	22.53	**23.84**	25.15	23.48	**25.06**	26.88	24.85	**26.82**	29.37
**Wrestling and Judo**	18.30	**19.93**	21.32	21.14	**22.31**	23.37	23.12	**23.96**	24.80	24.55	**25.61**	26.78	26.59	**27.99**	29.62

NA: data not presented for n < 8.

Table 4 – Sum of seven skinfolds [triceps + subscapular + biceps + suprailiac + abdominal + thigh + medial calf (mm)] percentiles by sport and sex

Sport	0.05			0.25			Median			0.75			0.95		
	Low	Estimate	High	Low	Estimate	High	Low	Estimate	High	Low	Estimate	High	Low	Estimate	High
**Females**															
**Archery and Shooting**	NA	**NA**	NA	NA	**NA**	NA	NA	**NA**	NA	NA	**NA**	NA	NA	**NA**	NA
**Athletics**	1.71	**27.21**	48.22	35.86	**53.97**	70.25	59.59	**72.57**	85.55	74.90	**91.18**	109.29	96.92	**117.94**	143.43
**Basketball**	43.84	**70.14**	92.33	84.45	**103.20**	120.25	112.68	**126.17**	139.66	132.09	**149.15**	167.90	160.02	**182.21**	208.51
**Fencing**	NA	**NA**	NA	NA	**NA**	NA	NA	**NA**	NA	NA	**NA**	NA	NA	**NA**	NA
**Gymnastics**	18.79	**47.46**	71.11	52.94	**73.76**	92.52	76.68	**92.04**	107.40	91.56	**110.32**	131.14	112.97	**136.62**	165.29
**Handball**	NA	**NA**	NA	NA	**NA**	NA	NA	**NA**	NA	NA	**NA**	NA	NA	**NA**	NA
**Hockey Rink**	NA	**NA**	NA	NA	**NA**	NA	NA	**NA**	NA	NA	**NA**	NA	NA	**NA**	NA
**Korfball**	23.32	**64.25**	98.02	62.46	**93.21**	121.02	89.67	**113.34**	137.01	105.66	**133.47**	164.22	128.66	**162.42**	203.36
**Modern Pentathlon**	0.05	**41.22**	75.02	38.48	**69.30**	97.14	65.18	**88.82**	112.52	80.56	**108.34**	139.23	102.68	**136.42**	177.65
**Motorsport**	NA	**NA**	NA	NA	**NA**	NA	NA	**NA**	NA	NA	**NA**	NA	NA	**NA**	NA
**Other combat sports**	12.71	**53.82**	87.53	54.74	**85.16**	112.53	83.96	**106.94**	129.91	101.34	**128.72**	159.13	126.35	**160.05**	201.17
**Rowing**	19.24	**58.85**	91.47	55.78	**85.70**	112.71	81.17	**104.36**	127.48	95.94	**123.02**	152.87	117.18	**149.87**	189.41
**Rugby**	NA	**NA**	NA	NA	**NA**	NA	NA	**NA**	NA	NA	**NA**	NA	NA	**NA**	NA
**Sailing**	NA	**NA**	NA	NA	**NA**	NA	NA	**NA**	NA	NA	**NA**	NA	NA	**NA**	NA
**Soccer**	31.47	**59.08**	82.01	66.41	**86.42**	104.50	90.71	**105.42**	120.13	106.34	**124.42**	144.42	128.83	**151.76**	179.37
**Surf**	NA	**NA**	NA	NA	**NA**	NA	NA	**NA**	NA	NA	**NA**	NA	NA	**NA**	NA
**Swimming**	22.57	**47.92**	68.95	56.64	**74.81**	91.20	80.32	**93.50**	106.67	95.79	**112.19**	130.35	118.05	**139.08**	164.42
**Tennis**	51.97	**90.25**	122.02	89.31	**118.17**	144.37	115.26	**137.58**	159.90	130.79	**156.99**	185.85	153.13	**184.91**	223.18
**Triathlon**	0.00	**35.11**	74.53	30.27	**66.54**	99.11	60.50	**88.38**	116.20	77.59	**110.22**	146.43	102.17	**141.65**	189.92
**Volleyball**	30.00	**65.10**	94.04	70.08	**95.76**	118.93	97.93	**117.08**	136.23	115.23	**138.39**	164.08	140.11	**169.06**	204.15
**Wrestling and Judo**	29.14	**64.21**	93.23	73.03	**98.38**	121.24	103.53	**122.12**	140.71	123.00	**145.87**	171.22	151.02	**180.04**	215.11
**Males**															
**Archery and Shooting**	0.00	**45.84**	81.12	35.95	**71.63**	99.16	67.40	**89.55**	111.70	79.95	**107.48**	143.15	97.99	**133.26**	188.40
**Athletics**	7.61	**24.49**	37.06	26.82	**38.14**	47.68	40.18	**47.62**	55.06	47.56	**57.11**	68.42	58.18	**70.75**	87.64
**Basketball**	15.76	**34.38**	49.40	44.34	**56.96**	68.10	64.20	**72.65**	81.09	77.20	**88.34**	100.96	95.90	**110.91**	129.53
**Fencing**	0.00	**29.49**	54.55	25.67	**50.06**	69.48	48.81	**64.36**	79.86	59.19	**78.66**	103.00	74.12	**99.23**	136.29
**Gymnastics**	0.00	**25.93**	44.99	28.09	**45.51**	59.98	47.83	**59.12**	70.40	58.25	**72.72**	90.14	73.24	**92.30**	118.53
**Handball**	1.54	**40.30**	68.27	42.61	**68.36**	89.66	71.16	**87.87**	104.53	86.03	**107.38**	133.08	107.42	**135.45**	174.15
**Hockey Rink**	13.78	**35.45**	52.93	47.38	**62.04**	74.99	70.73	**80.53**	90.32	86.06	**99.01**	113.67	108.12	**125.60**	147.27
**Korfball**	0.00	**31.84**	54.49	27.59	**49.89**	67.41	48.48	**62.43**	76.39	57.46	**74.98**	97.28	70.38	**93.03**	127.32
**Modern Pentathlon**	0.00	**26.60**	47.24	25.03	**44.69**	60.60	44.66	**57.27**	69.89	53.95	**69.85**	89.52	67.31	**87.95**	117.76
**Motorsport**	NA	**NA**	NA	NA	**NA**	NA	NA	**NA**	NA	NA	**NA**	NA	NA	**NA**	NA
**Other combat sports**	8.25	**29.57**	45.81	34.91	**49.22**	61.43	53.44	**62.89**	72.29	64.30	**76.55**	90.82	79.92	**96.21**	117.48
**Rowing**	7.66	**28.92**	45.04	33.52	**47.77**	59.92	51.49	**60.88**	70.27	61.83	**73.98**	88.24	76.71	**92.83**	114.09
**Rugby**	0.00	**18.39**	49.55	46.93	**72.38**	94.98	93.24	**109.90**	126.55	124.82	**147.42**	172.87	170.25	**201.40**	239.49
**Sailing**	2.03	**33.14**	57.53	45.37	**66.20**	84.32	75.49	**89.19**	102.94	94.11	**112.17**	133.07	120.90	**145.24**	176.41
**Soccer**	15.13	**35.48**	50.17	35.59	**49.24**	60.59	49.82	**58.81**	67.84	57.06	**68.38**	82.06	67.49	**82.14**	102.52
**Surf**	NA	**NA**	NA	NA	**NA**	NA	NA	**NA**	NA	NA	**NA**	NA	NA	**NA**	NA
**Swimming**	7.37	**24.27**	37.72	32.15	**43.54**	53.52	49.37	**56.93**	64.50	60.35	**70.33**	81.72	76.15	**89.60**	106.50
**Tennis**	14.61	**38.53**	56.02	39.79	**55.83**	69.25	57.30	**67.85**	78.45	66.50	**79.88**	95.96	79.73	**97.17**	121.14
**Triathlon**	16.03	**28.82**	38.82	32.78	**41.47**	49.02	44.42	**50.26**	56.10	51.51	**59.05**	67.75	61.70	**71.70**	84.49
**Volleyball**	9.60	**37.69**	57.84	38.01	**56.75**	72.24	57.76	**70.00**	82.25	67.77	**83.26**	102.00	82.17	**102.32**	130.41
**Wrestling and Judo**	6.59	**21.99**	34.72	33.74	**44.17**	53.50	52.62	**59.58**	66.55	65.67	**75.00**	85.42	84.45	**97.18**	112.58

NA: data not presented for n < 8.

Table 5 – Sum of appendicular skinfolds [triceps + biceps + thigh + medial calf (mm)] percentiles by sport and sex

Sport	0.05			0.25			Median			0.75			0.95		
	Low	Estimate	High	Low	Estimate	High	Low	Estimate	High	Low	Estimate	High	Low	Estimate	High
**Females**															
**Archery and Shooting**	NA	**NA**	NA	NA	**NA**	NA	NA	**NA**	NA	NA	**NA**	NA	NA	**NA**	NA
**Athletics**	0.00	**10.62**	23.06	15.14	**25.93**	35.42	29.13	**36.57**	44.01	37.72	**47.21**	58.00	50.08	**62.52**	78.13
**Basketball**	18.69	**34.51**	47.56	42.39	**53.44**	63.36	58.86	**66.60**	74.35	69.84	**79.76**	90.82	85.64	**98.70**	114.51
**Fencing**	NA	**NA**	NA	NA	**NA**	NA	NA	**NA**	NA	NA	**NA**	NA	NA	**NA**	NA
**Gymnastics**	10.29	**28.44**	42.82	30.90	**43.74**	55.01	45.23	**54.38**	63.49	53.71	**65.02**	77.82	65.91	**80.32**	98.44
**Handball**	NA	**NA**	NA	NA	**NA**	NA	NA	**NA**	NA	NA	**NA**	NA	NA	**NA**	NA
**Hockey Rink**	NA	**NA**	NA	NA	**NA**	NA	NA	**NA**	NA	NA	**NA**	NA	NA	**NA**	NA
**Korfball**	10.52	**34.51**	53.26	32.37	**49.76**	65.00	47.55	**60.36**	73.17	55.71	**70.96**	88.35	67.45	**86.21**	110.19
**Modern Pentathlon**	0.84	**24.51**	42.91	21.80	**38.94**	53.93	36.37	**48.98**	61.59	44.02	**59.01**	76.16	55.04	**73.44**	97.11
**Motorsport**	NA	**NA**	NA	NA	**NA**	NA	NA	**NA**	NA	NA	**NA**	NA	NA	**NA**	NA
**Other combat sports**	2.97	**27.67**	46.92	27.06	**44.71**	60.12	43.80	**56.55**	69.30	52.97	**68.39**	86.04	66.18	**85.43**	110.13
**Rowing**	8.44	**32.17**	50.71	29.31	**46.59**	61.75	43.81	**56.62**	69.43	51.49	**66.65**	83.93	62.54	**81.07**	104.80
**Rugby**	NA	**NA**	NA	NA	**NA**	NA	NA	**NA**	NA	NA	**NA**	NA	NA	**NA**	NA
**Sailing**	NA	**NA**	NA	NA	**NA**	NA	NA	**NA**	NA	NA	**NA**	NA	NA	**NA**	NA
**Soccer**	15.08	**31.99**	45.58	35.61	**47.56**	58.15	49.88	**58.38**	66.88	58.61	**69.20**	81.15	71.17	**84.76**	101.68
**Surf**	NA	**NA**	NA	NA	**NA**	NA	NA	**NA**	NA	NA	**NA**	NA	NA	**NA**	NA
**Swimming**	11.66	**26.17**	37.87	30.38	**40.57**	49.59	43.39	**50.58**	57.74	51.54	**60.59**	70.76	63.27	**74.99**	89.48
**Tennis**	29.68	**51.05**	67.95	49.43	**65.05**	78.84	63.15	**74.78**	86.41	70.72	**84.51**	100.13	81.61	**98.51**	119.88
**Triathlon**	0.00	**18.80**	39.48	14.95	**34.38**	51.26	30.95	**45.21**	59.45	39.14	**56.05**	75.45	50.92	**71.63**	98.46
**Volleyball**	7.90	**31.17**	49.49	33.39	**49.86**	64.28	51.11	**62.85**	74.55	61.38	**75.84**	92.27	76.17	**94.54**	117.76
**Wrestling and Judo**	11.62	**33.74**	51.38	38.13	**53.69**	67.42	56.56	**67.56**	78.57	67.70	**81.43**	96.99	83.74	**101.38**	123.50
**Males**															
**Archery and Shooting**	1.42	**22.75**	36.30	18.70	**32.50**	43.10	30.71	**39.27**	47.83	35.44	**46.05**	59.84	42.24	**55.79**	77.12
**Athletics**	4.53	**11.28**	16.29	12.14	**16.67**	20.48	17.43	**20.42**	23.39	20.35	**24.17**	28.68	24.54	**29.56**	36.29
**Basketball**	9.05	**17.23**	23.82	21.54	**27.09**	31.98	30.22	**33.94**	37.66	35.90	**40.79**	46.34	44.06	**50.65**	58.83
**Fencing**	0.00	**11.13**	23.27	9.29	**21.21**	30.47	20.92	**28.21**	35.48	25.92	**35.21**	47.11	33.13	**45.28**	63.84
**Gymnastics**	1.51	**12.99**	21.30	13.84	**21.46**	27.77	22.42	**27.34**	32.27	26.92	**33.23**	40.84	33.39	**41.70**	53.17
**Handball**	1.51	**17.44**	28.91	18.29	**28.86**	37.58	29.96	**36.79**	43.61	35.99	**44.72**	55.28	44.67	**56.13**	72.07
**Hockey Rink**	2.00	**13.26**	22.31	19.49	**27.05**	33.72	31.65	**36.64**	41.65	39.57	**46.22**	53.81	50.98	**60.01**	71.30
**Korfball**	0.00	**14.31**	23.79	12.39	**21.80**	29.14	21.17	**27.02**	32.85	24.88	**32.23**	41.63	30.22	**39.73**	54.25
**Modern Pentathlon**	0.00	**9.60**	20.51	8.74	**19.18**	27.52	19.27	**25.83**	32.40	24.14	**32.49**	42.92	31.15	**42.06**	58.07
**Motorsport**	NA	**NA**	NA	NA	**NA**	NA	NA	**NA**	NA	NA	**NA**	NA	NA	**NA**	NA
**Other combat sports**	0.00	**9.13**	19.44	12.65	**21.70**	29.37	24.61	**30.44**	36.27	31.51	**39.18**	48.23	41.44	**51.76**	65.44
**Rowing**	1.81	**11.95**	19.61	14.12	**20.88**	26.64	22.68	**27.10**	31.53	27.57	**33.31**	40.09	34.61	**42.25**	52.40
**Rugby**	0.00	**2.68**	17.55	16.34	**28.47**	39.20	38.52	**46.39**	54.25	53.58	**64.31**	76.44	75.23	**90.10**	108.35
**Sailing**	0.00	**9.32**	22.39	15.96	**27.15**	36.77	32.31	**39.54**	46.77	42.30	**51.93**	63.11	56.68	**69.76**	86.62
**Soccer**	3.73	**13.70**	20.80	13.74	**20.37**	25.83	20.70	**25.01**	29.32	24.20	**29.65**	36.28	29.23	**36.33**	46.30
**Surf**	NA	**NA**	NA	NA	**NA**	NA	NA	**NA**	NA	NA	**NA**	NA	NA	**NA**	NA
**Swimming**	5.70	**12.80**	18.45	16.03	**20.84**	25.04	23.21	**26.42**	29.62	27.79	**32.01**	36.80	34.39	**40.05**	47.14
**Tennis**	9.13	**19.09**	26.33	19.50	**26.18**	31.75	26.71	**31.11**	35.52	30.47	**36.04**	42.72	35.89	**43.14**	53.09
**Triathlon**	7.37	**13.30**	17.92	15.11	**19.13**	22.62	20.49	**23.19**	25.88	23.76	**27.24**	31.27	28.45	**33.08**	39.01
**Volleyball**	0.00	**12.97**	22.29	13.11	**21.83**	28.94	22.38	**27.98**	33.57	27.01	**34.14**	42.84	33.66	**43.00**	56.19
**Wrestling and Judo**	4.22	**10.78**	16.19	15.74	**20.19**	24.16	23.75	**26.73**	29.70	29.29	**33.27**	37.70	37.25	**42.68**	49.22

NA: data not presented for n < 8.

Table 6 – Sum of arm skinfolds [triceps + biceps (mm)] percentiles by sport and sex

Sport	0.05			0.25			Median			0.75			0.95		
	Low	Estimate	High	Low	Estimate	High	Low	Estimate	High	Low	Estimate	High	Low	Estimate	High
**Females**															
Archery and Shooting	NA	**NA**	NA	NA	**NA**	NA	NA	**NA**	NA	NA	**NA**	NA	NA	**NA**	NA
Athletics	0,00	**4,20**	8,91	6,05	**10,11**	13,73	11,38	**14,22**	17,08	14,73	**18,33**	22,40	19,55	**24,25**	30,06
Basketball	4,79	**10,88**	15,95	14,11	**18,39**	22,24	20,60	**23,61**	26,62	24,97	**28,83**	33,10	31,27	**36,34**	42,43
Fencing	NA	**NA**	NA	NA	**NA**	NA	NA	**NA**	NA	NA	**NA**	NA	NA	**NA**	NA
Gymnastics	0,12	**7,95**	14,25	9,32	**14,89**	19,82	15,72	**19,71**	23,70	19,59	**24,54**	30,10	25,17	**31,48**	39,29
Handball	NA	**NA**	NA	NA	**NA**	NA	NA	**NA**	NA	NA	**NA**	NA	NA	**NA**	NA
Hockey Rink	NA	**NA**	NA	NA	**NA**	NA	NA	**NA**	NA	NA	**NA**	NA	NA	**NA**	NA
Korfball	3,67	**13,40**	21,24	12,81	**19,99**	26,39	19,15	**24,56**	29,97	22,74	**29,14**	36,32	27,89	**35,72**	45,45
Modern Pentathlon	0,00	**9,38**	17,19	8,52	**15,72**	22,11	14,71	**20,12**	25,53	18,13	**24,53**	31,72	23,06	**30,86**	40,62
Motorsport	NA	**NA**	NA	NA	**NA**	NA	NA	**NA**	NA	NA	**NA**	NA	NA	**NA**	NA
Other combat sports	0,17	**10,34**	18,47	10,38	**17,76**	24,31	17,48	**22,93**	28,37	21,54	**28,09**	35,47	27,38	**35,52**	45,68
Rowing	1,93	**11,79**	19,70	10,88	**18,18**	24,68	17,10	**22,63**	28,14	20,56	**27,07**	34,36	25,53	**33,46**	43,30
Rugby	NA	**NA**	NA	NA	**NA**	NA	NA	**NA**	NA	NA	**NA**	NA	NA	**NA**	NA
Sailing	NA	**NA**	NA	NA	**NA**	NA	NA	**NA**	NA	NA	**NA**	NA	NA	**NA**	NA
Soccer	3,33	**9,57**	14,65	11,13	**15,57**	19,53	16,55	**19,74**	22,93	19,94	**23,91**	28,35	24,82	**29,91**	36,15
Surf	NA	**NA**	NA	NA	**NA**	NA	NA	**NA**	NA	NA	**NA**	NA	NA	**NA**	NA
Swimming	1,79	**7,86**	12,80	9,84	**14,12**	17,93	15,44	**18,47**	21,50	19,00	**22,82**	27,10	24,13	**29,08**	35,15
Tennis	9,46	**18,82**	26,39	18,42	**25,36**	31,55	24,65	**29,90**	35,13	28,24	**34,45**	41,36	33,40	**40,98**	50,33
Triathlon	0,00	**6,20**	15,30	4,88	**13,31**	20,76	11,94	**18,25**	24,56	15,73	**23,20**	31,61	21,20	**30,31**	41,76
Volleyball	2,75	**10,77**	17,24	11,78	**17,54**	22,66	18,05	**22,24**	26,43	21,82	**26,94**	32,70	27,24	**33,71**	41,73
Wrestling and Judo	1,60	**10,28**	17,32	12,32	**18,47**	23,95	19,76	**24,17**	28,57	24,38	**29,86**	36,02	31,02	**38,05**	46,73
**Males**															
Archery and Shooting	2.67	**10.91**	16.19	9.30	**14.66**	18.81	13.90	**17.27**	20.63	15.73	**19.87**	25.24	18.35	**23.62**	31.87
Athletics	1.03	**4.44**	6.95	4.89	**7.16**	9.06	7.57	**9.05**	10.53	9.04	**10.94**	13.21	11.15	**13.67**	17.07
Basketball	3.36	**6.86**	9.69	8.71	**11.09**	13.19	12.43	**14.03**	15.62	14.86	**16.96**	19.34	18.36	**21.19**	24.70
Fencing	0.00	**5.58**	10.40	4.83	**9.55**	13.24	9.40	**12.30**	15.22	11.37	**15.06**	19.78	14.21	**19.03**	26.36
Gymnastics	0.39	**5.49**	9.18	5.87	**9.25**	12.05	9.68	**11.87**	14.05	11.68	**14.48**	17.86	14.55	**18.24**	23.35
Handball	0.00	**6.25**	11.58	6.66	**11.58**	15.62	12.14	**15.29**	18.44	14.95	**19.00**	23.91	19.00	**24.34**	31.79
Hockey Rink	1.74	**5.92**	9.28	8.21	**11.03**	13.51	12.71	**14.58**	16.44	15.64	**18.12**	20.94	19.87	**23.23**	27.41
Korfball	0.00	**6.28**	10.79	5.37	**9.84**	13.32	9.54	**12.31**	15.07	11.30	**14.78**	19.25	13.82	**18.33**	25.25
Modern Pentathlon	0.00	**3.62**	9.00	3.19	**8.35**	12.46	8.43	**11.65**	14.86	10.83	**14.94**	20.10	14.29	**19.67**	27.63
Motorsport	NA	**NA**	NA	NA	**NA**	NA	NA	**NA**	NA	NA	**NA**	NA	NA	**NA**	NA
Other combat sports	0.08	**4.74**	8.28	5.92	**9.02**	11.67	9.97	**12.00**	14.03	12.33	**14.97**	18.08	15.71	**19.25**	23.92
Rowing	1.36	**5.43**	8.50	6.29	**9.00**	11.32	9.71	**11.49**	13.28	11.67	**13.97**	16.70	14.48	**17.55**	21.62
Rugby	0.00	**1.25**	7.23	6.74	**11.62**	15.94	15.66	**18.83**	21.99	21.71	**26.03**	30.91	30.42	**36.40**	43.75
Sailing	0.67	**6.24**	10.59	8.39	**12.12**	15.34	13.76	**16.20**	18.65	17.06	**20.29**	24.02	21.82	**26.17**	31.74
Soccer	0.93	**5.52**	8.78	5.54	**8.59**	11.08	8.75	**10.72**	12.69	10.36	**12.85**	15.90	12.67	**15.92**	20.52
Surf	NA	**NA**	NA	NA	**NA**	NA	NA	**NA**	NA	NA	**NA**	NA	NA	**NA**	NA
Swimming	2.14	**4.96**	7.20	6.24	**8.14**	9.81	9.09	**10.36**	11.63	10.91	**12.57**	14.48	13.52	**15.76**	18.58
Tennis	3.65	**8.12**	11.38	8.30	**11.31**	13.81	11.54	**13.52**	15.50	13.23	**15.73**	18.73	15.66	**18.92**	23.39
Triathlon	2.34	**5.06**	7.18	5.91	**7.74**	9.34	8.39	**9.61**	10.83	9.89	**11.47**	13.31	12.04	**14.15**	16.88
Volleyball	1.46	**6.14**	9.48	6.16	**9.28**	11.83	9.44	**11.45**	13.47	11.07	**13.63**	16.74	13.43	**16.76**	21.45
Wrestling and Judo	2.10	**4.61**	6.68	6.50	**8.20**	9.72	9.55	**10.69**	11.84	11.66	**13.19**	14.89	14.70	**16.78**	19.29

NA: data not presented for n < 8.

Table 7 – Sum of leg skinfolds [thigh + medial calf (mm)] percentiles by sport and sex

Sport		0.05			0.25			Median			0.75			0.95	
	Low	Estimate	High	Low	Estimate	High	Low	Estimate	High	Low	Estimate	High	Low	Estimate	High
**Females**															
Archery and Shooting	NA	**NA**	NA	NA	**NA**	NA	NA	**NA**	NA	NA	**NA**	NA	NA	**NA**	NA
Athletics	0.00	**4.46**	13.24	7.31	**15.00**	21.61	17.21	**22.32**	27.43	23.03	**29.64**	37.32	31.39	**40.17**	51.56
Basketball	11.18	**21.95**	30.70	26.92	**34.36**	40.96	37.86	**42.97**	48.09	44.99	**51.59**	59.03	55.25	**63.99**	74.77
Fencing	NA	**NA**	NA	NA	**NA**	NA	NA	**NA**	NA	NA	**NA**	NA	NA	**NA**	NA
Gymnastics	4.59	**17.45**	27.30	18.71	**27.58**	35.22	28.52	**34.63**	40.73	34.03	**41.67**	50.54	41.95	**51.80**	64.67
Handball	NA	**NA**	NA	NA	**NA**	NA	NA	**NA**	NA	NA	**NA**	NA	NA	**NA**	NA
Hockey Rink	NA	**NA**	NA	NA	**NA**	NA	NA	**NA**	NA	NA	**NA**	NA	NA	**NA**	NA
Korfball	3.00	**19.59**	31.81	17.49	**29.11**	38.91	27.57	**35.72**	43.85	32.51	**42.34**	53.93	39.62	**51.86**	68.43
Modern Pentathlon	0.00	**13.06**	25.40	10.55	**22.33**	32.24	20.58	**28.78**	36.99	25.33	**35.22**	47.02	32.17	**44.49**	61.44
Motorsport	NA	**NA**	NA	NA	**NA**	NA	NA	**NA**	NA	NA	**NA**	NA	NA	**NA**	NA
Other combat sports	0.00	**16.05**	28.23	14.99	**26.38**	36.03	25.68	**33.56**	41.44	31.10	**40.74**	52.13	38.89	**51.07**	67.51
Rowing	2.94	**19.02**	30.86	16.50	**27.81**	37.37	25.93	**33.92**	41.90	30.46	**40.02**	51.33	36.97	**48.81**	64.89
Rugby	NA	**NA**	NA	NA	**NA**	NA	NA	**NA**	NA	NA	**NA**	NA	NA	**NA**	NA
Sailing	NA	**NA**	NA	NA	**NA**	NA	NA	**NA**	NA	NA	**NA**	NA	NA	**NA**	NA
Soccer	7.33	**19.99**	29.86	22.24	**30.98**	38.58	32.60	**38.61**	44.64	38.66	**46.24**	55.01	47.38	**57.23**	69.92
Surf	NA	**NA**	NA	NA	**NA**	NA	NA	**NA**	NA	NA	**NA**	NA	NA	**NA**	NA
Swimming	6.91	**16.62**	24.27	19.05	**25.75**	31.60	27.48	**32.09**	36.70	32.58	**38.43**	45.13	39.91	**47.55**	57.26
Tennis	13.40	**28.99**	40.70	27.32	**38.33**	47.75	37.00	**44.81**	52.65	41.90	**51.30**	62.33	48.95	**60.63**	76.26
Triathlon	0.00	**10.73**	23.98	7.46	**20.25**	30.93	17.98	**26.87**	35.76	22.81	**33.49**	46.28	29.76	**43.02**	61.41
Volleyball	0.00	**17.22**	31.15	18.33	**30.99**	41.80	31.93	**40.57**	49.20	39.34	**50.14**	62.80	49.99	**63.92**	82.37
Wrestling and Judo	1.20	**18.48**	31.78	21.33	**33.16**	43.36	35.32	**43.36**	51.41	43.36	**53.56**	65.40	54.94	**68.24**	85.52
**Males**															
Archery and Shooting	0.00	**12.74**	21.42	10.13	**19.00**	25.77	17.90	**23.35**	28.80	20.92	**27.70**	36.57	25.27	**33.95**	47.75
Athletics	0.00	**4.81**	8.66	5.54	**9.01**	11.91	9.69	**11.93**	14.17	11.95	**14.85**	18.32	15.20	**19.05**	24.29
Basketball	3.77	**9.16**	13.50	12.03	**15.67**	18.88	17.77	**20.19**	22.62	21.51	**24.72**	28.36	26.89	**31.23**	36.62
Fencing	0.00	**4.74**	13.38	3.45	**11.94**	18.50	11.84	**16.94**	22.05	15.39	**21.94**	30.44	20.51	**29.14**	42.50
Gymnastics	0.00	**6.70**	12.13	7.29	**12.26**	16.37	12.92	**16.12**	19.31	15.87	**19.98**	24.95	20.10	**25.54**	33.06
Handball	1.77	**11.00**	17.64	11.47	**17.59**	22.65	18.20	**22.17**	26.13	21.68	**26.75**	32.87	26.70	**33.33**	42.56
Hockey Rink	0.00	**6.51**	12.61	10.75	**15.85**	20.32	19.00	**22.34**	25.68	24.35	**28.83**	33.92	32.06	**38.16**	45.79
Korfball	0.00	**6.48**	13.40	5.11	**12.00**	17.30	11.64	**15.83**	20.01	14.35	**19.66**	26.54	18.25	**25.18**	35.94
Modern Pentathlon	0.00	**5.07**	11.79	4.53	**10.97**	16.10	11.06	**15.08**	19.11	14.06	**19.19**	25.63	18.38	**25.10**	35.02
Motorsport	NA	**NA**	NA	NA	**NA**	NA	NA	**NA**	NA	NA	**NA**	NA	NA	**NA**	NA
Other combat sports	0.00	**0.50**	9.32	3.63	**11.35**	17.83	14.04	**18.89**	23.74	19.95	**26.43**	34.16	28.46	**37.28**	49.14
Rowing	0.00	**5.46**	10.79	7.02	**11.73**	15.71	13.05	**16.09**	19.13	16.47	**20.45**	25.16	21.39	**26.72**	33.84
Rugby	0.00	**0.99**	10.10	9.37	**16.79**	23.36	22.97	**27.78**	32.58	32.19	**38.76**	46.18	45.45	**54.56**	65.76
Sailing	0.00	**1.29**	10.93	6.28	**14.51**	21.54	18.46	**23.69**	28.92	25.83	**32.87**	41.09	36.44	**46.08**	58.61
Soccer	0.00	**6.74**	11.89	6.82	**11.63**	15.57	11.96	**15.04**	18.12	14.51	**18.44**	23.26	18.18	**23.34**	30.66
Surf	NA	**NA**	NA	NA	**NA**	NA	NA	**NA**	NA	NA	**NA**	NA	NA	**NA**	NA
Swimming	1.89	**6.85**	10.79	9.13	**12.47**	15.39	14.17	**16.38**	18.59	17.37	**20.29**	23.62	21.97	**25.91**	30.87
Tennis	2.97	**9.89**	14.89	10.22	**14.83**	18.65	15.27	**18.27**	21.26	17.88	**21.70**	26.31	21.64	**26.64**	33.56
Triathlon	2.33	**6.69**	10.06	8.06	**10.99**	13.51	12.04	**13.98**	15.91	14.44	**16.96**	19.89	17.89	**21.26**	25.62
Volleyball	0.00	**6.03**	12.96	6.19	**12.67**	17.92	13.18	**17.28**	21.37	16.63	**21.89**	28.36	21.59	**28.52**	38.42
Wrestling and Judo	0.95	**5.40**	9.07	8.79	**11.80**	14.48	14.25	**16.24**	18.24	18.01	**20.69**	23.69	23.42	**27.09**	31.54

NA: data not presented for n < 8.

Table 8 – Sum of trunk skinfolds [subscapular + suprailiac + abdominal (mm)] percentiles by sport and sex

Sport	0.05			0.25			Median			0.75			0.95		
	Low	Estimate	High	Low	Estimate	High	Low	Estimate	High	Low	Estimate	High	Low	Estimate	High
**Females**															
Archery and Shooting	0.00	**17.37**	42.54	11.08	**36.32**	55.81	33.96	**49.49**	65.03	43.18	**62.67**	87.91	56.44	**81.62**	120.84
Athletics	0.00	**9.11**	18.57	11.13	**19.59**	26.71	21.39	**26.88**	32.36	27.04	**34.16**	42.62	35.18	**44.64**	57.38
Basketball	1.55	**13.59**	23.27	20.20	**28.31**	35.46	33.16	**38.54**	43.92	41.63	**48.77**	56.89	53.81	**63.49**	75.54
Fencing	0.00	**14.70**	29.43	12.80	**27.00**	38.40	26.46	**35.54**	44.63	32.69	**44.09**	58.29	41.65	**56.38**	77.94
Gymnastics	0.00	**9.72**	21.95	11.39	**22.51**	31.74	24.25	**31.40**	38.54	31.06	**40.29**	51.40	40.85	**53.07**	69.90
Handball	0.00	**16.77**	36.37	18.86	**36.78**	51.62	39.16	**50.69**	62.22	49.76	**64.60**	82.53	65.01	**84.61**	111.74
Hockey Rink	4.93	**17.46**	27.58	24.49	**32.96**	40.43	38.09	**43.73**	49.37	47.02	**54.50**	62.96	59.88	**70.00**	82.52
Korfball	0.00	**13.63**	28.92	11.19	**26.10**	37.86	25.48	**34.76**	44.06	31.69	**43.42**	58.35	40.62	**55.89**	78.91
Modern Pentathlon	0.00	**11.90**	24.46	11.20	**23.12**	32.76	23.29	**30.92**	38.53	29.06	**38.72**	50.62	37.37	**49.94**	68.00
Motorsport	NA	**NA**	NA	NA	**NA**	NA	NA	**NA**	NA	NA	**NA**	NA	NA	**NA**	NA
Other combat sports	1.85	**13.59**	22.58	16.69	**24.56**	31.30	27.00	**32.18**	37.36	33.07	**39.80**	47.67	41.79	**50.77**	62.51
Rowing	0.73	**13.72**	23.57	16.70	**25.39**	32.79	27.80	**33.50**	39.20	34.21	**41.61**	50.30	43.42	**53.28**	66.27
Rugby	0.00	**11.45**	29.06	27.67	**42.09**	54.88	53.90	**63.38**	72.82	71.85	**84.68**	99.06	97.67	**115.32**	136.81
Sailing	0.00	**15.08**	29.88	22.71	**35.35**	46.31	41.16	**49.44**	57.73	52.58	**63.53**	76.18	69.01	**83.81**	102.72
Soccer	6.18	**18.71**	27.78	18.94	**27.35**	34.34	27.81	**33.36**	38.90	32.38	**39.37**	47.78	38.94	**48.01**	60.54
Surf	NA	**NA**	NA	NA	**NA**	NA	NA	**NA**	NA	NA	**NA**	NA	NA	**NA**	NA
Swimming	0.00	**8.99**	17.67	14.25	**21.58**	27.98	25.51	**30.33**	35.15	32.67	**39.08**	46.41	42.98	**51.66**	62.61
Tennis	0.15	**16.24**	27.96	17.38	**28.10**	37.03	29.36	**36.35**	43.33	35.67	**44.60**	55.31	44.74	**56.46**	72.54
Triathlon	4.43	**12.73**	19.20	15.45	**21.06**	25.91	23.11	**26.84**	30.57	27.77	**32.62**	38.23	34.48	**40.95**	49.25
Volleyball	3.08	**20.81**	33.58	21.23	**33.07**	42.87	33.84	**41.59**	49.33	40.30	**50.11**	61.95	49.59	**62.37**	80.10
Wrestling and Judo	0.00	**7.94**	16.25	15.78	**22.56**	28.62	28.25	**32.73**	37.21	36.84	**42.90**	49.68	49.21	**57.52**	67.62
**Males**															
Archery and Shooting	NA	**NA**	NA	NA	**NA**	NA	NA	**NA**	NA	NA	**NA**	NA	NA	**NA**	NA
Athletics	0.00	**12.55**	23.74	17.03	**26.68**	35.31	29.65	**36.50**	43.34	37.69	**46.32**	55.97	49.26	**60.45**	74.14
Basketball	14.93	**29.39**	41.49	37.19	**47.39**	56.62	52.65	**59.90**	67.14	63.17	**72.40**	82.61	78.30	**90.40**	104.86
Fencing	NA	**NA**	NA	NA	**NA**	NA	NA	**NA**	NA	NA	**NA**	NA	NA	**NA**	NA
Gymnastics	0.00	**13.98**	26.89	16.98	**28.35**	38.48	30.16	**38.34**	46.53	38.21	**48.33**	59.71	49.79	**62.70**	78.66
Handball	NA	**NA**	NA	NA	**NA**	NA	NA	**NA**	NA	NA	**NA**	NA	NA	**NA**	NA
Hockey Rink	NA	**NA**	NA	NA	**NA**	NA	NA	**NA**	NA	NA	**NA**	NA	NA	**NA**	NA
Korfball	3.55	**26.91**	45.84	25.76	**43.05**	58.53	41.20	**54.28**	67.35	50.02	**65.50**	82.79	62.71	**81.64**	105.00
Modern Pentathlon	0.00	**12.56**	33.01	10.78	**29.50**	46.17	27.27	**41.28**	55.32	36.42	**53.06**	71.81	49.58	**70.01**	95.54
Motorsport	NA	**NA**	NA	NA	**NA**	NA	NA	**NA**	NA	NA	**NA**	NA	NA	**NA**	NA
Other combat sports	0.00	**22.27**	40.93	22.61	**39.50**	54.54	38.94	**51.47**	64.00	48.40	**63.44**	80.33	62.01	**80.66**	103.81
Rowing	1.09	**23.73**	42.10	21.91	**38.74**	53.84	36.38	**49.18**	62.00	44.54	**59.61**	76.47	56.28	**74.62**	97.29
Rugby	NA	**NA**	NA	NA	**NA**	NA	NA	**NA**	NA	NA	**NA**	NA	NA	**NA**	NA
Sailing	NA	**NA**	NA	NA	**NA**	NA	NA	**NA**	NA	NA	**NA**	NA	NA	**NA**	NA
Soccer	5.80	**21.55**	34.45	25.68	**36.92**	46.99	39.50	**47.60**	55.71	48.22	**58.29**	69.53	60.76	**73.66**	89.41
Surf	NA	**NA**	NA	NA	**NA**	NA	NA	**NA**	NA	NA	**NA**	NA	NA	**NA**	NA
Swimming	3.68	**17.99**	29.73	22.85	**32.98**	42.05	36.18	**43.39**	50.61	44.74	**53.81**	63.93	57.05	**68.79**	83.10
Tennis	14.19	**36.71**	55.03	36.11	**52.79**	67.76	51.34	**63.98**	76.61	60.19	**75.16**	91.84	72.93	**91.24**	113.76
Triathlon	0.00	**12.22**	36.51	9.04	**31.43**	51.35	27.91	**44.78**	61.65	38.22	**58.14**	80.53	53.05	**77.35**	107.68
Volleyball	10.20	**28.46**	43.35	30.89	**44.11**	55.96	45.26	**54.99**	64.72	54.03	**65.87**	79.10	66.63	**81.52**	99.78
Wrestling and Judo	4.89	**24.05**	39.71	28.74	**42.40**	54.64	45.32	**55.15**	65.01	55.70	**67.90**	81.60	70.63	**86.25**	105.45

NA: data not presented for n < 8.

Table 9 – Arm circumference (cm) percentiles by sport and sex

Sport	0.05			0.25			Median			0.75			0.95		
	Low	Estimate	High	Low	Estimate	High	Low	Estimate	High	Low	Estimate	High	Low	Estimate	High
**Females**															
**Archery and Shooting**	NA	**NA**	NA	NA	**NA**	NA	NA	**NA**	NA	NA	**NA**	NA	NA	**NA**	NA
**Athletics**	20.69	**22.24**	23.71	23.07	**24.38**	25.64	24.73	**25.86**	26.99	26.08	**27.34**	28.65	28.01	**29.48**	31.03
**Basketball**	22.11	**23.47**	24.75	24.52	**25.64**	26.73	26.20	**27.15**	28.10	27.57	**28.66**	29.78	29.55	**30.83**	32.19
**Fencing**	NA	**NA**	NA	NA	**NA**	NA	NA	**NA**	NA	NA	**NA**	NA	NA	**NA**	NA
**Gymnastics**	19.91	**21.69**	23.37	22.36	**23.87**	25.34	24.06	**25.38**	26.71	25.43	**26.90**	28.41	27.40	**29.08**	30.86
**Handball**	NA	**NA**	NA	NA	**NA**	NA	NA	**NA**	NA	NA	**NA**	NA	NA	**NA**	NA
**Hockey Rink**	NA	**NA**	NA	NA	**NA**	NA	NA	**NA**	NA	NA	**NA**	NA	NA	**NA**	NA
**Korfball**	20.79	**23.06**	25.23	23.22	**25.21**	27.17	24.90	**26.71**	28.52	26.25	**28.21**	30.20	28.19	**30.36**	32.63
**Modern Pentathlon**	20.64	**23.05**	25.36	23.12	**25.25**	27.34	24.84	**26.78**	28.71	26.22	**28.31**	30.44	28.19	**30.50**	32.91
**Motorsport**	NA	**NA**	NA	NA	**NA**	NA	NA	**NA**	NA	NA	**NA**	NA	NA	**NA**	NA
**Other combat sports**	21.81	**23.97**	26.02	24.29	**26.17**	28.01	26.02	**27.71**	29.40	27.40	**29.24**	31.12	29.39	**31.45**	33.61
**Rowing**	21.36	**23.73**	25.98	23.78	**25.87**	27.91	25.46	**27.37**	29.25	26.80	**28.86**	30.94	28.74	**31.01**	33.35
**Rugby**	NA	**NA**	NA	NA	**NA**	NA	NA	**NA**	NA	NA	**NA**	NA	NA	**NA**	NA
**Sailing**	NA	**NA**	NA	NA	**NA**	NA	NA	**NA**	NA	NA	**NA**	NA	NA	**NA**	NA
**Soccer**	20.86	**22.53**	24.09	23.29	**24.71**	26.06	24.99	**26.22**	27.44	26.36	**27.74**	29.13	28.34	**29.92**	31.57
**Surf**	NA	**NA**	NA	NA	**NA**	NA	NA	**NA**	NA	NA	**NA**	NA	NA	**NA**	NA
**Swimming**	22.75	**24.29**	25.76	25.14	**26.44**	27.70	26.80	**27.93**	29.05	28.15	**29.42**	30.71	30.09	**31.56**	33.11
**Tennis**	22.67	**24.90**	27.03	25.14	**27.09**	29.01	26.85	**28.62**	30.38	28.23	**30.14**	32.10	30.21	**32.34**	34.57
**Triathlon**	20.77	**23.31**	25.78	23.27	**25.54**	27.78	25.02	**27.08**	29.17	26.40	**28.63**	30.91	28.40	**30.85**	33.41
**Volleyball**	22.42	**24.33**	26.14	24.92	**26.56**	28.17	26.67	**28.12**	29.57	28.07	**29.67**	31.31	30.09	**31.91**	33.82
**Wrestling and Judo**	22.38	**24.10**	25.75	24.87	**26.32**	27.76	26.60	**27.87**	29.16	28.00	**29.42**	30.89	30.01	**31.65**	33.38
**Males**															
**Archery and Shooting**	18.49	**23.96**	28.12	23.01	**26.97**	30.39	26.15	**29.06**	31.97	27.73	**31.15**	35.11	30.00	**34.16**	39.63
**Athletics**	22.28	**24.89**	26.98	25.18	**27.06**	28.73	27.19	**28.57**	29.95	28.40	**30.08**	31.96	30.15	**32.25**	34.86
**Basketball**	23.28	**25.43**	27.25	26.49	**28.03**	29.44	28.72	**29.84**	30.97	30.24	**31.65**	33.20	32.44	**34.25**	36.41
**Fencing**	19.84	**23.47**	26.28	23.09	**25.72**	28.01	25.34	**27.28**	29.21	26.54	**28.84**	31.47	28.27	**31.08**	34.71
**Gymnastics**	20.86	**24.33**	27.09	24.59	**27.08**	29.27	27.18	**28.99**	30.79	28.70	**30.90**	33.38	30.89	**33.64**	37.11
**Handball**	22.71	**26.25**	29.06	26.42	**28.97**	31.22	29.00	**30.86**	32.72	30.50	**32.75**	35.30	32.66	**35.46**	39.01
**Hockey Rink**	23.49	**25.41**	27.04	26.37	**27.75**	29.01	28.37	**29.38**	30.39	29.75	**31.01**	32.39	31.72	**33.34**	35.27
**Korfball**	18.41	**22.85**	26.27	22.30	**25.51**	28.30	25.00	**27.36**	29.72	26.41	**29.21**	32.42	28.45	**31.87**	36.31
**Modern Pentathlon**	20.23	**24.20**	27.28	23.98	**26.84**	29.33	26.58	**28.67**	30.76	28.00	**30.50**	33.36	30.06	**33.14**	37.10
**Motorsport**	NA	**NA**	NA	NA	**NA**	NA	NA	**NA**	NA	NA	**NA**	NA	NA	**NA**	NA
**Other combat sports**	22.69	**25.20**	27.25	25.76	**27.56**	29.18	27.89	**29.21**	30.52	29.24	**30.85**	32.65	31.17	**33.21**	35.72
**Rowing**	23.58	**26.06**	28.06	26.51	**28.29**	29.88	28.54	**29.85**	31.15	29.81	**31.40**	33.18	31.63	**33.64**	36.11
**Rugby**	25.15	**28.02**	30.48	30.03	**32.08**	33.96	33.43	**34.90**	36.38	35.85	**37.72**	39.78	39.33	**41.78**	44.66
**Sailing**	21.65	**24.62**	27.07	25.69	**27.81**	29.72	28.50	**30.03**	31.56	30.34	**32.25**	34.37	33.00	**35.44**	38.41
**Soccer**	21.14	**24.86**	27.79	24.90	**27.57**	29.93	27.51	**29.46**	31.41	28.99	**31.34**	34.02	31.13	**34.06**	37.77
**Surf**	NA	**NA**	NA	NA	**NA**	NA	NA	**NA**	NA	NA	**NA**	NA	NA	**NA**	NA
**Swimming**	23.51	**25.70**	27.52	26.61	**28.18**	29.60	28.76	**29.90**	31.05	30.21	**31.63**	33.20	32.29	**34.11**	36.30
**Tennis**	20.14	**23.60**	26.34	23.78	**26.26**	28.45	26.31	**28.12**	29.92	27.78	**29.97**	32.46	29.90	**32.64**	36.10
**Triathlon**	22.96	**24.84**	26.38	25.33	**26.69**	27.91	26.98	**27.98**	28.98	28.05	**29.27**	30.62	29.58	**31.12**	32.99
**Volleyball**	23.15	**27.48**	30.88	27.53	**30.64**	33.36	30.57	**32.83**	35.09	32.30	**35.02**	38.13	34.78	**38.18**	42.51
**Wrestling and Judo**	23.87	**25.99**	27.82	27.48	**29.00**	30.40	29.99	**31.09**	32.20	31.78	**33.19**	34.71	34.37	**36.19**	38.32

NA: data not presented for n < 8.

Table 10 – Arm muscle circumference (cm) percentiles by sport and sex

Sport	0.05			0.25			Median			0.75			0.95		
	Low	Estimate	High	Low	Estimate	High	Low	Estimate	High	Low	Estimate	High	Low	Estimate	High
**Females**															
Archery and Shooting	NA	**NA**	NA	NA	**NA**	NA	NA	**NA**	NA	NA	**NA**	NA	NA	**NA**	NA
Athletics	17.93	**19.80**	21.32	20.09	**21.45**	22.66	21.59	**22.59**	23.59	22.52	**23.74**	25.09	23.87	**25.39**	27.25
Basketball	17.42	**18.94**	20.21	19.52	**20.62**	21.61	20.97	**21.78**	22.58	21.95	**22.94**	24.04	23.35	**24.62**	26.13
Fencing	NA	**NA**	NA	NA	**NA**	NA	NA	**NA**	NA	NA	**NA**	NA	NA	**NA**	NA
Gymnastics	15.73	**18.09**	19.98	18.18	**19.90**	21.41	19.89	**21.15**	22.41	20.89	**22.41**	24.12	22.33	**24.21**	26.57
Handball	NA	**NA**	NA	NA	**NA**	NA	NA	**NA**	NA	NA	**NA**	NA	NA	**NA**	NA
Hockey Rink	NA	**NA**	NA	NA	**NA**	NA	NA	**NA**	NA	NA	**NA**	NA	NA	**NA**	NA
Korfball	15.82	**18.53**	20.66	18.03	**20.04**	21.81	19.57	**21.09**	22.62	20.37	**22.14**	24.15	21.53	**23.65**	26.36
Modern Pentathlon	15.19	**18.69**	21.42	17.98	**20.57**	22.85	19.92	**21.88**	23.84	20.91	**23.19**	25.78	22.34	**25.07**	28.57
Motorsport	NA	**NA**	NA	NA	**NA**	NA	NA	**NA**	NA	NA	**NA**	NA	NA	**NA**	NA
Other combat sports	16.75	**19.60**	21.84	19.25	**21.34**	23.19	20.99	**22.56**	24.13	21.92	**23.77**	25.86	23.27	**25.51**	28.36
Rowing	16.29	**19.35**	21.75	18.71	**20.98**	22.99	20.39	**22.12**	23.85	21.25	**23.25**	25.53	22.49	**24.88**	27.95
Rugby	NA	**NA**	NA	NA	**NA**	NA	NA	**NA**	NA	NA	**NA**	NA	NA	**NA**	NA
Sailing	NA	**NA**	NA	NA	**NA**	NA	NA	**NA**	NA	NA	**NA**	NA	NA	**NA**	NA
Soccer	16.29	**18.50**	20.28	18.75	**20.35**	21.77	20.46	**21.63**	22.81	21.50	**22.92**	24.52	22.99	**24.77**	26.98
Surf	NA	**NA**	NA	NA	**NA**	NA	NA	**NA**	NA	NA	**NA**	NA	NA	**NA**	NA
Swimming	18.68	**20.72**	22.38	21.08	**22.56**	23.88	22.75	**23.84**	24.92	23.79	**25.11**	26.59	25.29	**26.96**	29.00
Tennis	13.36	**17.61**	20.91	17.07	**20.16**	22.86	19.64	**21.93**	24.21	21.00	**23.70**	26.79	22.95	**26.24**	30.49
Triathlon	15.57	**19.62**	22.75	18.69	**21.69**	24.31	20.85	**23.12**	25.39	21.93	**24.56**	27.56	23.49	**26.62**	30.67
Volleyball	16.60	**19.47**	21.75	19.47	**21.55**	23.40	21.46	**23.00**	24.54	22.61	**24.45**	26.54	24.25	**26.53**	29.41
Wrestling and Judo	15.55	**18.37**	20.63	18.68	**20.71**	22.51	20.86	**22.34**	23.82	22.16	**23.97**	26.00	24.04	**26.31**	29.13
**Males**															
Archery and Shooting	15.04	**21.64**	26.00	20.18	**24.61**	28.11	23.76	**26.68**	29.58	25.22	**28.75**	33.15	27.33	**31.72**	38.29
Athletics	20.82	**23.65**	25.83	23.83	**25.81**	27.53	25.93	**27.32**	28.71	27.11	**28.83**	30.81	28.81	**30.99**	33.82
Basketball	21.33	**23.31**	24.95	24.18	**25.58**	26.85	26.16	**27.16**	28.16	27.47	**28.74**	30.14	29.37	**31.01**	32.99
Fencing	16.83	**21.48**	24.76	20.74	**23.93**	26.56	23.46	**25.63**	27.81	24.71	**27.34**	30.53	26.51	**29.79**	34.44
Gymnastics	19.94	**23.20**	25.66	23.24	**25.51**	27.46	25.54	**27.12**	28.70	26.78	**28.73**	31.00	28.58	**31.04**	34.30
Handball	20.50	**24.03**	26.68	23.99	**26.46**	28.56	26.42	**28.14**	29.86	27.72	**29.82**	32.29	29.59	**32.25**	35.78
Hockey Rink	20.58	**22.59**	24.25	23.50	**24.93**	26.21	25.53	**26.55**	27.57	26.89	**28.17**	29.60	28.85	**30.51**	32.52
Korfball	14.75	**20.77**	24.93	19.72	**23.81**	27.14	23.17	**25.93**	28.68	24.71	**28.04**	32.13	26.92	**31.08**	37.10
Modern Pentathlon	18.34	**22.79**	25.99	22.28	**25.35**	27.90	25.02	**27.13**	29.24	26.36	**28.91**	31.98	28.27	**31.47**	35.92
Motorsport	NA	**NA**	NA	NA	**NA**	NA	NA	**NA**	NA	NA	**NA**	NA	NA	**NA**	NA
Other combat sports	21.20	**23.58**	25.46	23.99	**25.66**	27.13	25.92	**27.10**	28.29	27.08	**28.55**	30.22	28.75	**30.63**	33.01
Rowing	22.79	**24.96**	26.66	25.22	**26.75**	28.09	26.91	**28.00**	29.09	27.90	**29.24**	30.78	29.33	**31.04**	33.20
Rugby	23.17	**25.58**	27.62	27.14	**28.85**	30.39	29.90	**31.11**	32.32	31.83	**33.38**	35.08	34.61	**36.64**	39.05
Sailing	18.17	**21.36**	23.92	22.40	**24.63**	26.59	25.35	**26.90**	28.45	27.21	**29.17**	31.39	29.88	**32.44**	35.63
Soccer	19.97	**23.79**	26.62	23.61	**26.26**	28.50	26.13	**27.97**	29.80	27.44	**29.68**	32.33	29.32	**32.14**	35.97
Surf	NA	**NA**	NA	NA	**NA**	NA	NA	**NA**	NA	NA	**NA**	NA	NA	**NA**	NA
Swimming	0.00	**9.46**	18.25	14.67	**22.11**	28.59	26.04	**30.91**	35.78	33.23	**39.71**	47.15	43.57	**52.36**	63.50
Tennis	17.65	**21.46**	24.31	21.46	**24.10**	26.34	24.11	**25.93**	27.75	25.52	**27.76**	30.40	27.55	**30.39**	34.20
Triathlon	21.27	**23.24**	24.83	23.70	**25.10**	26.33	25.39	**26.38**	27.38	26.43	**27.67**	29.07	27.94	**29.52**	31.50
Volleyball	22.27	**26.53**	29.68	26.32	**29.28**	31.77	29.13	**31.18**	33.23	30.59	**33.09**	36.04	32.68	**35.83**	40.09
Wrestling and Judo	21.68	**23.88**	25.75	25.36	**26.93**	28.35	27.93	**29.04**	30.15	29.73	**31.16**	32.72	32.33	**34.20**	36.41

NA: data not presented for n < 8.

Table 11 – Thigh circumference (cm) percentiles by sport and sex

Sport	0.05			0.25			Median			0.75			0.95		
	Low	Estimate	High	Low	Estimate	High	Low	Estimate	High	Low	Estimate	High	Low	Estimate	High
**Females**															
**Archery and Shooting**	NA	**NA**	NA	NA	**NA**	NA	NA	**NA**	NA	NA	**NA**	NA	NA	**NA**	NA
**Athletics**	44.00	**45.32**	46.63	47.12	**48.42**	49.73	49.29	**50.59**	51.88	51.44	**52.75**	54.05	54.54	**55.85**	57.17
**Basketball**	46.50	**47.56**	48.61	49.62	**50.67**	51.71	51.79	**52.83**	53.86	53.94	**54.99**	56.03	57.04	**58.10**	59.15
**Fencing**	NA	**NA**	NA	NA	**NA**	NA	NA	**NA**	NA	NA	**NA**	NA	NA	**NA**	NA
**Gymnastics**	41.97	**43.50**	45.03	45.09	**46.61**	48.13	47.26	**48.77**	50.28	49.41	**50.93**	52.45	52.51	**54.04**	55.56
**Handball**	NA	**NA**	NA	NA	**NA**	NA	NA	**NA**	NA	NA	**NA**	NA	NA	**NA**	NA
**Hockey Rink**	NA	**NA**	NA	NA	**NA**	NA	NA	**NA**	NA	NA	**NA**	NA	NA	**NA**	NA
**Korfball**	39.40	**41.55**	43.64	42.52	**44.66**	46.74	44.68	**46.82**	48.90	46.84	**48.98**	51.06	49.94	**52.09**	54.18
**Modern Pentathlon**	40.79	**43.04**	45.30	43.91	**46.15**	48.40	46.08	**48.31**	50.55	48.23	**50.47**	52.72	51.33	**53.58**	55.84
**Motorsport**	NA	**NA**	NA	NA	**NA**	NA	NA	**NA**	NA	NA	**NA**	NA	NA	**NA**	NA
**Other combat sports**	43.08	**45.06**	47.11	46.20	**48.17**	50.20	48.37	**50.33**	52.36	50.52	**52.49**	54.53	53.62	**55.60**	57.64
**Rowing**	44.25	**46.49**	48.73	47.37	**49.60**	51.83	49.54	**51.76**	53.99	51.69	**53.92**	56.15	54.79	**57.03**	59.27
**Rugby**	NA	**NA**	NA	NA	**NA**	NA	NA	**NA**	NA	NA	**NA**	NA	NA	**NA**	NA
**Sailing**	NA	**NA**	NA	NA	**NA**	NA	NA	**NA**	NA	NA	**NA**	NA	NA	**NA**	NA
**Soccer**	43.31	**44.71**	46.10	46.43	**47.82**	49.20	48.59	**49.98**	51.36	50.75	**52.14**	53.53	53.85	**55.25**	56.64
**Surf**	NA	**NA**	NA	NA	**NA**	NA	NA	**NA**	NA	NA	**NA**	NA	NA	**NA**	NA
**Swimming**	42.33	**43.63**	44.90	45.44	**46.74**	48.00	47.61	**48.90**	50.15	49.76	**51.06**	52.32	52.86	**54.17**	55.43
**Tennis**	42.16	**44.17**	46.18	45.28	**47.28**	49.28	47.45	**49.44**	51.43	49.60	**51.60**	53.60	52.70	**54.71**	56.72
**Triathlon**	41.27	**43.65**	46.07	44.39	**46.76**	49.17	46.56	**48.92**	51.32	48.71	**51.08**	53.49	51.81	**54.19**	56.61
**Volleyball**	45.32	**46.92**	48.55	48.44	**50.03**	51.65	50.61	**52.19**	53.81	52.76	**54.35**	55.97	55.86	**57.46**	59.09
**Wrestling and Judo**	43.63	**45.03**	46.47	46.74	**48.14**	49.57	48.91	**50.30**	51.73	51.07	**52.46**	53.89	54.17	**55.57**	57.01
**Males**															
**Archery and Shooting**	37.75	**43.89**	48.48	42.54	**46.96**	50.76	45.87	**49.09**	52.35	47.46	**51.23**	55.68	49.74	**54.29**	60.47
**Athletics**	43.47	**47.84**	51.31	48.20	**51.33**	54.09	51.48	**53.75**	56.03	53.41	**56.17**	59.31	56.20	**59.66**	64.03
**Basketball**	45.14	**48.37**	51.08	49.80	**52.13**	54.24	53.04	**54.73**	56.43	55.23	**57.34**	59.67	58.39	**61.10**	64.33
**Fencing**	41.62	**48.71**	54.04	47.75	**52.81**	57.15	52.01	**55.66**	59.31	54.17	**58.51**	63.57	57.28	**62.61**	69.70
**Gymnastics**	37.11	**42.12**	46.04	42.32	**45.90**	49.02	45.95	**48.52**	51.09	48.01	**51.14**	54.71	50.99	**54.91**	59.92
**Handball**	42.90	**48.03**	52.03	48.07	**51.74**	54.95	51.66	**54.32**	56.98	53.69	**56.90**	60.58	56.61	**60.62**	65.74
**Hockey Rink**	45.23	**48.30**	50.88	49.71	**51.92**	53.93	52.81	**54.43**	56.04	54.93	**56.94**	59.15	57.97	**60.55**	63.62
**Korfball**	37.53	**43.57**	48.09	42.58	**46.91**	50.61	46.09	**49.22**	52.36	47.84	**51.54**	55.87	50.35	**54.88**	60.92
**Modern Pentathlon**	31.70	**42.21**	50.10	41.57	**48.95**	55.25	48.43	**53.63**	58.83	52.01	**58.31**	65.69	57.16	**65.05**	75.56
**Motorsport**	NA	**NA**	NA	NA	**NA**	NA	NA	**NA**	NA	NA	**NA**	NA	NA	**NA**	NA
**Other combat sports**	43.70	**47.49**	50.54	48.19	**50.90**	53.32	51.31	**53.28**	55.25	53.24	**55.66**	58.37	56.02	**59.07**	62.86
**Rowing**	43.71	**47.80**	51.09	48.42	**51.36**	53.96	51.69	**53.82**	55.96	53.69	**56.29**	59.23	56.56	**59.84**	63.94
**Rugby**	44.54	**49.02**	52.85	52.03	**55.21**	58.16	57.24	**59.52**	61.84	60.93	**63.83**	67.05	66.23	**70.03**	74.55
**Sailing**	37.05	**43.09**	48.01	45.19	**49.45**	53.26	50.85	**53.88**	56.91	54.50	**58.31**	62.57	59.76	**64.68**	70.71
**Soccer**	42.47	**47.85**	51.98	47.67	**51.53**	54.84	51.29	**54.08**	56.84	53.28	**56.63**	60.45	56.14	**60.31**	65.65
**Surf**	NA	**NA**	NA	NA	**NA**	NA	NA	**NA**	NA	NA	**NA**	NA	NA	**NA**	NA
**Swimming**	42.95	**45.92**	48.40	47.04	**49.18**	51.12	49.89	**51.45**	53.01	51.78	**53.72**	55.86	54.50	**56.97**	59.95
**Tennis**	39.63	**44.69**	48.62	44.77	**48.37**	51.52	48.34	**50.93**	53.53	50.35	**53.50**	57.10	53.25	**57.18**	62.24
**Triathlon**	41.75	**44.84**	47.36	45.60	**47.81**	49.80	48.27	**49.88**	51.50	49.97	**51.95**	54.17	52.41	**54.93**	58.02
**Volleyball**	44.57	**50.06**	54.30	49.90	**53.82**	57.23	53.60	**56.44**	59.27	55.64	**59.05**	62.98	58.57	**62.81**	68.30
**Wrestling and Judo**	44.26	**46.81**	48.95	48.43	**50.27**	51.93	51.33	**52.68**	53.99	53.39	**55.09**	56.89	56.37	**58.55**	61.06

NA: data not presented for n < 8.

Table 12 – Thigh muscle circumference (cm) percentiles by sport and sex

Sport	0.05			0.25			Median			0.75			0.95		
	Low	Estimate	High	Low	Estimate	High	Low	Estimate	High	Low	Estimate	High	Low	Estimate	High
**Females**															
Archery and Shooting	NA	**NA**	NA	NA	**NA**	NA	NA	**NA**	NA	NA	**NA**	NA	NA	**NA**	NA
Athletics	39.20	**40.47**	41.74	42.29	**43.56**	44.83	44.44	**45.71**	46.97	46.59	**47.85**	49.12	49.67	**50.94**	52.21
Basketball	39.13	**40.15**	41.17	42.22	**43.24**	44.25	44.37	**45.38**	46.40	46.52	**47.53**	48.55	49.60	**50.62**	51.64
Fencing	NA	**NA**	NA	NA	**NA**	NA	NA	**NA**	NA	NA	**NA**	NA	NA	**NA**	NA
Gymnastics	37.70	**39.21**	40.68	40.80	**42.30**	43.77	42.95	**44.44**	45.91	45.09	**46.59**	48.06	48.18	**49.68**	51.16
Handball	NA	**NA**	NA	NA	**NA**	NA	NA	**NA**	NA	NA	**NA**	NA	NA	**NA**	NA
Hockey Rink	NA	**NA**	NA	NA	**NA**	NA	NA	**NA**	NA	NA	**NA**	NA	NA	**NA**	NA
Korfball	31.36	**33.44**	35.57	34.45	**36.53**	38.66	36.60	**38.68**	40.80	38.75	**40.82**	42.95	41.83	**43.91**	46.05
Modern Pentathlon	35.29	**37.60**	39.76	38.38	**40.69**	42.84	40.54	**42.83**	44.99	42.68	**44.98**	47.14	45.76	**48.07**	50.23
Motorsport	NA	**NA**	NA	NA	**NA**	NA	NA	**NA**	NA	NA	**NA**	NA	NA	**NA**	NA
Other combat sports	35.85	**37.88**	39.86	38.94	**40.97**	42.94	41.09	**43.12**	45.09	43.24	**45.27**	47.24	46.32	**48.36**	50.33
Rowing	37.53	**39.78**	41.99	40.63	**42.87**	45.07	42.78	**45.01**	47.22	44.92	**47.16**	49.37	48.01	**50.25**	52.46
Rugby	NA	**NA**	NA	NA	**NA**	NA	NA	**NA**	NA	NA	**NA**	NA	NA	**NA**	NA
Sailing	NA	**NA**	NA	NA	**NA**	NA	NA	**NA**	NA	NA	**NA**	NA	NA	**NA**	NA
Soccer	37.36	**38.73**	40.07	40.46	**41.82**	43.16	42.61	**43.97**	45.30	44.75	**46.12**	47.45	47.84	**49.21**	50.55
Surf	NA	**NA**	NA	NA	**NA**	NA	NA	**NA**	NA	NA	**NA**	NA	NA	**NA**	NA
Swimming	37.06	**38.27**	39.54	40.15	**41.36**	42.62	42.30	**43.51**	44.77	44.45	**45.65**	46.92	47.53	**48.74**	50.01
Tennis	32.50	**34.50**	36.48	35.59	**37.59**	39.57	37.74	**39.74**	41.71	39.89	**41.89**	43.86	42.97	**44.98**	46.96
Triathlon	35.45	**37.83**	40.23	38.55	**40.92**	43.32	40.70	**43.07**	45.46	42.84	**45.21**	47.61	45.93	**48.30**	50.71
Volleyball	38.58	**40.18**	41.74	41.68	**43.27**	44.83	43.83	**45.41**	46.97	45.97	**47.56**	49.12	49.06	**50.65**	52.22
Wrestling and Judo	36.93	**38.32**	39.70	40.03	**41.41**	42.79	42.18	**43.55**	44.93	44.32	**45.70**	47.08	47.41	**48.79**	50.18
**Males**															
Archery and Shooting	24.18	**36.14**	44.87	33.99	**42.42**	49.52	40.82	**46.79**	52.76	44.05	**51.15**	59.58	48.70	**57.44**	69.40
Athletics	38.38	**44.10**	48.61	44.69	**48.76**	52.34	49.07	**52.00**	54.93	51.66	**55.24**	59.31	55.38	**59.90**	65.62
Basketball	40.79	**44.22**	47.10	45.81	**48.27**	50.50	49.30	**51.08**	52.87	51.66	**53.90**	56.36	55.07	**57.94**	61.37
Fencing	37.79	**45.71**	51.67	44.74	**50.38**	55.22	49.57	**53.63**	57.68	52.04	**56.88**	62.52	55.59	**61.55**	69.47
Gymnastics	30.12	**37.14**	42.62	37.62	**42.57**	46.90	42.83	**46.34**	49.87	45.81	**50.11**	55.09	50.09	**55.54**	62.59
Handball	34.76	**41.52**	46.77	41.77	**46.56**	50.73	46.65	**50.06**	53.48	49.40	**53.57**	58.36	53.36	**58.61**	65.36
Hockey Rink	39.62	**42.94**	45.73	44.50	**46.89**	49.06	47.90	**49.64**	51.37	50.21	**52.38**	54.77	53.54	**56.33**	59.66
Korfball	27.41	**37.53**	45.03	36.20	**43.34**	49.41	42.32	**47.39**	52.45	45.36	**51.43**	58.57	49.74	**57.25**	67.37
Modern Pentathlon	28.57	**39.58**	47.89	39.03	**46.75**	53.38	46.30	**51.73**	57.19	50.12	**56.71**	64.46	55.60	**63.88**	74.92
Motorsport	NA	**NA**	NA	NA	**NA**	NA	NA	**NA**	NA	NA	**NA**	NA	NA	**NA**	NA
Other combat sports	38.96	**43.50**	47.17	44.43	**47.67**	50.55	48.23	**50.57**	52.91	50.58	**53.46**	56.71	53.97	**57.63**	62.18
Rowing	38.96	**43.68**	47.46	44.50	**47.86**	50.84	48.35	**50.77**	53.19	50.70	**53.67**	57.04	54.08	**57.86**	62.57
Rugby	41.15	**44.65**	47.62	46.94	**49.44**	51.73	50.96	**52.77**	54.58	53.82	**56.11**	58.61	57.92	**60.90**	64.40
Sailing	31.77	**37.77**	42.66	39.94	**44.17**	47.94	45.62	**48.62**	51.61	49.29	**53.06**	57.29	54.57	**59.46**	65.46
Soccer	37.54	**44.13**	49.22	44.04	**48.73**	52.81	48.56	**51.93**	55.31	51.06	**55.13**	59.83	54.65	**59.74**	66.33
Surf	NA	**NA**	NA	NA	**NA**	NA	NA	**NA**	NA	NA	**NA**	NA	NA	**NA**	NA
Swimming	38.14	**41.80**	44.84	43.25	**45.87**	48.24	46.81	**48.71**	50.60	49.17	**51.54**	54.16	52.57	**55.61**	59.27
Tennis	31.72	**38.81**	44.29	39.13	**44.13**	48.47	44.27	**47.82**	51.37	47.17	**51.52**	56.52	51.35	**56.83**	63.92
Triathlon	36.24	**40.56**	44.06	41.73	**44.80**	47.54	45.54	**47.75**	49.96	47.96	**50.70**	53.78	51.44	**54.95**	59.26
Volleyball	40.11	**46.22**	50.96	46.12	**50.48**	54.28	50.29	**53.44**	56.60	52.61	**56.41**	60.77	55.93	**60.67**	66.78
Wrestling and Judo	41.19	**43.74**	45.93	45.42	**47.26**	48.95	48.36	**49.71**	51.05	50.47	**52.15**	53.99	53.49	**55.67**	58.22

NA: data not presented for n < 8.

Table 13 – Calf circumference (cm) percentiles by sport and sex

Sport	0.05			0.25			Median			0.75			0.95		
	Low	Estimate	High	Low	Estimate	High	Low	Estimate	High	Low	Estimate	High	Low	Estimate	High
**Females**															
Archery and Shooting	NA	**NA**	NA	NA	**NA**	NA	NA	**NA**	NA	NA	**NA**	NA	NA	**NA**	NA
Athletics	30.07	**32.27**	34.02	32.48	**34.06**	35.45	34.16	**35.30**	36.44	35.15	**36.54**	38.12	36.58	**38.33**	40.54
Basketball	31.02	**33.30**	35.17	34.03	**35.65**	37.12	36.11	**37.29**	38.47	37.47	**38.93**	40.56	39.42	**41.29**	43.56
Fencing	NA	**NA**	NA	NA	**NA**	NA	NA	**NA**	NA	NA	**NA**	NA	NA	**NA**	NA
Gymnastics	28.20	**30.80**	32.80	30.70	**32.55**	34.16	32.43	**33.77**	35.10	33.37	**34.98**	36.84	34.74	**36.73**	39.33
Handball	NA	**NA**	NA	NA	**NA**	NA	NA	**NA**	NA	NA	**NA**	NA	NA	**NA**	NA
Hockey Rink	NA	**NA**	NA	NA	**NA**	NA	NA	**NA**	NA	NA	**NA**	NA	NA	**NA**	NA
Korfball	28.96	**32.52**	35.09	31.63	**34.17**	36.30	33.49	**35.32**	37.14	34.33	**36.46**	39.00	35.54	**38.11**	41.67
Modern Pentathlon	19.21	**27.41**	33.07	25.57	**31.20**	35.79	29.99	**33.84**	37.69	31.88	**36.48**	42.11	34.61	**40.27**	48.47
Motorsport	NA	**NA**	NA	NA	**NA**	NA	NA	**NA**	NA	NA	**NA**	NA	NA	**NA**	NA
Other combat sports	28.71	**32.63**	35.49	31.79	**34.57**	36.92	33.92	**35.92**	37.92	34.92	**37.27**	40.06	36.36	**39.21**	43.14
Rowing	27.59	**32.25**	35.55	31.06	**34.34**	37.06	33.48	**35.79**	38.11	34.53	**37.25**	40.53	36.04	**39.34**	44.00
Rugby	NA	**NA**	NA	NA	**NA**	NA	NA	**NA**	NA	NA	**NA**	NA	NA	**NA**	NA
Sailing	NA	**NA**	NA	NA	**NA**	NA	NA	**NA**	NA	NA	**NA**	NA	NA	**NA**	NA
Soccer	27.43	**30.85**	33.52	31.06	**33.48**	35.59	33.58	**35.31**	37.04	35.02	**37.13**	39.56	37.10	**39.76**	43.19
Surf	NA	**NA**	NA	NA	**NA**	NA	NA	**NA**	NA	NA	**NA**	NA	NA	**NA**	NA
Swimming	29.85	**32.02**	33.75	32.24	**33.80**	35.18	33.91	**35.04**	36.17	34.90	**36.28**	37.84	36.33	**38.06**	40.23
Tennis	29.06	**32.83**	35.58	32.01	**34.69**	36.96	34.05	**35.98**	37.91	35.01	**37.27**	39.96	36.38	**39.13**	42.90
Triathlon	28.02	**32.38**	35.44	31.11	**34.19**	36.74	33.25	**35.44**	37.64	34.15	**36.70**	39.78	35.45	**38.51**	42.87
Volleyball	28.86	**32.92**	36.00	32.66	**35.53**	38.00	35.29	**37.34**	39.40	36.69	**39.16**	42.03	38.69	**41.77**	45.83
Wrestling and Judo	28.12	**31.10**	33.43	31.20	**33.33**	35.18	33.35	**34.87**	36.39	34.56	**36.42**	38.54	36.31	**38.64**	41.62
**Males**															
Archery and Shooting	28.56	**32.25**	35.27	31.64	**34.46**	37.01	33.78	**36.00**	38.22	35.00	**37.54**	40.37	36.74	**39.76**	43.45
Athletics	31.04	**33.50**	35.56	33.83	**35.67**	37.34	35.78	**37.18**	38.58	37.01	**38.68**	40.52	38.79	**40.85**	43.32
Basketball	32.20	**34.34**	36.18	35.40	**36.97**	38.42	37.62	**38.80**	39.97	39.18	**40.62**	42.20	41.42	**43.25**	45.39
Fencing	29.94	**33.17**	35.83	32.89	**35.34**	37.55	34.95	**36.85**	38.75	36.15	**38.36**	40.81	37.87	**40.53**	43.76
Gymnastics	28.46	**31.32**	33.68	31.58	**33.70**	35.62	33.75	**35.36**	36.97	35.09	**37.02**	39.14	37.04	**39.40**	42.26
Handball	31.11	**33.93**	36.26	34.12	**36.22**	38.11	36.21	**37.81**	39.41	37.50	**39.40**	41.49	39.36	**41.68**	44.50
Hockey Rink	31.44	**33.43**	35.13	34.44	**35.90**	37.24	36.52	**37.61**	38.70	37.98	**39.33**	40.78	40.09	**41.79**	43.78
Korfball	29.97	**33.37**	36.17	32.90	**35.50**	37.84	34.94	**36.98**	39.01	36.11	**38.45**	41.05	37.79	**40.58**	43.99
Modern Pentathlon	29.32	**32.41**	34.94	32.28	**34.60**	36.70	34.34	**36.13**	37.93	35.56	**37.66**	39.98	37.32	**39.86**	42.94
Motorsport	NA	**NA**	NA	NA	**NA**	NA	NA	**NA**	NA	NA	**NA**	NA	NA	**NA**	NA
Other combat sports	30.96	**33.28**	35.23	33.83	**35.55**	37.11	35.83	**37.12**	38.42	37.13	**38.70**	40.42	39.02	**40.97**	43.29
Rowing	29.20	**32.04**	34.41	32.66	**34.75**	36.65	35.06	**36.64**	38.21	36.62	**38.52**	40.61	38.86	**41.24**	44.07
Rugby	32.87	**35.03**	36.91	36.53	**38.11**	39.56	39.07	**40.24**	41.41	40.92	**42.38**	43.95	43.57	**45.45**	47.61
Sailing	30.68	**32.82**	34.64	33.56	**35.13**	36.58	35.55	**36.74**	37.93	36.90	**38.35**	39.92	38.84	**40.66**	42.80
Soccer	29.40	**32.55**	35.14	32.64	**34.99**	37.11	34.89	**36.69**	38.49	36.27	**38.39**	40.74	38.24	**40.84**	43.98
Surf	NA	**NA**	NA	NA	**NA**	NA	NA	**NA**	NA	NA	**NA**	NA	NA	**NA**	NA
Swimming	31.42	**33.06**	34.46	33.75	**34.95**	36.06	35.36	**36.27**	37.17	36.48	**37.58**	38.79	38.08	**39.47**	41.11
Tennis	30.22	**32.74**	34.82	32.90	**34.78**	36.48	34.76	**36.19**	37.63	35.91	**37.61**	39.49	37.56	**39.64**	42.16
Triathlon	31.00	**32.72**	34.17	33.20	**34.48**	35.64	34.73	**35.70**	36.66	35.76	**36.92**	38.19	37.23	**38.68**	40.39
Volleyball	31.95	**34.83**	37.21	34.90	**37.06**	39.01	36.94	**38.60**	40.26	38.19	**40.15**	42.31	39.99	**42.37**	45.25
Wrestling and Judo	30.21	**32.01**	33.56	33.23	**34.55**	35.77	35.34	**36.32**	37.30	36.87	**38.08**	39.40	39.07	**40.63**	42.43

NA: data not presented for n < 8.

Table 14 – Calf muscle circumference (cm) percentiles by sport and sex

Sport	0.05			0.25			Median			0.75			0.95		
	Low	Estimate	High	Low	Estimate	High	Low	Estimate	High	Low	Estimate	High	Low	Estimate	High
**Females**															
Archery and Shooting	NA	**NA**	NA	NA	**NA**	NA	NA	**NA**	NA	NA	**NA**	NA	NA	**NA**	NA
Athletics	27.26	**29.66**	31.54	29.87	**31.57**	33.06	31.68	**32.90**	34.11	32.74	**34.23**	35.93	34.26	**36.14**	38.54
Basketball	24.98	**27.44**	29.44	28.26	**30.00**	31.55	30.54	**31.78**	33.01	32.00	**33.55**	35.29	34.11	**36.11**	38.57
Fencing	NA	**NA**	NA	NA	**NA**	NA	NA	**NA**	NA	NA	**NA**	NA	NA	**NA**	NA
Gymnastics	23.48	**26.43**	28.65	26.32	**28.39**	30.16	28.29	**29.75**	31.21	29.35	**31.11**	33.18	30.86	**33.08**	36.02
Handball	NA	**NA**	NA	NA	**NA**	NA	NA	**NA**	NA	NA	**NA**	NA	NA	**NA**	NA
Hockey Rink	NA	**NA**	NA	NA	**NA**	NA	NA	**NA**	NA	NA	**NA**	NA	NA	**NA**	NA
Korfball	23.61	**27.64**	30.43	26.67	**29.46**	31.73	28.79	**30.72**	32.64	29.70	**31.98**	34.77	31.00	**33.79**	37.82
Modern Pentathlon	14.80	**23.92**	29.91	21.77	**27.89**	32.70	26.62	**30.64**	34.64	28.56	**33.40**	39.49	31.35	**37.36**	46.46
Motorsport	NA	**NA**	NA	NA	**NA**	NA	NA	**NA**	NA	NA	**NA**	NA	NA	**NA**	NA
Other combat sports	25.37	**28.81**	31.24	28.01	**30.42**	32.42	29.85	**31.54**	33.23	30.67	**32.66**	35.07	31.84	**34.27**	37.71
Rowing	24.98	**28.67**	31.21	27.64	**30.21**	32.30	29.49	**31.28**	33.06	30.25	**32.34**	34.91	31.35	**33.88**	37.57
Rugby	NA	**NA**	NA	NA	**NA**	NA	NA	**NA**	NA	NA	**NA**	NA	NA	**NA**	NA
Sailing	NA	**NA**	NA	NA	**NA**	NA	NA	**NA**	NA	NA	**NA**	NA	NA	**NA**	NA
Soccer	23.14	**26.48**	29.05	26.66	**29.00**	31.02	29.10	**30.75**	32.39	30.47	**32.49**	34.84	32.44	**35.01**	38.35
Surf	NA	**NA**	NA	NA	**NA**	NA	NA	**NA**	NA	NA	**NA**	NA	NA	**NA**	NA
Swimming	25.77	**28.03**	29.81	28.26	**29.86**	31.27	29.99	**31.14**	32.29	31.01	**32.42**	34.02	32.47	**34.26**	36.52
Tennis	22.65	**26.70**	29.55	25.79	**28.61**	30.94	27.98	**29.94**	31.91	28.95	**31.27**	34.09	30.34	**33.19**	37.23
Triathlon	23.41	**28.74**	32.29	27.17	**30.82**	33.75	29.78	**32.27**	34.77	30.79	**33.72**	37.38	32.25	**35.81**	41.13
Volleyball	24.68	**28.45**	31.25	28.17	**30.81**	33.05	30.60	**32.44**	34.29	31.84	**34.08**	36.72	33.64	**36.44**	40.21
Wrestling and Judo	20.14	**24.14**	27.18	24.33	**27.11**	29.50	27.24	**29.17**	31.10	28.85	**31.23**	34.02	31.16	**34.20**	38.20
**Males**															
Archery and Shooting	25.99	**29.78**	32.70	29.01	**31.80**	34.22	31.11	**33.20**	35.29	32.17	**34.60**	37.39	33.70	**36.61**	40.40
Athletics	29.73	**32.30**	34.38	32.55	**34.42**	36.08	34.52	**35.90**	37.27	35.71	**37.37**	39.24	37.42	**39.49**	42.07
Basketball	29.94	**32.05**	33.83	33.01	**34.54**	35.94	35.15	**36.28**	37.40	36.61	**38.01**	39.54	38.72	**40.50**	42.61
Fencing	27.73	**31.27**	34.02	30.83	**33.41**	35.67	32.98	**34.90**	36.82	34.13	**36.39**	38.97	35.78	**38.53**	42.06
Gymnastics	26.52	**29.53**	31.93	29.69	**31.86**	33.79	31.89	**33.49**	35.08	33.18	**35.11**	37.28	35.04	**37.44**	40.45
Handball	29.07	**31.87**	34.10	31.94	**33.97**	35.77	33.93	**35.43**	36.93	35.09	**36.89**	38.92	36.76	**38.98**	41.78
Hockey Rink	28.09	**30.33**	32.22	31.43	**33.04**	34.51	33.75	**34.93**	36.10	35.34	**36.81**	38.42	37.64	**39.52**	41.76
Korfball	28.83	**32.33**	35.05	31.69	**34.27**	36.52	33.68	**35.62**	37.55	34.71	**36.97**	39.54	36.19	**38.90**	42.40
Modern Pentathlon	27.83	**31.03**	33.55	30.77	**33.11**	35.17	32.82	**34.56**	36.30	33.94	**36.00**	38.34	35.56	**38.08**	41.29
Motorsport	NA	**NA**	NA	NA	**NA**	NA	NA	**NA**	NA	NA	**NA**	NA	NA	**NA**	NA
Other combat sports	24.31	**28.19**	31.32	29.12	**31.88**	34.34	32.46	**34.45**	36.43	34.56	**37.02**	39.78	37.57	**40.71**	44.59
Rowing	27.43	**30.29**	32.62	30.80	**32.87**	34.72	33.15	**34.66**	36.17	34.61	**36.45**	38.52	36.70	**39.03**	41.90
Rugby	30.03	**32.05**	33.77	33.36	**34.81**	36.15	35.67	**36.74**	37.80	37.32	**38.66**	40.11	39.70	**41.42**	43.44
Sailing	26.52	**29.18**	31.38	30.06	**31.97**	33.69	32.52	**33.91**	35.29	34.12	**35.84**	37.75	36.43	**38.63**	41.29
Soccer	27.31	**30.86**	33.66	30.84	**33.41**	35.67	33.29	**35.18**	37.06	34.69	**36.95**	39.52	36.70	**39.50**	43.05
Surf	NA	**NA**	NA	NA	**NA**	NA	NA	**NA**	NA	NA	**NA**	NA	NA	**NA**	NA
Swimming	29.63	**31.28**	32.66	31.91	**33.11**	34.20	33.50	**34.38**	35.27	34.57	**35.66**	36.86	36.11	**37.49**	39.14
Tennis	28.27	**30.86**	32.93	30.91	**32.79**	34.47	32.74	**34.13**	35.53	33.80	**35.48**	37.36	35.34	**37.41**	39.99
Triathlon	29.63	**31.35**	32.77	31.78	**33.03**	34.16	33.27	**34.20**	35.12	34.24	**35.36**	36.61	35.62	**37.04**	38.76
Volleyball	30.26	**33.22**	35.58	33.16	**35.32**	37.24	35.18	**36.79**	38.39	36.33	**38.25**	40.41	37.99	**40.35**	43.32
Wrestling and Judo	27.89	**29.83**	31.48	31.11	**32.51**	33.79	33.35	**34.37**	35.39	34.96	**36.24**	37.63	37.26	**38.92**	40.85

NA: data not presented for n < 8.

Table 15 – Abdominal circumference (cm) percentiles by sport and sex

Sport	0.05			0.25			Median			0.75			0.95		
	Low	Estimate	High	Low	Estimate	High	Low	Estimate	High	Low	Estimate	High	Low	Estimate	High
**Females**															
Archery and Shooting	NA	**NA**	NA	NA	**NA**	NA	NA	**NA**	NA	NA	**NA**	NA	NA	**NA**	NA
Athletics	59.84	**64.43**	68.23	65.15	**68.54**	71.60	68.85	**71.39**	73.94	71.19	**74.25**	77.64	74.56	**78.36**	82.95
Basketball	63.58	**68.29**	72.26	70.12	**73.56**	76.69	74.67	**77.22**	79.76	77.75	**80.88**	84.31	82.17	**86.15**	90.86
Fencing	NA	**NA**	NA	NA	**NA**	NA	NA	**NA**	NA	NA	**NA**	NA	NA	**NA**	NA
Gymnastics	56.81	**62.74**	67.59	62.98	**67.36**	71.31	67.26	**70.57**	73.89	69.84	**73.79**	78.17	73.56	**78.41**	84.34
Handball	NA	**NA**	NA	NA	**NA**	NA	NA	**NA**	NA	NA	**NA**	NA	NA	**NA**	NA
Hockey Rink	NA	**NA**	NA	NA	**NA**	NA	NA	**NA**	NA	NA	**NA**	NA	NA	**NA**	NA
Korfball	60.79	**69.90**	77.20	68.39	**75.24**	81.36	73.67	**78.96**	84.25	76.56	**82.68**	89.53	80.72	**88.02**	97.13
Modern Pentathlon	53.90	**65.55**	74.84	63.44	**72.18**	79.96	70.06	**76.79**	83.52	73.62	**81.40**	90.14	78.74	**88.03**	99.67
Motorsport	NA	**NA**	NA	NA	**NA**	NA	NA	**NA**	NA	NA	**NA**	NA	NA	**NA**	NA
Other combat sports	62.22	**69.99**	76.25	69.09	**74.91**	80.11	73.87	**78.33**	82.79	76.56	**81.75**	87.57	80.42	**86.68**	94.45
Rowing	58.63	**68.66**	76.70	66.73	**74.31**	81.06	72.37	**78.23**	84.10	75.40	**82.16**	89.73	79.77	**87.80**	97.84
Rugby	NA	**NA**	NA	NA	**NA**	NA	NA	**NA**	NA	NA	**NA**	NA	NA	**NA**	NA
Sailing	NA	**NA**	NA	NA	**NA**	NA	NA	**NA**	NA	NA	**NA**	NA	NA	**NA**	NA
Soccer	61.01	**66.41**	70.86	67.04	**71.02**	74.61	71.23	**74.22**	77.21	73.83	**77.42**	81.40	77.58	**82.03**	87.44
Surf	NA	**NA**	NA	NA	**NA**	NA	NA	**NA**	NA	NA	**NA**	NA	NA	**NA**	NA
Swimming	59.67	**64.95**	69.33	65.93	**69.81**	73.31	70.28	**73.18**	76.08	73.05	**76.56**	80.43	77.04	**81.41**	86.69
Tennis	62.12	**71.13**	78.35	69.90	**76.64**	82.65	75.30	**80.47**	85.65	78.29	**84.30**	91.05	82.60	**89.82**	98.82
Triathlon	56.07	**66.63**	75.13	64.30	**72.30**	79.49	70.03	**76.25**	82.52	73.06	**80.19**	88.25	77.42	**85.87**	96.48
Volleyball	61.73	**69.44**	75.69	69.48	**75.18**	80.28	74.87	**79.17**	83.47	78.06	**83.16**	88.85	82.65	**88.90**	96.61
Wrestling and Judo	57.97	**64.23**	69.36	64.91	**69.50**	73.63	69.72	**73.16**	76.60	72.69	**76.82**	81.41	76.96	**82.09**	88.35
**Males**															
Archery and Shooting	49.48	**66.83**	79.61	63.56	**75.85**	86.31	73.34	**82.12**	90.96	78.00	**88.40**	100.75	84.70	**97.42**	114.83
Athletics	62.27	**68.01**	72.59	68.50	**72.63**	76.28	72.84	**75.84**	78.85	75.40	**79.05**	83.18	79.09	**83.67**	89.42
Basketball	65.70	**70.70**	74.90	73.01	**76.60**	79.86	78.10	**80.70**	83.31	81.55	**84.80**	88.40	86.51	**90.70**	95.71
Fencing	56.09	**67.37**	75.85	65.96	**74.00**	80.89	72.82	**78.60**	84.39	76.32	**83.21**	91.25	81.35	**89.84**	101.11
Gymnastics	58.20	**66.27**	72.59	66.60	**72.36**	77.41	72.44	**76.60**	80.76	75.79	**80.83**	86.60	80.60	**86.92**	95.00
Handball	60.08	**70.04**	77.80	70.34	**77.42**	83.61	77.47	**82.56**	87.65	81.51	**87.69**	94.77	87.32	**95.08**	105.03
Hockey Rink	65.80	**70.98**	75.31	73.45	**77.15**	80.52	78.76	**81.45**	84.13	82.38	**85.74**	89.45	87.58	**91.92**	97.09
Korfball	56.28	**67.66**	76.19	65.92	**74.05**	81.02	72.61	**78.50**	84.38	75.97	**82.94**	91.08	80.80	**89.34**	100.71
Modern Pentathlon	56.11	**66.00**	73.53	65.21	**72.26**	78.34	71.53	**76.61**	81.69	74.88	**80.97**	88.02	79.70	**87.23**	97.11
Motorsport	NA	**NA**	NA	NA	**NA**	NA	NA	**NA**	NA	NA	**NA**	NA	NA	**NA**	NA
Other combat sports	63.15	**68.67**	73.13	69.59	**73.56**	77.10	74.07	**76.97**	79.86	76.83	**80.37**	84.34	80.80	**85.26**	90.79
Rowing	61.61	**69.03**	74.97	70.29	**75.58**	80.27	76.32	**80.14**	83.96	80.01	**84.70**	89.99	85.31	**91.25**	98.68
Rugby	63.86	**71.21**	77.46	76.21	**81.43**	86.21	84.79	**88.54**	92.28	90.87	**95.64**	100.86	99.61	**105.86**	113.21
Sailing	61.38	**69.02**	75.26	71.63	**77.05**	81.90	78.75	**82.64**	86.52	83.37	**88.22**	93.64	90.01	**96.25**	103.89
Soccer	59.77	**68.72**	75.66	68.55	**74.94**	80.51	74.65	**79.27**	83.89	78.02	**83.59**	89.99	82.88	**89.82**	98.76
Surf	NA	**NA**	NA	NA	**NA**	NA	NA	**NA**	NA	NA	**NA**	NA	NA	**NA**	NA
Swimming	63.41	**68.45**	72.63	70.42	**74.03**	77.29	75.29	**77.91**	80.53	78.53	**81.79**	85.40	83.19	**87.37**	92.41
Tennis	61.96	**69.47**	75.34	69.56	**74.94**	79.66	74.84	**78.75**	82.65	77.84	**82.55**	87.93	82.15	**88.03**	95.53
Triathlon	63.19	**68.04**	72.01	69.22	**72.72**	75.86	73.42	**75.97**	78.53	76.09	**79.22**	82.72	79.93	**83.90**	88.76
Volleyball	64.47	**73.69**	80.83	73.51	**80.09**	85.83	79.79	**84.54**	89.30	83.26	**89.00**	95.58	88.26	**95.40**	104.62
Wrestling and Judo	62.97	**67.21**	70.79	69.98	**73.04**	75.80	74.86	**77.10**	79.28	78.35	**81.15**	84.16	83.35	**86.99**	91.18

NA: data not presented for n < 8.

Table 16 – Hip circumference (cm) percentiles by sport and sex

Sport	0.05			0.25			Median			0.75			0.95		
	Low	Estimate	High	Low	Estimate	High	Low	Estimate	High	Low	Estimate	High	Low	Estimate	High
**Females**															
Archery and Shooting	NA	**NA**	NA	NA	**NA**	NA	NA	**NA**	NA	NA	**NA**	NA	NA	**NA**	NA
Athletics	80.60	**84.11**	87.47	85.93	**88.95**	91.91	89.64	**92.31**	94.99	92.72	**95.68**	98.69	97.16	**100.52**	104.03
Basketball	89.25	**92.40**	95.41	94.78	**97.44**	100.04	98.62	**100.94**	103.26	101.83	**104.44**	107.10	106.46	**109.47**	112.62
Fencing	NA	**NA**	NA	NA	**NA**	NA	NA	**NA**	NA	NA	**NA**	NA	NA	**NA**	NA
Gymnastics	79.56	**83.60**	87.48	85.06	**88.58**	92.04	88.89	**92.05**	95.21	92.06	**95.51**	99.03	96.62	**100.50**	104.54
Handball	NA	**NA**	NA	NA	**NA**	NA	NA	**NA**	NA	NA	**NA**	NA	NA	**NA**	NA
Hockey Rink	NA	**NA**	NA	NA	**NA**	NA	NA	**NA**	NA	NA	**NA**	NA	NA	**NA**	NA
Korfball	80.01	**85.18**	90.19	85.47	**90.11**	94.68	89.27	**93.54**	97.81	92.40	**96.97**	101.61	96.90	**101.90**	107.08
Modern Pentathlon	80.63	**86.04**	91.28	86.11	**90.98**	95.78	89.92	**94.42**	98.92	93.05	**97.85**	102.73	97.56	**102.80**	108.21
Motorsport	NA	**NA**	NA	NA	**NA**	NA	NA	**NA**	NA	NA	**NA**	NA	NA	**NA**	NA
Other combat sports	81.12	**85.93**	90.51	86.54	**90.83**	94.99	90.31	**94.24**	98.10	93.42	**97.64**	101.87	97.89	**102.54**	107.29
Rowing	82.66	**88.09**	93.36	88.13	**93.03**	97.86	91.93	**96.46**	100.99	95.05	**99.89**	104.79	99.55	**104.82**	110.26
Rugby	NA	**NA**	NA	NA	**NA**	NA	NA	**NA**	NA	NA	**NA**	NA	NA	**NA**	NA
Sailing	NA	**NA**	NA	NA	**NA**	NA	NA	**NA**	NA	NA	**NA**	NA	NA	**NA**	NA
Soccer	83.52	**87.30**	90.92	89.05	**92.31**	95.51	92.89	**95.79**	98.70	96.08	**99.27**	102.54	100.66	**104.29**	108.06
Surf	NA	**NA**	NA	NA	**NA**	NA	NA	**NA**	NA	NA	**NA**	NA	NA	**NA**	NA
Swimming	82.49	**85.92**	89.21	87.81	**90.76**	93.64	91.52	**94.12**	96.72	94.59	**97.48**	100.42	99.02	**102.31**	105.75
Tennis	84.83	**89.81**	94.63	90.28	**94.74**	99.12	94.07	**98.16**	102.25	97.20	**101.59**	106.04	101.69	**106.51**	111.49
Triathlon	78.88	**84.53**	90.06	84.34	**89.45**	94.54	88.13	**92.87**	97.66	91.25	**96.28**	101.45	95.73	**101.20**	106.91
Volleyball	88.24	**92.62**	96.76	93.86	**97.71**	101.42	97.77	**101.24**	104.65	101.00	**104.78**	108.56	105.66	**109.87**	114.18
Wrestling and Judo	82.11	**85.89**	89.52	87.55	**90.82**	94.03	91.33	**94.25**	97.17	94.47	**97.68**	100.96	98.99	**102.62**	106.40
**Males**															
Archery and Shooting	79.74	**87.90**	94.55	86.43	**92.66**	98.27	91.08	**95.97**	100.86	93.66	**99.27**	105.50	97.39	**104.03**	112.19
Athletics	83.42	**88.31**	92.37	88.88	**92.52**	95.82	92.68	**95.45**	98.21	95.08	**98.37**	102.01	98.52	**102.58**	107.47
Basketball	86.43	**90.66**	94.29	92.63	**95.75**	98.63	96.94	**99.29**	101.64	99.95	**102.83**	105.95	104.28	**107.91**	112.15
Fencing	78.82	**87.03**	93.72	86.26	**92.44**	98.00	91.43	**96.21**	100.98	94.41	**99.97**	106.16	98.70	**105.39**	113.60
Gymnastics	78.19	**84.09**	88.97	84.50	**88.90**	92.88	88.89	**92.24**	95.59	91.61	**95.59**	99.98	95.51	**100.39**	106.30
Handball	84.23	**90.97**	96.53	91.30	**96.33**	100.87	96.22	**100.05**	103.88	99.23	**103.77**	108.80	103.57	**109.13**	115.87
Hockey Rink	86.10	**89.93**	93.21	91.76	**94.58**	97.18	95.70	**97.82**	99.94	98.46	**101.06**	103.88	102.43	**105.71**	109.54
Korfball	77.25	**85.30**	91.85	84.32	**90.41**	95.88	89.23	**93.96**	98.69	92.03	**97.51**	103.60	96.06	**102.62**	110.67
Modern Pentathlon	78.05	**85.26**	91.15	84.91	**90.32**	95.18	89.67	**93.83**	97.99	92.48	**97.35**	102.75	96.51	**102.40**	109.61
Motorsport	NA	**NA**	NA	NA	**NA**	NA	NA	**NA**	NA	NA	**NA**	NA	NA	**NA**	NA
Other combat sports	83.41	**87.72**	91.33	88.62	**91.81**	94.73	92.23	**94.66**	97.09	94.59	**97.51**	100.71	97.99	**101.60**	105.91
Rowing	82.97	**88.29**	92.74	89.28	**93.23**	96.82	93.66	**96.66**	99.65	96.50	**100.09**	104.04	100.57	**105.02**	110.35
Rugby	86.08	**91.45**	96.08	95.11	**99.01**	102.60	101.38	**104.26**	107.14	105.91	**109.51**	113.41	112.44	**117.07**	122.43
Sailing	81.73	**87.05**	91.55	88.91	**92.82**	96.40	93.90	**96.83**	99.77	97.26	**100.84**	104.76	102.11	**106.61**	111.94
Soccer	80.63	**87.25**	92.70	87.31	**92.27**	96.74	91.96	**95.75**	99.55	94.76	**99.24**	104.19	98.80	**104.25**	110.87
Surf	NA	**NA**	NA	NA	**NA**	NA	NA	**NA**	NA	NA	**NA**	NA	NA	**NA**	NA
Swimming	82.85	**86.74**	90.06	88.29	**91.16**	93.80	92.06	**94.23**	96.40	94.66	**97.30**	100.17	98.40	**101.72**	105.60
Tennis	80.79	**86.40**	91.04	86.61	**90.81**	94.61	90.66	**93.88**	97.10	93.14	**96.95**	101.14	96.72	**101.36**	106.96
Triathlon	77.69	**82.40**	86.37	83.72	**87.20**	90.37	87.91	**90.54**	93.16	90.70	**93.87**	97.35	94.71	**98.68**	103.39
Volleyball	90.64	**96.01**	100.45	95.98	**100.03**	103.68	99.70	**102.82**	105.93	101.95	**105.61**	109.65	105.19	**109.62**	115.00
Wrestling and Judo	81.70	**85.15**	88.16	87.44	**89.98**	92.33	91.43	**93.33**	95.23	94.33	**96.68**	99.22	98.51	**101.51**	104.96

NA: data not presented for n < 8.
