# Supplementary material for: Reference Values for Body Composition and Anthropometric Measurements in Athletes
Source: PLoS One. 2014 May 15;9(5):e97846. doi: 10.1371/journal.pone.0097846 (PMC4022746; doi:10.1371/journal.pone.0097846)
Supplement: File S2 — Dual Energy X-ray Absorptiometry variables percentiles by sport and sex. (PDF) [file pone.0097846.s002.pdf]

Supporting Information 2 (SI2):

Dual Energy X-ray Absorptiometry variables percentiles by sport and sex

Contents

Table 1 – Whole-body bone mineral content (g) percentiles by sport and sex 1
Table 2 – Whole-body bone mineral density (g/cm^3) percentiles by sport and sex 2
Table 3 – Whole-body fat mass (kg) percentiles by sport and sex 3
Table 4 – Whole-body fat mass index (kg/m^2) percentiles by sport and sex 4
Table 5 – Whole body fat mass (%) percentiles by sport and sex 5
Table 6 – Whole-body fat-free mass (kg) percentiles by sport and sex 6
Table 7 – Whole-body fat-free mass index (kg/m^2) percentiles by sport and sex 7
Table 8 – Whole-body lean soft tissue (kg) percentiles by sport and sex 8
Table 9 – Subtotal* bone mineral content (g) percentiles by sport and sex 9
Table 10 – Subtotal* fat mass (kg) percentiles by sport and sex 10
Table 11 – Subtotal* fat mass (%) percentiles by sport and sex 11
Table 12 – Subtotal* fat-free mass (kg) percentiles by sport and sex 12
Table 13 – Subtotal* lean soft tissue (kg) percentiles by sport and sex 13
Table 14 – Appendicular* bone mineral content (g) percentiles by sport and sex 14
Table 15 – Appendicular* fat-mass (kg) percentiles by sport and sex 15
Table 16 – Appendicular* fat mass (%) percentiles by sport and sex 16
Table 17 – Appendicular* fat-free mass (kg) percentiles by sport and sex 17
Table 18 – Appendicular* lean soft tissue (kg) percentiles by sport and sex 18
Table 19 – Appendicular* lean soft tissue index (kg/m^2) percentiles by sport and sex 19
Table 20 – Trunk bone mineral content (g) percentiles by sport and sex 20
Table 21 – Trunk fat mass (kg) percentiles by sport and sex 21
Table 22 – Trunk fat mass (%) percentiles by sport and sex 22
Table 23 – Trunk fat-free mass (kg) percentiles by sport and sex 23
Table 24 – Trunk lean soft tissue (kg) percentiles by sport and sex 24

Table 1 – Whole-body bone mineral content (g) percentiles by sport and sex

Sport	0.05			0.25			Median			0.75			0.95		
	Low	Estimate	High	Low	Estimate	High	Low	Estimate	High	Low	Estimate	High	Low	Estimate	High
**Females**															
**Archery and Shooting**	NA	**NA**	NA	NA	**NA**	NA	NA	**NA**	NA	NA	**NA**	NA	NA	**NA**	NA
**Athletics**	967	**1692**	2264	1741	**2257**	2711	2279	**2650**	3022	2590	**3043**	3560	3037	**3609**	4334
**Basketball**	1265	**1757**	2163	1958	**2305**	2618	2440	**2686**	2934	2756	**3067**	3416	3211	**3615**	4109
**Fencing**	NA	**NA**	NA	NA	**NA**	NA	NA	**NA**	NA	NA	**NA**	NA	NA	**NA**	NA
**Gymnastics**	280	**1182**	1879	1169	**1810**	2365	1787	**2246**	2703	2125	**2682**	3321	2612	**3310**	4211
**Handball**	NA	**NA**	NA	NA	**NA**	NA	NA	**NA**	NA	NA	**NA**	NA	NA	**NA**	NA
**Hockey Rink**	NA	**NA**	NA	NA	**NA**	NA	NA	**NA**	NA	NA	**NA**	NA	NA	**NA**	NA
**Korfball**	NA	**NA**	NA	NA	**NA**	NA	NA	**NA**	NA	NA	**NA**	NA	NA	**NA**	NA
**Modern Pentathlon**	NA	**NA**	NA	NA	**NA**	NA	NA	**NA**	NA	NA	**NA**	NA	NA	**NA**	NA
**Motorsport**	NA	**NA**	NA	NA	**NA**	NA	NA	**NA**	NA	NA	**NA**	NA	NA	**NA**	NA
**Other combat sports**	NA	**NA**	NA	NA	**NA**	NA	NA	**NA**	NA	NA	**NA**	NA	NA	**NA**	NA
**Rowing**	NA	**NA**	NA	NA	**NA**	NA	NA	**NA**	NA	NA	**NA**	NA	NA	**NA**	NA
**Rugby**	NA	**NA**	NA	NA	**NA**	NA	NA	**NA**	NA	NA	**NA**	NA	NA	**NA**	NA
**Sailing**	NA	**NA**	NA	NA	**NA**	NA	NA	**NA**	NA	NA	**NA**	NA	NA	**NA**	NA
**Soccer**	NA	**NA**	NA	NA	**NA**	NA	NA	**NA**	NA	NA	**NA**	NA	NA	**NA**	NA
**Surf**	NA	**NA**	NA	NA	**NA**	NA	NA	**NA**	NA	NA	**NA**	NA	NA	**NA**	NA
**Swimming**	731	**1326**	1801	1444	**1863**	2233	1939	**2237**	2534	2240	**2610**	3030	2672	**3147**	3743
**Tennis**	NA	**NA**	NA	NA	**NA**	NA	NA	**NA**	NA	NA	**NA**	NA	NA	**NA**	NA
**Triathlon**	169	**1148**	1902	1084	**1782**	2388	1720	**2223**	2725	2058	**2663**	3362	2544	**3298**	4277
**Volleyball**	1027	**1731**	2288	1778	**2281**	2723	2300	**2663**	3026	2603	**3045**	3548	3038	**3595**	4299
**Wrestling and Judo**	822	**1577**	2171	1614	**2152**	2624	2165	**2552**	2938	2480	**2951**	3489	2932	**3526**	4281
**Males**															
**Archery and Shooting**	NA	**NA**	NA	NA	**NA**	NA	NA	**NA**	NA	NA	**NA**	NA	NA	**NA**	NA
**Athletics**	1656	**2214**	2686	2210	**2638**	3030	2596	**2933**	3270	2835	**3227**	3655	3179	**3651**	4210
**Basketball**	2092	**2453**	2765	2673	**2937**	3180	3078	**3273**	3468	3366	**3609**	3872	3781	**4093**	4454
**Fencing**	NA	**NA**	NA	NA	**NA**	NA	NA	**NA**	NA	NA	**NA**	NA	NA	**NA**	NA
**Gymnastics**	NA	**NA**	NA	NA	**NA**	NA	NA	**NA**	NA	NA	**NA**	NA	NA	**NA**	NA
**Handball**	2112	**2500**	2831	2681	**2967**	3227	3077	**3291**	3502	3352	**3615**	3898	3748	**4081**	4467
**Hockey Rink**	NA	**NA**	NA	NA	**NA**	NA	NA	**NA**	NA	NA	**NA**	NA	NA	**NA**	NA
**Korfball**	NA	**NA**	NA	NA	**NA**	NA	NA	**NA**	NA	NA	**NA**	NA	NA	**NA**	NA
**Modern Pentathlon**	NA	**NA**	NA	NA	**NA**	NA	NA	**NA**	NA	NA	**NA**	NA	NA	**NA**	NA
**Motorsport**	NA	**NA**	NA	NA	**NA**	NA	NA	**NA**	NA	NA	**NA**	NA	NA	**NA**	NA
**Other combat sports**	1523	**2045**	2484	2071	**2466**	2828	2452	**2760**	3068	2691	**3053**	3449	3035	**3475**	3997
**Rowing**	NA	**NA**	NA	NA	**NA**	NA	NA	**NA**	NA	NA	**NA**	NA	NA	**NA**	NA
**Rugby**	2240	**2590**	2891	2768	**3025**	3261	3135	**3327**	3519	3393	**3629**	3886	3764	**4064**	4414
**Sailing**	NA	**NA**	NA	NA	**NA**	NA	NA	**NA**	NA	NA	**NA**	NA	NA	**NA**	NA
**Soccer**	1953	**2305**	2606	2425	**2686**	2925	2752	**2950**	3147	2975	**3214**	3475	3294	**3595**	3947
**Surf**	NA	**NA**	NA	NA	**NA**	NA	NA	**NA**	NA	NA	**NA**	NA	NA	**NA**	NA
**Swimming**	1504	**1856**	2157	2025	**2283**	2520	2388	**2580**	2771	2640	**2877**	3134	3002	**3304**	3656
**Tennis**	1371	**1870**	2293	1869	**2251**	2601	2214	**2515**	2816	2429	**2779**	3161	2737	**3159**	3659
**Triathlon**	1586	**1861**	2096	1998	**2199**	2384	2284	**2434**	2584	2483	**2668**	2870	2771	**3006**	3281
**Volleyball**	2300	**2854**	3323	2928	**3345**	3728	3364	**3687**	4010	3645	**4028**	4446	4050	**4519**	5073
**Wrestling and Judo**	1999	**2312**	2581	2497	**2726**	2936	2844	**3013**	3183	3090	**3301**	3529	3445	**3715**	4028

NA: data not presented for n < 8.

Table 2 – Whole-body bone mineral density (g/cm^3) percentiles by sport and sex

Sport	0.05			0.25			Median			0.75			0.95		
	Low	Estimate	High	Low	Estimate	High	Low	Estimate	High	Low	Estimate	High	Low	Estimate	High
**Females**															
Archery and Shooting	NA	**NA**	NA	NA	**NA**	NA	NA	**NA**	NA	NA	**NA**	NA	NA	**NA**	NA
Athletics	0.863	**0.997**	1.115	1.015	**1.121**	1.221	1.137	**1.216**	1.301	1.211	**1.319**	1.457	1.327	**1.483**	1.714
Basketball	0.883	**0.979**	1.063	1.026	**1.100**	1.169	1.139	**1.193**	1.249	1.217	**1.293**	1.387	1.338	**1.453**	1.612
Fencing	NA	**NA**	NA	NA	**NA**	NA	NA	**NA**	NA	NA	**NA**	NA	NA	**NA**	NA
Gymnastics	0.781	**0.908**	1.013	0.911	**1.007**	1.095	1.014	**1.083**	1.156	1.071	**1.164**	1.287	1.157	**1.291**	1.501
Handball	NA	**NA**	NA	NA	**NA**	NA	NA	**NA**	NA	NA	**NA**	NA	NA	**NA**	NA
Hockey Rink	NA	**NA**	NA	NA	**NA**	NA	NA	**NA**	NA	NA	**NA**	NA	NA	**NA**	NA
Korfball	NA	**NA**	NA	NA	**NA**	NA	NA	**NA**	NA	NA	**NA**	NA	NA	**NA**	NA
Modern Pentathlon	NA	**NA**	NA	NA	**NA**	NA	NA	**NA**	NA	NA	**NA**	NA	NA	**NA**	NA
Motorsport	NA	**NA**	NA	NA	**NA**	NA	NA	**NA**	NA	NA	**NA**	NA	NA	**NA**	NA
Other combat sports	NA	**NA**	NA	NA	**NA**	NA	NA	**NA**	NA	NA	**NA**	NA	NA	**NA**	NA
Rowing	NA	**NA**	NA	NA	**NA**	NA	NA	**NA**	NA	NA	**NA**	NA	NA	**NA**	NA
Rugby	NA	**NA**	NA	NA	**NA**	NA	NA	**NA**	NA	NA	**NA**	NA	NA	**NA**	NA
Sailing	NA	**NA**	NA	NA	**NA**	NA	NA	**NA**	NA	NA	**NA**	NA	NA	**NA**	NA
Soccer	NA	**NA**	NA	NA	**NA**	NA	NA	**NA**	NA	NA	**NA**	NA	NA	**NA**	NA
Surf	NA	**NA**	NA	NA	**NA**	NA	NA	**NA**	NA	NA	**NA**	NA	NA	**NA**	NA
Swimming	0.847	**0.932**	1.001	0.953	**1.015**	1.070	1.034	**1.077**	1.121	1.084	**1.143**	1.217	1.159	**1.245**	1.369
Tennis	NA	**NA**	NA	NA	**NA**	NA	NA	**NA**	NA	NA	**NA**	NA	NA	**NA**	NA
Triathlon	0.767	**0.888**	0.985	0.883	**0.973**	1.053	0.974	**1.036**	1.102	1.020	**1.104**	1.216	1.091	**1.210**	1.401
Volleyball	0.861	**0.981**	1.084	0.997	**1.090**	1.177	1.104	**1.173**	1.246	1.169	**1.262**	1.380	1.269	**1.403**	1.599
Wrestling and Judo	0.876	**1.003**	1.112	1.016	**1.115**	1.207	1.127	**1.200**	1.278	1.193	**1.292**	1.417	1.295	**1.436**	1.643
**Males**															
Archery and Shooting	NA	**NA**	NA	NA	**NA**	NA	NA	**NA**	NA	NA	**NA**	NA	NA	**NA**	NA
Athletics	1.030	**1.137**	1.237	1.147	**1.236**	1.325	1.235	**1.310**	1.390	1.295	**1.388**	1.497	1.387	**1.509**	1.666
Basketball	1.026	**1.097**	1.161	1.152	**1.208**	1.261	1.248	**1.291**	1.336	1.322	**1.381**	1.447	1.436	**1.520**	1.624
Fencing	NA	**NA**	NA	NA	**NA**	NA	NA	**NA**	NA	NA	**NA**	NA	NA	**NA**	NA
Gymnastics	NA	**NA**	NA	NA	**NA**	NA	NA	**NA**	NA	NA	**NA**	NA	NA	**NA**	NA
Handball	1.095	**1.165**	1.228	1.207	**1.263**	1.316	1.292	**1.335**	1.380	1.355	**1.412**	1.476	1.451	**1.530**	1.627
Hockey Rink	NA	**NA**	NA	NA	**NA**	NA	NA	**NA**	NA	NA	**NA**	NA	NA	**NA**	NA
Korfball	NA	**NA**	NA	NA	**NA**	NA	NA	**NA**	NA	NA	**NA**	NA	NA	**NA**	NA
Modern Pentathlon	NA	**NA**	NA	NA	**NA**	NA	NA	**NA**	NA	NA	**NA**	NA	NA	**NA**	NA
Motorsport	NA	**NA**	NA	NA	**NA**	NA	NA	**NA**	NA	NA	**NA**	NA	NA	**NA**	NA
Other combat sports	0.989	**1.085**	1.174	1.100	**1.180**	1.258	1.185	**1.250**	1.319	1.243	**1.325**	1.421	1.331	**1.440**	1.581
Rowing	NA	**NA**	NA	NA	**NA**	NA	NA	**NA**	NA	NA	**NA**	NA	NA	**NA**	NA
Rugby	1.121	**1.192**	1.256	1.238	**1.294**	1.348	1.326	**1.371**	1.416	1.393	**1.452**	1.518	1.495	**1.577**	1.676
Sailing	NA	**NA**	NA	NA	**NA**	NA	NA	**NA**	NA	NA	**NA**	NA	NA	**NA**	NA
Soccer	1.096	**1.170**	1.236	1.204	**1.263**	1.319	1.285	**1.332**	1.380	1.344	**1.405**	1.473	1.435	**1.517**	1.618
Surf	NA	**NA**	NA	NA	**NA**	NA	NA	**NA**	NA	NA	**NA**	NA	NA	**NA**	NA
Swimming	0.926	**0.993**	1.054	1.037	**1.089**	1.139	1.121	**1.161**	1.202	1.184	**1.238**	1.301	1.279	**1.357**	1.456
Tennis	0.948	**1.042**	1.128	1.051	**1.128**	1.203	1.129	**1.192**	1.258	1.181	**1.260**	1.352	1.260	**1.364**	1.499
Triathlon	0.947	**1.005**	1.057	1.044	**1.088**	1.130	1.116	**1.150**	1.184	1.170	**1.215**	1.267	1.251	**1.315**	1.395
Volleyball	1.109	**1.204**	1.293	1.227	**1.306**	1.383	1.317	**1.382**	1.450	1.381	**1.462**	1.556	1.477	**1.586**	1.723
Wrestling and Judo	1.097	**1.166**	1.230	1.219	**1.274**	1.327	1.311	**1.354**	1.399	1.382	**1.440**	1.505	1.491	**1.572**	1.672

NA: data not presented for n < 8.

Table 3 – Whole-body fat mass (kg) percentiles by sport and sex

Sport	0.05			0.25			Median			0.75			0.95		
	Low	Estimate	High	Low	Estimate	High	Low	Estimate	High	Low	Estimate	High	Low	Estimate	High
**Females**															
Archery and Shooting	NA	**NA**	NA	NA	**NA**	NA	NA	**NA**	NA	NA	**NA**	NA	NA	**NA**	NA
Athletics	5.42	**7.64**	9.95	7.33	**9.41**	11.68	9.05	**10.87**	13.05	10.12	**12.56**	16.11	11.87	**15.46**	21.82
Basketball	8.41	**10.91**	13.49	11.52	**13.91**	16.47	14.32	**16.46**	18.92	16.45	**19.49**	23.53	20.08	**24.84**	32.21
Fencing	NA	**NA**	NA	NA	**NA**	NA	NA	**NA**	NA	NA	**NA**	NA	NA	**NA**	NA
Gymnastics	4.47	**7.34**	10.61	6.66	**9.51**	12.89	8.80	**11.39**	14.75	10.07	**13.64**	19.47	12.22	**17.68**	29.05
Handball	NA	**NA**	NA	NA	**NA**	NA	NA	**NA**	NA	NA	**NA**	NA	NA	**NA**	NA
Hockey Rink	NA	**NA**	NA	NA	**NA**	NA	NA	**NA**	NA	NA	**NA**	NA	NA	**NA**	NA
Korfball	NA	**NA**	NA	NA	**NA**	NA	NA	**NA**	NA	NA	**NA**	NA	NA	**NA**	NA
Modern Pentathlon	NA	**NA**	NA	NA	**NA**	NA	NA	**NA**	NA	NA	**NA**	NA	NA	**NA**	NA
Motorsport	NA	**NA**	NA	NA	**NA**	NA	NA	**NA**	NA	NA	**NA**	NA	NA	**NA**	NA
Other combat sports	NA	**NA**	NA	NA	**NA**	NA	NA	**NA**	NA	NA	**NA**	NA	NA	**NA**	NA
Rowing	NA	**NA**	NA	NA	**NA**	NA	NA	**NA**	NA	NA	**NA**	NA	NA	**NA**	NA
Rugby	NA	**NA**	NA	NA	**NA**	NA	NA	**NA**	NA	NA	**NA**	NA	NA	**NA**	NA
Sailing	NA	**NA**	NA	NA	**NA**	NA	NA	**NA**	NA	NA	**NA**	NA	NA	**NA**	NA
Soccer	NA	**NA**	NA	NA	**NA**	NA	NA	**NA**	NA	NA	**NA**	NA	NA	**NA**	NA
Surf	NA	**NA**	NA	NA	**NA**	NA	NA	**NA**	NA	NA	**NA**	NA	NA	**NA**	NA
Swimming	8.17	**10.29**	12.35	10.26	**12.13**	14.06	12.02	**13.61**	15.40	13.16	**15.26**	18.04	15.00	**17.99**	22.66
Tennis	NA	**NA**	NA	NA	**NA**	NA	NA	**NA**	NA	NA	**NA**	NA	NA	**NA**	NA
Triathlon	3.02	**6.08**	10.09	5.18	**8.51**	12.92	7.53	**10.75**	15.34	8.94	**13.58**	22.31	11.45	**19.00**	38.25
Volleyball	6.30	**10.05**	14.34	9.58	**13.39**	17.86	12.82	**16.33**	20.82	14.94	**19.93**	27.84	18.61	**26.54**	42.32
Wrestling and Judo	4.97	**8.01**	11.50	7.53	**10.60**	14.24	10.05	**12.89**	16.53	11.66	**15.66**	22.05	14.45	**20.73**	33.39
**Males**															
Archery and Shooting	NA	**NA**	NA	NA	**NA**	NA	NA	**NA**	NA	NA	**NA**	NA	NA	**NA**	NA
Athletics	2.75	**4.88**	7.43	4.31	**6.49**	9.19	5.89	**7.92**	10.65	6.83	**9.67**	14.56	8.45	**12.87**	22.83
Basketball	5.23	**6.89**	8.67	7.65	**9.34**	11.19	9.96	**11.54**	13.36	11.90	**14.26**	17.40	15.35	**19.33**	25.45
Fencing	NA	**NA**	NA	NA	**NA**	NA	NA	**NA**	NA	NA	**NA**	NA	NA	**NA**	NA
Gymnastics	NA	**NA**	NA	NA	**NA**	NA	NA	**NA**	NA	NA	**NA**	NA	NA	**NA**	NA
Handball	4.89	**7.05**	9.52	7.73	**10.06**	12.75	10.63	**12.89**	15.62	13.02	**16.50**	21.47	17.44	**23.54**	33.94
Hockey Rink	NA	**NA**	NA	NA	**NA**	NA	NA	**NA**	NA	NA	**NA**	NA	NA	**NA**	NA
Korfball	NA	**NA**	NA	NA	**NA**	NA	NA	**NA**	NA	NA	**NA**	NA	NA	**NA**	NA
Modern Pentathlon	NA	**NA**	NA	NA	**NA**	NA	NA	**NA**	NA	NA	**NA**	NA	NA	**NA**	NA
Motorsport	NA	**NA**	NA	NA	**NA**	NA	NA	**NA**	NA	NA	**NA**	NA	NA	**NA**	NA
Other combat sports	2.91	**5.24**	8.11	4.77	**7.25**	10.35	6.72	**9.08**	12.27	7.97	**11.38**	17.29	10.17	**15.74**	28.31
Rowing	NA	**NA**	NA	NA	**NA**	NA	NA	**NA**	NA	NA	**NA**	NA	NA	**NA**	NA
Rugby	4.20	**6.69**	9.79	7.75	**10.80**	14.56	11.86	**15.08**	19.18	15.62	**21.05**	29.36	23.22	**34.01**	54.17
Sailing	NA	**NA**	NA	NA	**NA**	NA	NA	**NA**	NA	NA	**NA**	NA	NA	**NA**	NA
Soccer	3.81	**5.35**	7.03	5.58	**7.12**	8.85	7.27	**8.68**	10.38	8.53	**10.59**	13.52	10.73	**14.09**	19.79
Surf	NA	**NA**	NA	NA	**NA**	NA	NA	**NA**	NA	NA	**NA**	NA	NA	**NA**	NA
Swimming	3.54	**4.89**	6.37	5.31	**6.71**	8.28	7.04	**8.36**	9.93	8.45	**10.42**	13.16	10.97	**14.31**	19.76
Tennis	3.15	**6.49**	10.99	5.61	**9.36**	14.42	8.37	**12.07**	17.41	10.10	**15.56**	25.98	13.25	**22.44**	46.20
Triathlon	4.30	**5.38**	6.49	5.68	**6.69**	7.77	6.89	**7.79**	8.81	7.82	**9.07**	10.69	9.36	**11.28**	14.13
Volleyball	5.39	**8.16**	11.22	7.87	**10.60**	13.73	10.23	**12.71**	15.80	11.77	**15.25**	20.54	14.41	**19.80**	29.98
Wrestling and Judo	3.90	**5.10**	6.38	5.65	**6.86**	8.18	7.32	**8.43**	9.72	8.70	**10.36**	12.58	11.14	**13.94**	18.24

NA: data not presented for n < 8.

Table 4 – Whole-body fat mass index (kg/m^2) percentiles by sport and sex

Sport	0.05			0.25			Median			0.75			0.95		
	Low	Estimate	High	Low	Estimate	High	Low	Estimate	High	Low	Estimate	High	Low	Estimate	High
**Females**															
Archery and Shooting	NA	**NA**	NA	NA	**NA**	NA	NA	**NA**	NA	NA	**NA**	NA	NA	**NA**	NA
Athletics	1.07	**1.53**	1.99	1.49	**1.90**	2.33	1.87	**2.21**	2.61	2.09	**2.56**	3.28	2.45	**3.18**	4.56
Basketball	1.50	**1.90**	2.31	2.16	**2.54**	2.94	2.79	**3.12**	3.49	3.30	**3.82**	4.50	4.22	**5.12**	6.50
Fencing	NA	**NA**	NA	NA	**NA**	NA	NA	**NA**	NA	NA	**NA**	NA	NA	**NA**	NA
Gymnastics	NA	**NA**	NA	NA	**NA**	NA	NA	**NA**	NA	NA	**NA**	NA	NA	**NA**	NA
Handball	1.49	**2.06**	2.67	2.35	**2.93**	3.57	3.22	**3.75**	4.36	3.94	**4.79**	5.99	5.26	**6.82**	9.45
Hockey Rink	NA	**NA**	NA	NA	**NA**	NA	NA	**NA**	NA	NA	**NA**	NA	NA	**NA**	NA
Korfball	NA	**NA**	NA	NA	**NA**	NA	NA	**NA**	NA	NA	**NA**	NA	NA	**NA**	NA
Modern Pentathlon	NA	**NA**	NA	NA	**NA**	NA	NA	**NA**	NA	NA	**NA**	NA	NA	**NA**	NA
Motorsport	NA	**NA**	NA	NA	**NA**	NA	NA	**NA**	NA	NA	**NA**	NA	NA	**NA**	NA
Other combat sports	1.05	**1.65**	2.28	1.62	**2.20**	2.84	2.19	**2.69**	3.30	2.55	**3.29**	4.47	3.17	**4.40**	6.90
Rowing	NA	**NA**	NA	NA	**NA**	NA	NA	**NA**	NA	NA	**NA**	NA	NA	**NA**	NA
Rugby	1.31	**2.00**	2.80	2.42	**3.22**	4.14	3.70	**4.49**	5.44	4.86	**6.25**	8.32	7.19	**10.07**	15.34
Sailing	NA	**NA**	NA	NA	**NA**	NA	NA	**NA**	NA	NA	**NA**	NA	NA	**NA**	NA
Soccer	1.36	**1.77**	2.18	1.90	**2.28**	2.67	2.40	**2.71**	3.07	2.76	**3.23**	3.87	3.37	**4.15**	5.41
Surf	NA	**NA**	NA	NA	**NA**	NA	NA	**NA**	NA	NA	**NA**	NA	NA	**NA**	NA
Swimming	1.21	**1.59**	1.97	1.77	**2.13**	2.51	2.31	**2.62**	2.96	2.73	**3.21**	3.86	3.48	**4.31**	5.66
Tennis	1.02	**1.90**	2.96	1.80	**2.75**	3.89	2.67	**3.55**	4.71	3.23	**4.58**	6.98	4.25	**6.63**	12.31
Triathlon	1.46	**1.75**	2.02	1.88	**2.13**	2.38	2.25	**2.45**	2.67	2.52	**2.82**	3.19	2.97	**3.44**	4.12
Volleyball	1.39	**2.01**	2.66	2.05	**2.64**	3.28	2.69	**3.20**	3.80	3.11	**3.86**	4.98	3.84	**5.07**	7.36
Wrestling and Judo	1.41	**1.76**	2.11	1.98	**2.31**	2.64	2.51	**2.78**	3.09	2.93	**3.36**	3.91	3.67	**4.40**	5.49
**Males**															
Archery and Shooting	NA	**NA**	NA	NA	**NA**	NA	NA	**NA**	NA	NA	**NA**	NA	NA	**NA**	NA
Athletics	0.72	**1.69**	3.08	1.71	**2.99**	4.74	3.12	**4.46**	6.38	4.20	**6.65**	11.66	6.45	**11.80**	27.71
Basketball	1.90	**2.84**	3.90	3.25	**4.27**	5.43	4.72	**5.68**	6.83	5.94	**7.54**	9.92	8.27	**11.35**	16.97
Fencing	NA	**NA**	NA	NA	**NA**	NA	NA	**NA**	NA	NA	**NA**	NA	NA	**NA**	NA
Gymnastics	0.64	**1.89**	3.93	1.73	**3.49**	6.14	3.42	**5.35**	8.39	4.67	**8.21**	16.62	7.30	**15.17**	44.49
Handball	NA	**NA**	NA	NA	**NA**	NA	NA	**NA**	NA	NA	**NA**	NA	NA	**NA**	NA
Hockey Rink	NA	**NA**	NA	NA	**NA**	NA	NA	**NA**	NA	NA	**NA**	NA	NA	**NA**	NA
Korfball	NA	**NA**	NA	NA	**NA**	NA	NA	**NA**	NA	NA	**NA**	NA	NA	**NA**	NA
Modern Pentathlon	NA	**NA**	NA	NA	**NA**	NA	NA	**NA**	NA	NA	**NA**	NA	NA	**NA**	NA
Motorsport	NA	**NA**	NA	NA	**NA**	NA	NA	**NA**	NA	NA	**NA**	NA	NA	**NA**	NA
Other combat sports	NA	**NA**	NA	NA	**NA**	NA	NA	**NA**	NA	NA	**NA**	NA	NA	**NA**	NA
Rowing	NA	**NA**	NA	NA	**NA**	NA	NA	**NA**	NA	NA	**NA**	NA	NA	**NA**	NA
Rugby	NA	**NA**	NA	NA	**NA**	NA	NA	**NA**	NA	NA	**NA**	NA	NA	**NA**	NA
Sailing	NA	**NA**	NA	NA	**NA**	NA	NA	**NA**	NA	NA	**NA**	NA	NA	**NA**	NA
Soccer	NA	**NA**	NA	NA	**NA**	NA	NA	**NA**	NA	NA	**NA**	NA	NA	**NA**	NA
Surf	NA	**NA**	NA	NA	**NA**	NA	NA	**NA**	NA	NA	**NA**	NA	NA	**NA**	NA
Swimming	1.41	**2.50**	3.84	2.69	**3.97**	5.50	4.23	**5.46**	7.05	5.43	**7.53**	11.08	7.77	**11.94**	21.24
Tennis	NA	**NA**	NA	NA	**NA**	NA	NA	**NA**	NA	NA	**NA**	NA	NA	**NA**	NA
Triathlon	0.35	**1.43**	3.59	1.19	**2.94**	5.98	2.77	**4.85**	8.53	3.94	**8.01**	19.84	6.58	**16.47**	66.76
Volleyball	1.21	**2.61**	4.53	2.62	**4.38**	6.68	4.48	**6.26**	8.75	5.87	**8.96**	14.95	8.66	**15.00**	32.33
Wrestling and Judo	0.91	**2.18**	4.04	2.15	**3.84**	6.17	3.92	**5.70**	8.28	5.26	**8.45**	15.08	8.03	**14.89**	35.70

NA: data not presented for n < 8.

Table 5 – Whole body fat mass (%) percentiles by sport and sex

Sport	0.05			0.25			Median			0.75			0.95		
	Low	Estimate	High	Low	Estimate	High	Low	Estimate	High	Low	Estimate	High	Low	Estimate	High
**Females**															
Archery and Shooting	NA	**NA**	NA	NA	**NA**	NA	NA	**NA**	NA	NA	**NA**	NA	NA	**NA**	NA
Athletics	7.23	**12.49**	16.31	12.38	**15.95**	18.94	15.95	**18.36**	20.76	17.78	**20.76**	24.34	20.40	**24.23**	29.48
Basketball	13.59	**17.96**	21.44	19.38	**22.40**	25.06	23.41	**25.49**	27.58	25.93	**28.58**	31.60	29.55	**33.03**	37.40
Fencing	NA	**NA**	NA	NA	**NA**	NA	NA	**NA**	NA	NA	**NA**	NA	NA	**NA**	NA
Gymnastics	7.57	**15.35**	20.80	14.44	**19.70**	24.00	19.22	**22.72**	26.23	21.45	**25.75**	31.01	24.65	**30.10**	37.88
Handball	NA	**NA**	NA	NA	**NA**	NA	NA	**NA**	NA	NA	**NA**	NA	NA	**NA**	NA
Hockey Rink	NA	**NA**	NA	NA	**NA**	NA	NA	**NA**	NA	NA	**NA**	NA	NA	**NA**	NA
Korfball	NA	**NA**	NA	NA	**NA**	NA	NA	**NA**	NA	NA	**NA**	NA	NA	**NA**	NA
Modern Pentathlon	NA	**NA**	NA	NA	**NA**	NA	NA	**NA**	NA	NA	**NA**	NA	NA	**NA**	NA
Motorsport	NA	**NA**	NA	NA	**NA**	NA	NA	**NA**	NA	NA	**NA**	NA	NA	**NA**	NA
Other combat sports	NA	**NA**	NA	NA	**NA**	NA	NA	**NA**	NA	NA	**NA**	NA	NA	**NA**	NA
Rowing	NA	**NA**	NA	NA	**NA**	NA	NA	**NA**	NA	NA	**NA**	NA	NA	**NA**	NA
Rugby	NA	**NA**	NA	NA	**NA**	NA	NA	**NA**	NA	NA	**NA**	NA	NA	**NA**	NA
Sailing	NA	**NA**	NA	NA	**NA**	NA	NA	**NA**	NA	NA	**NA**	NA	NA	**NA**	NA
Soccer	NA	**NA**	NA	NA	**NA**	NA	NA	**NA**	NA	NA	**NA**	NA	NA	**NA**	NA
Surf	NA	**NA**	NA	NA	**NA**	NA	NA	**NA**	NA	NA	**NA**	NA	NA	**NA**	NA
Swimming	15.25	**18.73**	21.40	19.00	**21.42**	23.50	21.60	**23.29**	24.97	23.07	**25.15**	27.58	25.18	**27.84**	31.33
Tennis	NA	**NA**	NA	NA	**NA**	NA	NA	**NA**	NA	NA	**NA**	NA	NA	**NA**	NA
Triathlon	1.25	**11.46**	18.26	9.96	**16.67**	21.98	16.00	**20.28**	24.56	18.59	**23.90**	30.61	22.31	**29.10**	39.31
Volleyball	11.61	**18.17**	22.95	17.99	**22.47**	26.21	22.43	**25.46**	28.48	24.70	**28.44**	32.92	27.96	**32.74**	39.30
Wrestling and Judo	5.84	**14.10**	19.99	13.80	**19.36**	23.94	19.32	**23.01**	26.68	22.07	**26.66**	32.21	26.01	**31.92**	40.16
**Males**															
Archery and Shooting	NA	**NA**	NA	NA	**NA**	NA	NA	**NA**	NA	NA	**NA**	NA	NA	**NA**	NA
Athletics	1.17	**6.46**	9.96	5.75	**9.20**	11.92	8.93	**11.11**	13.29	10.30	**13.02**	16.47	12.26	**15.77**	21.05
Basketball	5.42	**8.53**	11.04	10.09	**12.22**	14.09	13.33	**14.78**	16.21	15.45	**17.34**	19.45	18.51	**21.02**	24.12
Fencing	NA	**NA**	NA	NA	**NA**	NA	NA	**NA**	NA	NA	**NA**	NA	NA	**NA**	NA
Gymnastics	NA	**NA**	NA	NA	**NA**	NA	NA	**NA**	NA	NA	**NA**	NA	NA	**NA**	NA
Handball	3.28	**8.01**	11.71	9.73	**12.92**	15.69	14.21	**16.34**	18.45	16.97	**19.75**	22.94	20.95	**24.66**	29.38
Hockey Rink	NA	**NA**	NA	NA	**NA**	NA	NA	**NA**	NA	NA	**NA**	NA	NA	**NA**	NA
Korfball	NA	**NA**	NA	NA	**NA**	NA	NA	**NA**	NA	NA	**NA**	NA	NA	**NA**	NA
Modern Pentathlon	NA	**NA**	NA	NA	**NA**	NA	NA	**NA**	NA	NA	**NA**	NA	NA	**NA**	NA
Motorsport	NA	**NA**	NA	NA	**NA**	NA	NA	**NA**	NA	NA	**NA**	NA	NA	**NA**	NA
Other combat sports	2.09	**7.81**	11.75	7.28	**11.08**	14.15	10.90	**13.36**	15.82	12.56	**15.63**	19.43	14.96	**18.90**	24.62
Rowing	NA	**NA**	NA	NA	**NA**	NA	NA	**NA**	NA	NA	**NA**	NA	NA	**NA**	NA
Rugby	1.93	**7.75**	12.35	10.19	**14.10**	17.51	15.92	**18.51**	21.10	19.52	**22.92**	26.84	24.68	**29.26**	35.10
Sailing	NA	**NA**	NA	NA	**NA**	NA	NA	**NA**	NA	NA	**NA**	NA	NA	**NA**	NA
Soccer	4.34	**7.51**	9.93	8.19	**10.34**	12.18	10.87	**12.31**	13.75	12.43	**14.28**	16.43	14.68	**17.11**	20.28
Surf	NA	**NA**	NA	NA	**NA**	NA	NA	**NA**	NA	NA	**NA**	NA	NA	**NA**	NA
Swimming	3.65	**6.92**	9.49	8.09	**10.31**	12.23	11.17	**12.66**	14.14	13.08	**15.02**	17.23	15.83	**18.41**	21.67
Tennis	0.00	**9.16**	15.19	7.96	**13.92**	18.58	13.51	**17.22**	20.94	15.87	**20.53**	26.48	19.26	**25.29**	34.46
Triathlon	5.98	**8.15**	9.89	8.95	**10.45**	11.76	11.02	**12.05**	13.07	12.33	**13.64**	15.14	14.21	**15.94**	18.11
Volleyball	4.05	**8.99**	12.54	8.98	**12.29**	15.03	12.41	**14.59**	16.77	14.14	**16.88**	20.19	16.64	**20.18**	25.12
Wrestling and Judo	4.65	**7.22**	9.30	8.47	**10.23**	11.79	11.13	**12.33**	13.52	12.86	**14.42**	16.18	15.35	**17.43**	20.00

NA: data not presented for n < 8.

Table 6 – Whole-body fat-free mass (kg) percentiles by sport and sex

Sport	0.05			0.25			Median			0.75			0.95		
	Low	Estimate	High	Low	Estimate	High	Low	Estimate	High	Low	Estimate	High	Low	Estimate	High
**Females**															
Archery and Shooting	NA	**NA**	NA	NA	**NA**	NA	NA	**NA**	NA	NA	**NA**	NA	NA	**NA**	NA
Athletics	37.93	**40.59**	43.26	43.16	**45.81**	48.47	46.79	**49.44**	52.09	50.41	**53.07**	55.73	55.63	**58.29**	60.96
Basketball	37.57	**39.36**	41.19	42.80	**44.58**	46.40	46.43	**48.21**	50.03	50.05	**51.84**	53.66	55.27	**57.06**	58.89
Fencing	NA	**NA**	NA	NA	**NA**	NA	NA	**NA**	NA	NA	**NA**	NA	NA	**NA**	NA
Gymnastics	26.48	**29.58**	32.63	31.71	**34.80**	37.84	35.35	**38.43**	41.47	38.97	**42.06**	45.10	44.18	**47.28**	50.33
Handball	NA	**NA**	NA	NA	**NA**	NA	NA	**NA**	NA	NA	**NA**	NA	NA	**NA**	NA
Hockey Rink	NA	**NA**	NA	NA	**NA**	NA	NA	**NA**	NA	NA	**NA**	NA	NA	**NA**	NA
Korfball	NA	**NA**	NA	NA	**NA**	NA	NA	**NA**	NA	NA	**NA**	NA	NA	**NA**	NA
Modern Pentathlon	NA	**NA**	NA	NA	**NA**	NA	NA	**NA**	NA	NA	**NA**	NA	NA	**NA**	NA
Motorsport	NA	**NA**	NA	NA	**NA**	NA	NA	**NA**	NA	NA	**NA**	NA	NA	**NA**	NA
Other combat sports	NA	**NA**	NA	NA	**NA**	NA	NA	**NA**	NA	NA	**NA**	NA	NA	**NA**	NA
Rowing	NA	**NA**	NA	NA	**NA**	NA	NA	**NA**	NA	NA	**NA**	NA	NA	**NA**	NA
Rugby	NA	**NA**	NA	NA	**NA**	NA	NA	**NA**	NA	NA	**NA**	NA	NA	**NA**	NA
Sailing	NA	**NA**	NA	NA	**NA**	NA	NA	**NA**	NA	NA	**NA**	NA	NA	**NA**	NA
Soccer	NA	**NA**	NA	NA	**NA**	NA	NA	**NA**	NA	NA	**NA**	NA	NA	**NA**	NA
Surf	NA	**NA**	NA	NA	**NA**	NA	NA	**NA**	NA	NA	**NA**	NA	NA	**NA**	NA
Swimming	33.84	**36.11**	38.39	39.06	**41.33**	43.60	42.70	**44.96**	47.22	46.32	**48.59**	50.86	51.54	**53.81**	56.09
Tennis	NA	**NA**	NA	NA	**NA**	NA	NA	**NA**	NA	NA	**NA**	NA	NA	**NA**	NA
Triathlon	31.61	**34.93**	38.34	36.84	**40.15**	43.56	40.47	**43.78**	47.18	44.09	**47.41**	50.81	49.31	**52.63**	56.04
Volleyball	37.28	**39.91**	42.60	42.51	**45.13**	47.82	46.14	**48.76**	51.44	49.77	**52.39**	55.07	54.98	**57.61**	60.30
Wrestling and Judo	32.09	**34.83**	37.59	37.32	**40.05**	42.80	40.96	**43.68**	46.43	44.58	**47.31**	50.06	49.79	**52.53**	55.29
**Males**															
Archery and Shooting	NA	**NA**	NA	NA	**NA**	NA	NA	**NA**	NA	NA	**NA**	NA	NA	**NA**	NA
Athletics	0.00	**21.95**	45.58	19.87	**42.02**	60.61	40.89	**55.97**	71.06	51.34	**69.92**	92.07	66.37	**89.99**	122.31
Basketball	29.25	**41.25**	51.15	47.74	**56.09**	63.58	60.58	**66.40**	72.22	69.22	**76.71**	85.06	81.65	**91.55**	103.55
Fencing	NA	**NA**	NA	NA	**NA**	NA	NA	**NA**	NA	NA	**NA**	NA	NA	**NA**	NA
Gymnastics	NA	**NA**	NA	NA	**NA**	NA	NA	**NA**	NA	NA	**NA**	NA	NA	**NA**	NA
Handball	24.70	**39.33**	51.16	45.20	**55.35**	64.34	59.46	**66.48**	73.50	68.62	**77.61**	87.75	81.79	**93.62**	108.26
Hockey Rink	NA	**NA**	NA	NA	**NA**	NA	NA	**NA**	NA	NA	**NA**	NA	NA	**NA**	NA
Korfball	NA	**NA**	NA	NA	**NA**	NA	NA	**NA**	NA	NA	**NA**	NA	NA	**NA**	NA
Modern Pentathlon	NA	**NA**	NA	NA	**NA**	NA	NA	**NA**	NA	NA	**NA**	NA	NA	**NA**	NA
Motorsport	NA	**NA**	NA	NA	**NA**	NA	NA	**NA**	NA	NA	**NA**	NA	NA	**NA**	NA
Other combat sports	0.00	**21.12**	42.12	20.75	**40.15**	56.55	40.20	**53.37**	66.58	50.23	**66.59**	86.03	64.66	**85.62**	114.02
Rowing	NA	**NA**	NA	NA	**NA**	NA	NA	**NA**	NA	NA	**NA**	NA	NA	**NA**	NA
Rugby	24.99	**39.50**	51.28	45.99	**56.04**	64.97	60.59	**67.54**	74.49	70.10	**79.03**	89.08	83.79	**95.57**	110.08
Sailing	NA	**NA**	NA	NA	**NA**	NA	NA	**NA**	NA	NA	**NA**	NA	NA	**NA**	NA
Soccer	17.15	**33.39**	46.22	37.84	**49.06**	58.88	52.23	**59.95**	67.68	61.02	**70.85**	82.07	73.68	**86.52**	102.76
Surf	NA	**NA**	NA	NA	**NA**	NA	NA	**NA**	NA	NA	**NA**	NA	NA	**NA**	NA
Swimming	20.09	**33.51**	44.38	38.95	**48.23**	56.49	52.06	**58.47**	64.91	60.48	**68.70**	78.02	72.59	**83.43**	96.88
Tennis	0.00	**18.51**	41.74	16.48	**38.29**	56.54	37.25	**52.04**	66.82	47.54	**65.79**	87.59	62.34	**85.57**	117.48
Triathlon	21.28	**32.93**	42.41	37.96	**46.05**	53.24	49.56	**55.16**	60.77	57.09	**64.28**	72.37	67.92	**77.40**	89.05
Volleyball	8.45	**35.70**	56.37	37.59	**56.37**	72.42	57.85	**70.74**	83.58	69.01	**85.11**	103.84	85.06	**105.78**	132.99
Wrestling and Judo	23.99	**35.41**	44.83	41.51	**49.44**	56.55	53.69	**59.19**	64.69	61.84	**68.94**	76.87	73.56	**82.97**	94.40

NA: data not presented for n < 8.

Table 7 – Whole-body fat-free mass index (kg/m^2) percentiles by sport and sex

Sport	0.05			0.25			Median			0.75			0.95		
	Low	Estimate	High	Low	Estimate	High	Low	Estimate	High	Low	Estimate	High	Low	Estimate	High
**Females**															
**Archery and Shooting**	NA	**NA**	NA	NA	**NA**	NA	NA	**NA**	NA	NA	**NA**	NA	NA	**NA**	NA
**Athletics**	13.27	**14.81**	16.31	14.88	**16.23**	17.61	16.11	**17.30**	18.57	16.99	**18.43**	20.11	18.34	**20.20**	22.55
**Basketball**	12.55	**13.56**	14.51	13.93	**14.77**	15.62	14.97	**15.68**	16.44	15.75	**16.65**	17.66	16.96	**18.14**	19.59
**Fencing**	NA	**NA**	NA	NA	**NA**	NA	NA	**NA**	NA	NA	**NA**	NA	NA	**NA**	NA
**Gymnastics**	11.48	**12.82**	14.14	12.73	**13.92**	15.14	13.68	**14.74**	15.87	14.34	**15.60**	17.06	15.35	**16.93**	18.92
**Handball**	NA	**NA**	NA	NA	**NA**	NA	NA	**NA**	NA	NA	**NA**	NA	NA	**NA**	NA
**Hockey Rink**	NA	**NA**	NA	NA	**NA**	NA	NA	**NA**	NA	NA	**NA**	NA	NA	**NA**	NA
**Korfball**	NA	**NA**	NA	NA	**NA**	NA	NA	**NA**	NA	NA	**NA**	NA	NA	**NA**	NA
**Modern Pentathlon**	NA	**NA**	NA	NA	**NA**	NA	NA	**NA**	NA	NA	**NA**	NA	NA	**NA**	NA
**Motorsport**	NA	**NA**	NA	NA	**NA**	NA	NA	**NA**	NA	NA	**NA**	NA	NA	**NA**	NA
**Other combat sports**	NA	**NA**	NA	NA	**NA**	NA	NA	**NA**	NA	NA	**NA**	NA	NA	**NA**	NA
**Rowing**	NA	**NA**	NA	NA	**NA**	NA	NA	**NA**	NA	NA	**NA**	NA	NA	**NA**	NA
**Rugby**	NA	**NA**	NA	NA	**NA**	NA	NA	**NA**	NA	NA	**NA**	NA	NA	**NA**	NA
**Sailing**	NA	**NA**	NA	NA	**NA**	NA	NA	**NA**	NA	NA	**NA**	NA	NA	**NA**	NA
**Soccer**	NA	**NA**	NA	NA	**NA**	NA	NA	**NA**	NA	NA	**NA**	NA	NA	**NA**	NA
**Surf**	NA	**NA**	NA	NA	**NA**	NA	NA	**NA**	NA	NA	**NA**	NA	NA	**NA**	NA
**Swimming**	12.84	**14.00**	15.10	14.18	**15.18**	16.17	15.20	**16.05**	16.95	15.93	**16.97**	18.16	17.05	**18.40**	20.06
**Tennis**	NA	**NA**	NA	NA	**NA**	NA	NA	**NA**	NA	NA	**NA**	NA	NA	**NA**	NA
**Triathlon**	11.92	**13.51**	15.08	13.30	**14.73**	16.20	14.36	**15.64**	17.03	15.10	**16.61**	18.38	16.22	**18.11**	20.52
**Volleyball**	12.38	**13.74**	15.05	13.79	**14.98**	16.18	14.87	**15.91**	17.02	15.64	**16.90**	18.35	16.82	**18.43**	20.45
**Wrestling and Judo**	12.11	**13.71**	15.29	13.74	**15.16**	16.63	15.00	**16.26**	17.63	15.91	**17.45**	19.25	17.30	**19.30**	21.85
**Males**															
**Archery and Shooting**	NA	**NA**	NA	NA	**NA**	NA	NA	**NA**	NA	NA	**NA**	NA	NA	**NA**	NA
**Athletics**	14.45	**16.83**	19.05	16.41	**18.43**	20.45	17.93	**19.62**	21.48	18.84	**20.90**	23.47	20.22	**22.88**	26.66
**Basketball**	15.26	**16.39**	17.42	16.87	**17.79**	18.68	18.10	**18.84**	19.61	19.00	**19.94**	21.03	20.37	**21.65**	23.26
**Fencing**	NA	**NA**	NA	NA	**NA**	NA	NA	**NA**	NA	NA	**NA**	NA	NA	**NA**	NA
**Gymnastics**	NA	**NA**	NA	NA	**NA**	NA	NA	**NA**	NA	NA	**NA**	NA	NA	**NA**	NA
**Handball**	16.59	**17.97**	19.23	18.36	**19.49**	20.58	19.70	**20.61**	21.57	20.65	**21.80**	23.14	22.09	**23.64**	25.61
**Hockey Rink**	NA	**NA**	NA	NA	**NA**	NA	NA	**NA**	NA	NA	**NA**	NA	NA	**NA**	NA
**Korfball**	NA	**NA**	NA	NA	**NA**	NA	NA	**NA**	NA	NA	**NA**	NA	NA	**NA**	NA
**Modern Pentathlon**	NA	**NA**	NA	NA	**NA**	NA	NA	**NA**	NA	NA	**NA**	NA	NA	**NA**	NA
**Motorsport**	NA	**NA**	NA	NA	**NA**	NA	NA	**NA**	NA	NA	**NA**	NA	NA	**NA**	NA
**Other combat sports**	14.09	**16.38**	18.51	16.11	**18.04**	19.98	17.68	**19.30**	21.07	18.64	**20.64**	23.13	20.12	**22.74**	26.44
**Rowing**	NA	**NA**	NA	NA	**NA**	NA	NA	**NA**	NA	NA	**NA**	NA	NA	**NA**	NA
**Rugby**	15.69	**17.47**	19.14	18.10	**19.60**	21.08	19.99	**21.23**	22.54	21.37	**22.99**	24.89	23.54	**25.79**	28.71
**Sailing**	NA	**NA**	NA	NA	**NA**	NA	NA	**NA**	NA	NA	**NA**	NA	NA	**NA**	NA
**Soccer**	16.35	**17.88**	19.28	18.12	**19.38**	20.60	19.46	**20.49**	21.57	20.38	**21.66**	23.16	21.77	**23.47**	25.67
**Surf**	NA	**NA**	NA	NA	**NA**	NA	NA	**NA**	NA	NA	**NA**	NA	NA	**NA**	NA
**Swimming**	15.24	**16.54**	17.73	16.91	**17.97**	19.00	18.18	**19.04**	19.94	19.08	**20.18**	21.44	20.46	**21.92**	23.79
**Tennis**	13.91	**16.37**	18.68	15.96	**18.05**	20.16	17.56	**19.32**	21.26	18.51	**20.68**	23.39	19.98	**22.80**	26.84
**Triathlon**	15.33	**16.45**	17.47	16.80	**17.70**	18.58	17.90	**18.63**	19.39	18.68	**19.60**	20.66	19.87	**21.09**	22.64
**Volleyball**	15.85	**17.75**	19.46	17.67	**19.23**	20.76	19.05	**20.34**	21.71	19.92	**21.51**	23.41	21.25	**23.31**	26.09
**Wrestling and Judo**	15.74	**17.26**	18.66	17.90	**19.16**	20.39	19.57	**20.60**	21.69	20.81	**22.16**	23.71	22.74	**24.60**	26.96

NA: data not presented for n < 8.

Table 8 – Whole-body lean soft tissue (kg) percentiles by sport and sex

Sport	0.05			0.25			Median			0.75			0.95		
	Low	Estimate	High	Low	Estimate	High	Low	Estimate	High	Low	Estimate	High	Low	Estimate	High
**Females**															
**Archery and Shooting**	NA	**NA**	NA	NA	**NA**	NA	NA	**NA**	NA	NA	**NA**	NA	NA	**NA**	NA
**Athletics**	35.72	**38.31**	40.90	40.71	**43.27**	45.83	44.19	**46.73**	49.26	47.62	**50.18**	52.74	52.55	**55.14**	57.73
**Basketball**	35.29	**37.08**	38.88	40.29	**42.05**	43.81	43.76	**45.50**	47.24	47.19	**48.95**	50.72	52.12	**53.92**	55.71
**Fencing**	NA	**NA**	NA	NA	**NA**	NA	NA	**NA**	NA	NA	**NA**	NA	NA	**NA**	NA
**Gymnastics**	24.67	**27.64**	30.64	29.67	**32.61**	35.58	33.14	**36.06**	39.01	36.57	**39.51**	42.48	41.51	**44.48**	47.48
**Handball**	NA	**NA**	NA	NA	**NA**	NA	NA	**NA**	NA	NA	**NA**	NA	NA	**NA**	NA
**Hockey Rink**	NA	**NA**	NA	NA	**NA**	NA	NA	**NA**	NA	NA	**NA**	NA	NA	**NA**	NA
**Korfball**	NA	**NA**	NA	NA	**NA**	NA	NA	**NA**	NA	NA	**NA**	NA	NA	**NA**	NA
**Modern Pentathlon**	NA	**NA**	NA	NA	**NA**	NA	NA	**NA**	NA	NA	**NA**	NA	NA	**NA**	NA
**Motorsport**	NA	**NA**	NA	NA	**NA**	NA	NA	**NA**	NA	NA	**NA**	NA	NA	**NA**	NA
**Other combat sports**	NA	**NA**	NA	NA	**NA**	NA	NA	**NA**	NA	NA	**NA**	NA	NA	**NA**	NA
**Rowing**	NA	**NA**	NA	NA	**NA**	NA	NA	**NA**	NA	NA	**NA**	NA	NA	**NA**	NA
**Rugby**	NA	**NA**	NA	NA	**NA**	NA	NA	**NA**	NA	NA	**NA**	NA	NA	**NA**	NA
**Sailing**	NA	**NA**	NA	NA	**NA**	NA	NA	**NA**	NA	NA	**NA**	NA	NA	**NA**	NA
**Soccer**	NA	**NA**	NA	NA	**NA**	NA	NA	**NA**	NA	NA	**NA**	NA	NA	**NA**	NA
**Surf**	NA	**NA**	NA	NA	**NA**	NA	NA	**NA**	NA	NA	**NA**	NA	NA	**NA**	NA
**Swimming**	32.03	**34.26**	36.49	37.03	**39.22**	41.42	40.50	**42.68**	44.85	43.93	**46.13**	48.32	48.87	**51.09**	53.32
**Tennis**	NA	**NA**	NA	NA	**NA**	NA	NA	**NA**	NA	NA	**NA**	NA	NA	**NA**	NA
**Triathlon**	29.83	**32.84**	36.37	34.83	**37.81**	41.31	38.30	**41.26**	44.74	41.73	**44.71**	48.21	46.67	**49.68**	53.21
**Volleyball**	35.03	**37.65**	40.23	40.03	**42.61**	45.17	43.50	**46.06**	48.60	46.93	**49.52**	52.07	51.87	**54.48**	57.07
**Wrestling and Judo**	29.96	**32.65**	35.32	34.96	**37.62**	40.25	38.43	**41.07**	43.68	41.86	**44.52**	47.16	46.80	**49.49**	52.16
**Males**															
**Archery and Shooting**	NA	**NA**	NA	NA	**NA**	NA	NA	**NA**	NA	NA	**NA**	NA	NA	**NA**	NA
**Athletics**	42.32	**51.34**	58.23	50.12	**56.63**	62.26	55.54	**60.30**	65.06	58.35	**63.98**	70.48	62.38	**69.26**	78.28
**Basketball**	46.56	**52.63**	57.69	55.60	**59.92**	63.82	61.89	**64.98**	68.08	66.14	**70.05**	74.36	72.27	**77.33**	83.40
**Fencing**	NA	**NA**	NA	NA	**NA**	NA	NA	**NA**	NA	NA	**NA**	NA	NA	**NA**	NA
**Gymnastics**	NA	**NA**	NA	NA	**NA**	NA	NA	**NA**	NA	NA	**NA**	NA	NA	**NA**	NA
**Handball**	46.37	**53.24**	58.88	55.60	**60.50**	64.88	62.03	**65.54**	69.06	66.20	**70.58**	75.48	72.20	**77.84**	84.71
**Hockey Rink**	NA	**NA**	NA	NA	**NA**	NA	NA	**NA**	NA	NA	**NA**	NA	NA	**NA**	NA
**Korfball**	NA	**NA**	NA	NA	**NA**	NA	NA	**NA**	NA	NA	**NA**	NA	NA	**NA**	NA
**Modern Pentathlon**	NA	**NA**	NA	NA	**NA**	NA	NA	**NA**	NA	NA	**NA**	NA	NA	**NA**	NA
**Motorsport**	NA	**NA**	NA	NA	**NA**	NA	NA	**NA**	NA	NA	**NA**	NA	NA	**NA**	NA
**Other combat sports**	36.02	**45.98**	53.61	45.27	**52.39**	58.54	51.70	**56.84**	61.97	55.13	**61.29**	68.40	60.06	**67.69**	77.65
**Rowing**	NA	**NA**	NA	NA	**NA**	NA	NA	**NA**	NA	NA	**NA**	NA	NA	**NA**	NA
**Rugby**	43.33	**51.34**	57.93	54.54	**60.22**	65.31	62.34	**66.39**	70.44	67.47	**72.56**	78.23	74.85	**81.44**	89.45
**Sailing**	NA	**NA**	NA	NA	**NA**	NA	NA	**NA**	NA	NA	**NA**	NA	NA	**NA**	NA
**Soccer**	44.82	**50.75**	55.53	51.98	**56.21**	59.98	56.95	**60.01**	63.07	60.04	**63.80**	68.04	64.48	**69.27**	75.19
**Surf**	NA	**NA**	NA	NA	**NA**	NA	NA	**NA**	NA	NA	**NA**	NA	NA	**NA**	NA
**Swimming**	40.76	**47.03**	52.17	49.19	**53.64**	57.64	55.04	**58.24**	61.44	58.85	**62.84**	67.30	64.32	**69.46**	75.72
**Tennis**	33.38	**45.01**	53.83	43.65	**51.96**	59.11	50.79	**56.78**	62.78	54.46	**61.61**	69.92	59.74	**68.55**	80.18
**Triathlon**	43.39	**47.50**	50.90	48.96	**51.91**	54.56	52.82	**54.97**	57.11	55.37	**58.03**	60.98	59.03	**62.43**	66.54
**Volleyball**	50.12	**59.89**	67.52	59.98	**66.97**	73.08	66.84	**71.89**	76.94	70.70	**76.81**	83.80	76.26	**83.89**	93.66
**Wrestling and Judo**	40.13	**46.08**	51.04	48.92	**53.16**	56.98	55.04	**58.07**	61.11	59.16	**62.99**	67.22	65.10	**70.06**	76.02

NA: data not presented for n < 8.

Table 9 – Subtotal* bone mineral content (g) percentiles by sport and sex

Sport	0.05			0.25			Median			0.75			0.95		
	Low	Estimate	High	Low	Estimate	High	Low	Estimate	High	Low	Estimate	High	Low	Estimate	High
**Females**															
Archery and Shooting	NA	**NA**	NA	NA	**NA**	NA	NA	**NA**	NA	NA	**NA**	NA	NA	**NA**	NA
Athletics	1324	**1844**	2258	1810	**2191**	2529	2148	**2432**	2718	2336	**2673**	3056	2608	**3020**	3542
Basketball	1655	**2003**	2297	2195	**2443**	2669	2571	**2749**	2928	2829	**3055**	3303	3202	**3495**	3843
Fencing	NA	**NA**	NA	NA	**NA**	NA	NA	**NA**	NA	NA	**NA**	NA	NA	**NA**	NA
Gymnastics	NA	**NA**	NA	NA	**NA**	NA	NA	**NA**	NA	NA	**NA**	NA	NA	**NA**	NA
Handball	1723	**2075**	2367	2218	**2469**	2696	2561	**2743**	2925	2790	**3017**	3269	3119	**3412**	3763
Hockey Rink	NA	**NA**	NA	NA	**NA**	NA	NA	**NA**	NA	NA	**NA**	NA	NA	**NA**	NA
Korfball	NA	**NA**	NA	NA	**NA**	NA	NA	**NA**	NA	NA	**NA**	NA	NA	**NA**	NA
Modern Pentathlon	NA	**NA**	NA	NA	**NA**	NA	NA	**NA**	NA	NA	**NA**	NA	NA	**NA**	NA
Motorsport	NA	**NA**	NA	NA	**NA**	NA	NA	**NA**	NA	NA	**NA**	NA	NA	**NA**	NA
Other combat sports	1145	**1646**	2043	1640	**2004**	2325	1984	**2253**	2521	2180	**2503**	2866	2463	**2861**	3361
Rowing	NA	**NA**	NA	NA	**NA**	NA	NA	**NA**	NA	NA	**NA**	NA	NA	**NA**	NA
Rugby	1831	**2143**	2405	2281	**2506**	2709	2595	**2757**	2920	2806	**3009**	3233	3110	**3371**	3684
Sailing	NA	**NA**	NA	NA	**NA**	NA	NA	**NA**	NA	NA	**NA**	NA	NA	**NA**	NA
Soccer	1604	**1913**	2167	1996	**2218**	2419	2268	**2431**	2593	2443	**2643**	2866	2694	**2948**	3257
Surf	NA	**NA**	NA	NA	**NA**	NA	NA	**NA**	NA	NA	**NA**	NA	NA	**NA**	NA
Swimming	1106	**1434**	1706	1572	**1805**	2015	1896	**2063**	2229	2110	**2320**	2553	2420	**2691**	3020
Tennis	1050	**1534**	1917	1504	**1858**	2170	1820	**2083**	2346	1996	**2309**	2663	2250	**2633**	3117
Triathlon	1232	**1457**	1645	1554	**1715**	1861	1778	**1895**	2011	1928	**2074**	2235	2144	**2332**	2557
Volleyball	1911	**2432**	2851	2468	**2846**	3182	2855	**3133**	3412	3085	**3421**	3799	3416	**3835**	4355
Wrestling and Judo	1551	**1830**	2066	1978	**2178**	2360	2276	**2420**	2564	2480	**2662**	2862	2774	**3010**	3290
**Males**															
Archery and Shooting	NA	**NA**	NA	NA	**NA**	NA	NA	**NA**	NA	NA	**NA**	NA	NA	**NA**	NA
Athletics	873	**1344**	1706	1355	**1685**	1972	1690	**1923**	2157	1875	**2161**	2492	2142	**2502**	2974
Basketball	905	**1314**	1645	1469	**1753**	2006	1861	**2059**	2257	2112	**2364**	2649	2473	**2803**	3213
Fencing	NA	**NA**	NA	NA	**NA**	NA	NA	**NA**	NA	NA	**NA**	NA	NA	**NA**	NA
Gymnastics	584	**1036**	1376	1005	**1324**	1595	1299	**1523**	1747	1451	**1723**	2040	1670	**2011**	2462
Handball	NA	**NA**	NA	NA	**NA**	NA	NA	**NA**	NA	NA	**NA**	NA	NA	**NA**	NA
Hockey Rink	NA	**NA**	NA	NA	**NA**	NA	NA	**NA**	NA	NA	**NA**	NA	NA	**NA**	NA
Korfball	NA	**NA**	NA	NA	**NA**	NA	NA	**NA**	NA	NA	**NA**	NA	NA	**NA**	NA
Modern Pentathlon	NA	**NA**	NA	NA	**NA**	NA	NA	**NA**	NA	NA	**NA**	NA	NA	**NA**	NA
Motorsport	NA	**NA**	NA	NA	**NA**	NA	NA	**NA**	NA	NA	**NA**	NA	NA	**NA**	NA
Other combat sports	NA	**NA**	NA	NA	**NA**	NA	NA	**NA**	NA	NA	**NA**	NA	NA	**NA**	NA
Rowing	NA	**NA**	NA	NA	**NA**	NA	NA	**NA**	NA	NA	**NA**	NA	NA	**NA**	NA
Rugby	NA	**NA**	NA	NA	**NA**	NA	NA	**NA**	NA	NA	**NA**	NA	NA	**NA**	NA
Sailing	NA	**NA**	NA	NA	**NA**	NA	NA	**NA**	NA	NA	**NA**	NA	NA	**NA**	NA
Soccer	NA	**NA**	NA	NA	**NA**	NA	NA	**NA**	NA	NA	**NA**	NA	NA	**NA**	NA
Surf	NA	**NA**	NA	NA	**NA**	NA	NA	**NA**	NA	NA	**NA**	NA	NA	**NA**	NA
Swimming	853	**1146.1**	1377	1187	**1394**	1575	1419	**1566**	1712	1556	**1738**	1944	1754	**1985**	2279
Tennis	NA	**NA**	NA	NA	**NA**	NA	NA	**NA**	NA	NA	**NA**	NA	NA	**NA**	NA
Triathlon	629	**1018**	1310	969	**1245**	1482	1206	**1403**	1601	1325	**1561**	1838	1496	**1788**	2178
Volleyball	928	**1373**	1716	1383	**1696**	1968	1699	**1921**	2143	1874	**2145**	2459	2125	**2468**	2914
Wrestling and Judo	796	**1255**	1605	1255	**1578**	1856	1575	**1802**	2030	1749	**2027**	2349	1999	**2349**	2808

NA: data not presented for n < 8.

*Whole-body minus the head

Table 10 – Subtotal* fat mass (kg) percentiles by sport and sex

Sport	0.05			0.25			Median			0.75			0.95		
	Low	Estimate	High	Low	Estimate	High	Low	Estimate	High	Low	Estimate	High	Low	Estimate	High
**Females**															
Archery and Shooting	NA	**NA**	NA	NA	**NA**	NA	NA	**NA**	NA	NA	**NA**	NA	NA	**NA**	NA
Athletics	3.84	**6.30**	9.17	5.98	**8.52**	11.56	8.14	**10.51**	13.58	9.56	**12.97**	18.48	12.04	**17.54**	28.80
Basketball	7.71	**10.23**	12.89	10.83	**13.30**	15.99	13.72	**15.96**	18.58	15.93	**19.16**	23.53	19.76	**24.91**	33.05
Fencing	NA	**NA**	NA	NA	**NA**	NA	NA	**NA**	NA	NA	**NA**	NA	NA	**NA**	NA
Gymnastics	3.29	**6.35**	10.27	5.62	**8.95**	13.26	8.14	**11.35**	15.84	9.72	**14.41**	22.96	12.56	**20.31**	39.17
Handball	NA	**NA**	NA	NA	**NA**	NA	NA	**NA**	NA	NA	**NA**	NA	NA	**NA**	NA
Hockey Rink	NA	**NA**	NA	NA	**NA**	NA	NA	**NA**	NA	NA	**NA**	NA	NA	**NA**	NA
Korfball	NA	**NA**	NA	NA	**NA**	NA	NA	**NA**	NA	NA	**NA**	NA	NA	**NA**	NA
Modern Pentathlon	NA	**NA**	NA	NA	**NA**	NA	NA	**NA**	NA	NA	**NA**	NA	NA	**NA**	NA
Motorsport	NA	**NA**	NA	NA	**NA**	NA	NA	**NA**	NA	NA	**NA**	NA	NA	**NA**	NA
Other combat sports	NA	**NA**	NA	NA	**NA**	NA	NA	**NA**	NA	NA	**NA**	NA	NA	**NA**	NA
Rowing	NA	**NA**	NA	NA	**NA**	NA	NA	**NA**	NA	NA	**NA**	NA	NA	**NA**	NA
Rugby	NA	**NA**	NA	NA	**NA**	NA	NA	**NA**	NA	NA	**NA**	NA	NA	**NA**	NA
Sailing	NA	**NA**	NA	NA	**NA**	NA	NA	**NA**	NA	NA	**NA**	NA	NA	**NA**	NA
Soccer	NA	**NA**	NA	NA	**NA**	NA	NA	**NA**	NA	NA	**NA**	NA	NA	**NA**	NA
Surf	NA	**NA**	NA	NA	**NA**	NA	NA	**NA**	NA	NA	**NA**	NA	NA	**NA**	NA
Swimming	6.77	**9.17**	11.63	9.14	**11.37**	13.79	11.25	**13.22**	15.53	12.66	**15.36**	19.12	15.01	**19.06**	25.79
Tennis	NA	**NA**	NA	NA	**NA**	NA	NA	**NA**	NA	NA	**NA**	NA	NA	**NA**	NA
Triathlon	1.97	**4.99**	9.68	4.05	**7.79**	13.40	6.70	**10.61**	16.81	8.40	**14.46**	27.78	11.63	**22.57**	57.24
Volleyball	5.76	**9.58**	14.08	9.10	**13.08**	17.87	12.50	**16.23**	21.09	14.75	**20.15**	28.97	18.71	**27.51**	45.75
Wrestling and Judo	4.00	**7.11**	10.95	6.65	**10.02**	14.21	9.49	**12.71**	17.04	11.37	**16.13**	24.29	14.76	**22.73**	40.44
**Males**															
Archery and Shooting	NA	**NA**	NA	NA	**NA**	NA	NA	**NA**	NA	NA	**NA**	NA	NA	**NA**	NA
Athletics	2.64	**4.27**	6.17	3.89	**5.53**	7.50	5.10	**6.62**	8.59	5.84	**7.92**	11.25	7.10	**10.25**	16.58
Basketball	4.54	**6.02**	7.64	6.77	**8.31**	10.01	8.93	**10.38**	12.08	10.77	**12.98**	15.93	14.11	**17.90**	23.74
Fencing	NA	**NA**	NA	NA	**NA**	NA	NA	**NA**	NA	NA	**NA**	NA	NA	**NA**	NA
Gymnastics	NA	**NA**	NA	NA	**NA**	NA	NA	**NA**	NA	NA	**NA**	NA	NA	**NA**	NA
Handball	4.22	**6.15**	8.41	6.81	**8.96**	11.48	9.50	**11.63**	14.25	11.79	**15.10**	19.88	16.08	**21.99**	32.10
Hockey Rink	NA	**NA**	NA	NA	**NA**	NA	NA	**NA**	NA	NA	**NA**	NA	NA	**NA**	NA
Korfball	NA	**NA**	NA	NA	**NA**	NA	NA	**NA**	NA	NA	**NA**	NA	NA	**NA**	NA
Modern Pentathlon	NA	**NA**	NA	NA	**NA**	NA	NA	**NA**	NA	NA	**NA**	NA	NA	**NA**	NA
Motorsport	NA	**NA**	NA	NA	**NA**	NA	NA	**NA**	NA	NA	**NA**	NA	NA	**NA**	NA
Other combat sports	2.67	**4.57**	6.92	4.24	**6.27**	8.81	5.84	**7.81**	10.43	6.91	**9.72**	14.38	8.81	**13.33**	22.84
Rowing	NA	**NA**	NA	NA	**NA**	NA	NA	**NA**	NA	NA	**NA**	NA	NA	**NA**	NA
Rugby	3.70	**5.93**	8.74	6.97	**9.77**	13.23	10.82	**13.82**	17.65	14.43	**19.55**	27.39	21.85	**32.22**	51.54
Sailing	NA	**NA**	NA	NA	**NA**	NA	NA	**NA**	NA	NA	**NA**	NA	NA	**NA**	NA
Soccer	3.26	**4.57**	6.01	4.79	**6.12**	7.63	6.26	**7.50**	8.99	7.38	**9.19**	11.75	9.36	**12.31**	17.25
Surf	NA	**NA**	NA	NA	**NA**	NA	NA	**NA**	NA	NA	**NA**	NA	NA	**NA**	NA
Swimming	2.98	**4.20**	5.57	4.63	**5.93**	7.41	6.28	**7.53**	9.03	7.66	**9.57**	12.26	10.18	**13.51**	19.04
Tennis	2.87	**5.65**	9.45	5.01	**8.19**	12.52	7.39	**10.60**	15.21	8.98	**13.72**	22.43	11.89	**19.88**	39.21
Triathlon	3.70	**4.62**	5.56	4.91	**5.77**	6.68	5.97	**6.73**	7.59	6.78	**7.85**	9.23	8.14	**9.79**	12.23
Volleyball	4.64	**7.07**	9.83	6.88	**9.35**	12.24	9.04	**11.35**	14.24	10.53	**13.78**	18.72	13.10	**18.22**	27.74
Wrestling and Judo	3.28	**4.34**	5.49	4.83	**5.92**	7.13	6.32	**7.35**	8.55	7.57	**9.12**	11.19	9.84	**12.45**	16.47

NA: data not presented for n < 8.

*Whole-body minus the head

Table 11 – Subtotal* fat mass (%) percentiles by sport and sex

Sport	0.05			0.25			Median			0.75			0.95		
	Low	Estimate	High	Low	Estimate	High	Low	Estimate	High	Low	Estimate	High	Low	Estimate	High
**Females**															
Archery and Shooting	NA	**NA**	NA	NA	**NA**	NA	NA	**NA**	NA	NA	**NA**	NA	NA	**NA**	NA
Athletics	4.84	**11.33**	16.00	11.24	**15.62**	19.24	15.70	**18.60**	21.49	17.95	**21.58**	25.95	21.19	**25.87**	32.35
Basketball	13.33	**18.03**	21.76	19.58	**22.82**	25.66	23.93	**26.15**	28.37	26.64	**29.48**	32.71	30.53	**34.27**	38.97
Fencing	NA	**NA**	NA	NA	**NA**	NA	NA	**NA**	NA	NA	**NA**	NA	NA	**NA**	NA
Gymnastics	6.64	**15.32**	21.37	14.34	**20.19**	24.95	19.70	**23.57**	27.44	22.18	**26.95**	32.79	25.76	**31.81**	40.50
Handball	NA	**NA**	NA	NA	**NA**	NA	NA	**NA**	NA	NA	**NA**	NA	NA	**NA**	NA
Hockey Rink	NA	**NA**	NA	NA	**NA**	NA	NA	**NA**	NA	NA	**NA**	NA	NA	**NA**	NA
Korfball	NA	**NA**	NA	NA	**NA**	NA	NA	**NA**	NA	NA	**NA**	NA	NA	**NA**	NA
Modern Pentathlon	NA	**NA**	NA	NA	**NA**	NA	NA	**NA**	NA	NA	**NA**	NA	NA	**NA**	NA
Motorsport	NA	**NA**	NA	NA	**NA**	NA	NA	**NA**	NA	NA	**NA**	NA	NA	**NA**	NA
Other combat sports	NA	**NA**	NA	NA	**NA**	NA	NA	**NA**	NA	NA	**NA**	NA	NA	**NA**	NA
Rowing	NA	**NA**	NA	NA	**NA**	NA	NA	**NA**	NA	NA	**NA**	NA	NA	**NA**	NA
Rugby	NA	**NA**	NA	NA	**NA**	NA	NA	**NA**	NA	NA	**NA**	NA	NA	**NA**	NA
Sailing	NA	**NA**	NA	NA	**NA**	NA	NA	**NA**	NA	NA	**NA**	NA	NA	**NA**	NA
Soccer	NA	**NA**	NA	NA	**NA**	NA	NA	**NA**	NA	NA	**NA**	NA	NA	**NA**	NA
Surf	NA	**NA**	NA	NA	**NA**	NA	NA	**NA**	NA	NA	**NA**	NA	NA	**NA**	NA
Swimming	14.53	**18.56**	21.63	18.89	**21.68**	24.08	21.92	**23.86**	25.79	23.63	**26.03**	28.82	26.08	**29.15**	33.19
Tennis	NA	**NA**	NA	NA	**NA**	NA	NA	**NA**	NA	NA	**NA**	NA	NA	**NA**	NA
Triathlon	0.00	**10.54**	18.43	8.82	**16.61**	22.75	15.92	**20.84**	25.76	18.92	**25.06**	32.85	23.25	**31.13**	43.05
Volleyball	11.36	**18.38**	23.51	18.21	**22.99**	27.00	22.98	**26.20**	29.43	25.41	**29.40**	34.20	28.90	**34.01**	41.05
Wrestling and Judo	4.52	**13.75**	20.33	13.45	**19.65**	24.75	19.66	**23.74**	27.82	22.73	**27.84**	34.02	27.15	**33.74**	42.95
**Males**															
Archery and Shooting	NA	**NA**	NA	NA	**NA**	NA	NA	**NA**	NA	NA	**NA**	NA	NA	**NA**	NA
Athletics	0.00	**5.48**	9.16	4.75	**8.39**	11.24	8.14	**10.42**	12.69	9.59	**12.45**	16.08	11.68	**15.37**	20.96
Basketball	4.52	**7.81**	10.46	9.48	**11.72**	13.70	12.93	**14.43**	15.95	15.18	**17.15**	19.40	18.42	**21.06**	24.36
Fencing	NA	**NA**	NA	NA	**NA**	NA	NA	**NA**	NA	NA	**NA**	NA	NA	**NA**	NA
Gymnastics	NA	**NA**	NA	NA	**NA**	NA	NA	**NA**	NA	NA	**NA**	NA	NA	**NA**	NA
Handball	2.20	**7.22**	11.14	9.07	**12.44**	15.36	13.83	**16.07**	18.30	16.77	**19.69**	23.07	20.99	**24.91**	29.93
Hockey Rink	NA	**NA**	NA	NA	**NA**	NA	NA	**NA**	NA	NA	**NA**	NA	NA	**NA**	NA
Korfball	NA	**NA**	NA	NA	**NA**	NA	NA	**NA**	NA	NA	**NA**	NA	NA	**NA**	NA
Modern Pentathlon	NA	**NA**	NA	NA	**NA**	NA	NA	**NA**	NA	NA	**NA**	NA	NA	**NA**	NA
Motorsport	NA	**NA**	NA	NA	**NA**	NA	NA	**NA**	NA	NA	**NA**	NA	NA	**NA**	NA
Other combat sports	0.81	**6.90**	11.08	6.37	**10.40**	13.65	10.24	**12.84**	15.43	12.02	**15.27**	19.30	14.59	**18.77**	24.86
Rowing	NA	**NA**	NA	NA	**NA**	NA	NA	**NA**	NA	NA	**NA**	NA	NA	**NA**	NA
Rugby	0.97	**7.10**	11.92	9.68	**13.79**	17.36	15.73	**18.44**	21.14	19.51	**23.09**	27.19	24.95	**29.78**	35.90
Sailing	NA	**NA**	NA	NA	**NA**	NA	NA	**NA**	NA	NA	**NA**	NA	NA	**NA**	NA
Soccer	3.17	**6.55**	9.14	7.31	**9.59**	11.56	10.18	**11.71**	13.23	11.86	**13.82**	16.11	14.28	**16.87**	20.25
Surf	NA	**NA**	NA	NA	**NA**	NA	NA	**NA**	NA	NA	**NA**	NA	NA	**NA**	NA
Swimming	2.66	**6.10**	8.80	7.35	**9.67**	11.70	10.61	**12.16**	13.71	12.62	**14.64**	16.97	15.51	**18.21**	21.65
Tennis	0.00	**8.37**	14.76	7.14	**13.45**	18.38	13.05	**16.97**	20.90	15.57	**20.50**	26.81	19.18	**25.57**	35.31
Triathlon	4.82	**7.20**	9.09	8.06	**9.69**	11.12	10.31	**11.42**	12.53	11.73	**13.15**	14.78	13.76	**15.64**	18.02
Volleyball	3.09	**8.30**	12.05	8.33	**11.81**	14.70	11.96	**14.25**	16.54	13.80	**16.68**	20.17	16.45	**20.19**	25.41
Wrestling and Judo	3.49	**6.26**	8.48	7.63	**9.51**	11.17	10.51	**11.77**	13.04	12.38	**14.04**	15.92	15.07	**17.29**	20.06

NA: data not presented for n < 8.

*Whole-body minus the head

Table 12 – Subtotal* fat-free mass (kg) percentiles by sport and sex

Sport	0.05			0.25			Median			0.75			0.95		
	Low	Estimate	High	Low	Estimate	High	Low	Estimate	High	Low	Estimate	High	Low	Estimate	High
**Females**															
Archery and Shooting	NA	**NA**	NA	NA	**NA**	NA	NA	**NA**	NA	NA	**NA**	NA	NA	**NA**	NA
Athletics	34.65	**37.21**	39.81	39.70	**42.25**	44.84	43.21	**45.76**	48.34	46.71	**49.26**	51.85	51.74	**54.31**	56.90
Basketball	34.94	**36.69**	38.44	39.99	**41.73**	43.47	43.50	**45.24**	46.97	47.00	**48.74**	50.48	52.03	**53.78**	55.54
Fencing	NA	**NA**	NA	NA	**NA**	NA	NA	**NA**	NA	NA	**NA**	NA	NA	**NA**	NA
Gymnastics	24.19	**26.17**	30.13	29.25	**31.21**	35.16	32.76	**34.71**	38.66	36.26	**38.22**	42.17	41.29	**43.26**	47.22
Handball	NA	**NA**	NA	NA	**NA**	NA	NA	**NA**	NA	NA	**NA**	NA	NA	**NA**	NA
Hockey Rink	NA	**NA**	NA	NA	**NA**	NA	NA	**NA**	NA	NA	**NA**	NA	NA	**NA**	NA
Korfball	NA	**NA**	NA	NA	**NA**	NA	NA	**NA**	NA	NA	**NA**	NA	NA	**NA**	NA
Modern Pentathlon	NA	**NA**	NA	NA	**NA**	NA	NA	**NA**	NA	NA	**NA**	NA	NA	**NA**	NA
Motorsport	NA	**NA**	NA	NA	**NA**	NA	NA	**NA**	NA	NA	**NA**	NA	NA	**NA**	NA
Other combat sports	NA	**NA**	NA	NA	**NA**	NA	NA	**NA**	NA	NA	**NA**	NA	NA	**NA**	NA
Rowing	NA	**NA**	NA	NA	**NA**	NA	NA	**NA**	NA	NA	**NA**	NA	NA	**NA**	NA
Rugby	NA	**NA**	NA	NA	**NA**	NA	NA	**NA**	NA	NA	**NA**	NA	NA	**NA**	NA
Sailing	NA	**NA**	NA	NA	**NA**	NA	NA	**NA**	NA	NA	**NA**	NA	NA	**NA**	NA
Soccer	NA	**NA**	NA	NA	**NA**	NA	NA	**NA**	NA	NA	**NA**	NA	NA	**NA**	NA
Surf	NA	**NA**	NA	NA	**NA**	NA	NA	**NA**	NA	NA	**NA**	NA	NA	**NA**	NA
Swimming	30.70	**32.88**	35.10	35.75	**37.93**	40.13	39.26	**41.43**	43.62	42.76	**44.93**	47.14	47.79	**49.98**	52.19
Tennis	NA	**NA**	NA	NA	**NA**	NA	NA	**NA**	NA	NA	**NA**	NA	NA	**NA**	NA
Triathlon	28.27	**31.10**	34.78	33.32	**36.14**	39.81	36.83	**39.65**	43.30	40.33	**43.15**	46.82	45.36	**48.19**	51.87
Volleyball	33.92	**36.51**	39.09	38.98	**41.55**	44.12	42.49	**45.05**	47.62	45.98	**48.56**	51.13	51.02	**53.60**	56.18
Wrestling and Judo	29.45	**32.11**	34.76	34.50	**37.15**	39.80	38.01	**40.65**	43.29	41.51	**44.16**	46.81	46.54	**49.20**	51.86
**Males**															
Archery and Shooting	NA	**NA**	NA	NA	**NA**	NA	NA	**NA**	NA	NA	**NA**	NA	NA	**NA**	NA
Athletics	37.17	**49.31**	58.69	48.05	**56.82**	64.46	55.61	**62.04**	68.47	59.63	**67.26**	76.03	65.40	**74.77**	86.91
Basketball	45.40	**51.87**	57.29	55.14	**59.75**	63.94	61.91	**65.23**	68.55	66.52	**70.71**	75.32	73.16	**78.59**	85.06
Fencing	NA	**NA**	NA	NA	**NA**	NA	NA	**NA**	NA	NA	**NA**	NA	NA	**NA**	NA
Gymnastics	NA	**NA**	NA	NA	**NA**	NA	NA	**NA**	NA	NA	**NA**	NA	NA	**NA**	NA
Handball	45.12	**52.37**	58.36	54.99	**60.17**	64.82	61.85	**65.58**	69.32	66.34	**71.00**	76.18	72.81	**78.80**	86.04
Hockey Rink	NA	**NA**	NA	NA	**NA**	NA	NA	**NA**	NA	NA	**NA**	NA	NA	**NA**	NA
Korfball	NA	**NA**	NA	NA	**NA**	NA	NA	**NA**	NA	NA	**NA**	NA	NA	**NA**	NA
Modern Pentathlon	NA	**NA**	NA	NA	**NA**	NA	NA	**NA**	NA	NA	**NA**	NA	NA	**NA**	NA
Motorsport	NA	**NA**	NA	NA	**NA**	NA	NA	**NA**	NA	NA	**NA**	NA	NA	**NA**	NA
Other combat sports	30.27	**43.30**	53.37	42.74	**52.05**	60.15	51.42	**58.14**	64.86	56.13	**64.22**	73.53	62.91	**72.97**	86.01
Rowing	NA	**NA**	NA	NA	**NA**	NA	NA	**NA**	NA	NA	**NA**	NA	NA	**NA**	NA
Rugby	44.79	**52.44**	58.78	55.61	**61.05**	65.96	63.13	**67.04**	70.95	68.12	**73.03**	78.47	75.30	**81.64**	89.29
Sailing	NA	**NA**	NA	NA	**NA**	NA	NA	**NA**	NA	NA	**NA**	NA	NA	**NA**	NA
Soccer	41.02	**48.29**	54.19	50.02	**55.21**	59.83	56.28	**60.01**	63.75	60.20	**64.82**	70.01	65.84	**71.73**	79.00
Surf	NA	**NA**	NA	NA	**NA**	NA	NA	**NA**	NA	NA	**NA**	NA	NA	**NA**	NA
Swimming	37.98	**45.17**	51.08	47.81	**52.93**	57.52	54.65	**58.32**	61.99	59.12	**63.71**	68.82	65.55	**71.47**	78.66
Tennis	27.75	**42.56**	53.92	41.24	**51.84**	61.02	50.62	**58.29**	65.96	55.56	**64.74**	75.34	62.67	**74.02**	88.83
Triathlon	37.59	**43.50**	48.38	45.75	**49.97**	53.76	51.43	**54.46**	57.49	55.16	**58.95**	63.17	60.54	**65.42**	71.33
Volleyball	52.63	**61.82**	69.09	61.99	**68.63**	74.48	68.50	**73.37**	78.23	72.25	**78.10**	84.74	77.64	**84.92**	94.11
Wrestling and Judo	38.56	**45.01**	50.38	48.22	**52.80**	56.93	54.93	**58.21**	61.49	59.49	**63.63**	68.20	66.04	**71.42**	77.86

NA: data not presented for n < 8.

*Whole-body minus the head

Table 13 – Subtotal* lean soft tissue (kg) percentiles by sport and sex

Sport	0.05			0.25			Median			0.75			0.95		
	Low	Estimate	High	Low	Estimate	High	Low	Estimate	High	Low	Estimate	High	Low	Estimate	High
**Females**															
Archery and Shooting	NA	**NA**	NA	NA	**NA**	NA	NA	**NA**	NA	NA	**NA**	NA	NA	**NA**	NA
Athletics	33.41	**35.90**	38.41	38.23	**40.70**	43.19	41.58	**44.03**	46.51	44.90	**47.37**	49.86	49.68	**52.17**	54.68
Basketball	33.42	**35.14**	36.84	38.24	**39.94**	41.61	41.59	**43.28**	44.93	44.91	**46.61**	48.29	49.69	**51.41**	53.11
Fencing	NA	**NA**	NA	NA	**NA**	NA	NA	**NA**	NA	NA	**NA**	NA	NA	**NA**	NA
Gymnastics	23.47	**26.30**	29.18	28.30	**31.10**	33.95	31.65	**34.44**	37.27	34.97	**37.77**	40.62	39.74	**42.57**	45.45
Handball	NA	**NA**	NA	NA	**NA**	NA	NA	**NA**	NA	NA	**NA**	NA	NA	**NA**	NA
Hockey Rink	NA	**NA**	NA	NA	**NA**	NA	NA	**NA**	NA	NA	**NA**	NA	NA	**NA**	NA
Korfball	NA	**NA**	NA	NA	**NA**	NA	NA	**NA**	NA	NA	**NA**	NA	NA	**NA**	NA
Modern Pentathlon	NA	**NA**	NA	NA	**NA**	NA	NA	**NA**	NA	NA	**NA**	NA	NA	**NA**	NA
Motorsport	NA	**NA**	NA	NA	**NA**	NA	NA	**NA**	NA	NA	**NA**	NA	NA	**NA**	NA
Other combat sports	NA	**NA**	NA	NA	**NA**	NA	NA	**NA**	NA	NA	**NA**	NA	NA	**NA**	NA
Rowing	NA	**NA**	NA	NA	**NA**	NA	NA	**NA**	NA	NA	**NA**	NA	NA	**NA**	NA
Rugby	NA	**NA**	NA	NA	**NA**	NA	NA	**NA**	NA	NA	**NA**	NA	NA	**NA**	NA
Sailing	NA	**NA**	NA	NA	**NA**	NA	NA	**NA**	NA	NA	**NA**	NA	NA	**NA**	NA
Soccer	NA	**NA**	NA	NA	**NA**	NA	NA	**NA**	NA	NA	**NA**	NA	NA	**NA**	NA
Surf	NA	**NA**	NA	NA	**NA**	NA	NA	**NA**	NA	NA	**NA**	NA	NA	**NA**	NA
Swimming	29.73	**31.89**	34.02	34.55	**36.69**	38.79	37.90	**40.02**	42.12	41.22	**43.36**	45.47	46.00	**48.16**	50.29
Tennis	NA	**NA**	NA	NA	**NA**	NA	NA	**NA**	NA	NA	**NA**	NA	NA	**NA**	NA
Triathlon	27.73	**30.98**	33.98	32.55	**35.78**	38.76	35.90	**39.12**	42.08	39.22	**42.46**	45.43	44.00	**47.26**	50.25
Volleyball	32.69	**35.20**	37.71	37.52	**40.00**	42.48	40.87	**43.34**	45.80	44.19	**46.67**	49.15	48.97	**51.47**	53.98
Wrestling and Judo	28.39	**30.94**	33.49	33.21	**35.74**	38.27	36.56	**39.07**	41.59	39.88	**42.41**	44.94	44.66	**47.21**	49.76
**Males**															
Archery and Shooting	NA	**NA**	NA	NA	**NA**	NA	NA	**NA**	NA	NA	**NA**	NA	NA	**NA**	NA
Athletics	40.54	**48.91**	55.39	47.87	**53.98**	59.29	52.96	**57.50**	62.00	55.67	**61.02**	67.09	59.57	**66.08**	74.42
Basketball	44.22	**50.03**	54.89	52.91	**57.06**	60.82	58.95	**61.94**	64.93	63.07	**66.83**	70.97	68.99	**73.85**	79.66
Fencing	NA	**NA**	NA	NA	**NA**	NA	NA	**NA**	NA	NA	**NA**	NA	NA	**NA**	NA
Gymnastics	NA	**NA**	NA	NA	**NA**	NA	NA	**NA**	NA	NA	**NA**	NA	NA	**NA**	NA
Handball	44.14	**50.53**	55.81	52.83	**57.39**	61.49	58.86	**62.16**	65.45	62.82	**66.92**	71.49	68.51	**73.78**	80.17
Hockey Rink	NA	**NA**	NA	NA	**NA**	NA	NA	**NA**	NA	NA	**NA**	NA	NA	**NA**	NA
Korfball	NA	**NA**	NA	NA	**NA**	NA	NA	**NA**	NA	NA	**NA**	NA	NA	**NA**	NA
Modern Pentathlon	NA	**NA**	NA	NA	**NA**	NA	NA	**NA**	NA	NA	**NA**	NA	NA	**NA**	NA
Motorsport	NA	**NA**	NA	NA	**NA**	NA	NA	**NA**	NA	NA	**NA**	NA	NA	**NA**	NA
Other combat sports	33.91	**43.42**	50.79	42.86	**49.70**	55.67	49.08	**54.07**	59.06	52.47	**58.44**	65.29	57.35	**64.73**	74.24
Rowing	NA	**NA**	NA	NA	**NA**	NA	NA	**NA**	NA	NA	**NA**	NA	NA	**NA**	NA
Rugby	43.16	**50.23**	56.09	53.11	**58.15**	62.69	60.02	**63.65**	67.27	64.61	**69.15**	74.19	71.21	**77.06**	84.14
Sailing	NA	**NA**	NA	NA	**NA**	NA	NA	**NA**	NA	NA	**NA**	NA	NA	**NA**	NA
Soccer	42.22	**47.82**	52.37	49.04	**53.06**	56.66	53.78	**56.71**	59.64	56.76	**60.36**	64.38	61.05	**65.60**	71.20
Surf	NA	**NA**	NA	NA	**NA**	NA	NA	**NA**	NA	NA	**NA**	NA	NA	**NA**	NA
Swimming	38.90	**44.82**	49.71	46.93	**51.16**	54.97	52.52	**55.57**	58.63	56.17	**59.98**	64.21	61.44	**66.32**	72.25
Tennis	31.42	**42.51**	51.02	41.37	**49.34**	56.27	48.28	**54.10**	59.92	51.92	**58.85**	66.83	57.17	**65.69**	76.77
Triathlon	40.10	**44.28**	47.75	45.76	**48.77**	51.49	49.70	**51.89**	54.09	52.30	**55.02**	58.03	56.03	**59.51**	63.69
Volleyball	49.60	**58.14**	64.89	58.28	**64.44**	69.88	64.31	**68.83**	73.34	67.78	**73.21**	79.38	72.76	**79.52**	88.05
Wrestling and Judo	38.57	**44.06**	48.65	46.74	**50.65**	54.20	52.43	**55.24**	58.06	56.28	**59.83**	63.74	61.83	**66.42**	71.91

NA: data not presented for n < 8.

*Whole-body minus the head

Table 14 – Appendicular* bone mineral content (g) percentiles by sport and sex

Sport	0.05			0.25			Median			0.75			0.95		
	Low	Estimate	High	Low	Estimate	High	Low	Estimate	High	Low	Estimate	High	Low	Estimate	High
**Females**															
**Archery and Shooting**	NA	**NA**	NA	NA	**NA**	NA	NA	**NA**	NA	NA	**NA**	NA	NA	**NA**	NA
**Athletics**	625	**924**	1153	929	**1139**	1320	1139	**1287**	1435	1255	**1436**	1646	1421	**1650**	1950
**Basketball**	613	**875**	1087	972	**1154**	1316	1221	**1348**	1475	1381	**1542**	1725	1610	**1822**	2084
**Fencing**	NA	**NA**	NA	NA	**NA**	NA	NA	**NA**	NA	NA	**NA**	NA	NA	**NA**	NA
**Gymnastics**	307	**648**	901	625	**862**	1064	846	**1012**	1177	959	**1161**	1398	1122	**1376**	1717
**Handball**	NA	**NA**	NA	NA	**NA**	NA	NA	**NA**	NA	NA	**NA**	NA	NA	**NA**	NA
**Hockey Rink**	NA	**NA**	NA	NA	**NA**	NA	NA	**NA**	NA	NA	**NA**	NA	NA	**NA**	NA
**Korfball**	NA	**NA**	NA	NA	**NA**	NA	NA	**NA**	NA	NA	**NA**	NA	NA	**NA**	NA
**Modern Pentathlon**	NA	**NA**	NA	NA	**NA**	NA	NA	**NA**	NA	NA	**NA**	NA	NA	**NA**	NA
**Motorsport**	NA	**NA**	NA	NA	**NA**	NA	NA	**NA**	NA	NA	**NA**	NA	NA	**NA**	NA
**Other combat sports**	NA	**NA**	NA	NA	**NA**	NA	NA	**NA**	NA	NA	**NA**	NA	NA	**NA**	NA
**Rowing**	NA	**NA**	NA	NA	**NA**	NA	NA	**NA**	NA	NA	**NA**	NA	NA	**NA**	NA
**Rugby**	NA	**NA**	NA	NA	**NA**	NA	NA	**NA**	NA	NA	**NA**	NA	NA	**NA**	NA
**Sailing**	NA	**NA**	NA	NA	**NA**	NA	NA	**NA**	NA	NA	**NA**	NA	NA	**NA**	NA
**Soccer**	NA	**NA**	NA	NA	**NA**	NA	NA	**NA**	NA	NA	**NA**	NA	NA	**NA**	NA
**Surf**	NA	**NA**	NA	NA	**NA**	NA	NA	**NA**	NA	NA	**NA**	NA	NA	**NA**	NA
**Swimming**	467	**691**	865	722	**879**	1015	900	**1009**	1118	1004	**1140**	1296	1153	**1328**	1551
**Tennis**	NA	**NA**	NA	NA	**NA**	NA	NA	**NA**	NA	NA	**NA**	NA	NA	**NA**	NA
**Triathlon**	328	**665**	913	623	**859**	1059	828	**994**	1160	929	**1129**	1365	1075	**1324**	1660
**Volleyball**	611	**911**	1140	915	**1125**	1307	1126	**1274**	1423	1242	**1424**	1634	1409	**1638**	1938
**Wrestling and Judo**	503	**811**	1045	810	**1026**	1211	1023	**1175**	1326	1139	**1324**	1540	1305	**1539**	1847
**Males**															
**Archery and Shooting**	NA	**NA**	NA	NA	**NA**	NA	NA	**NA**	NA	NA	**NA**	NA	NA	**NA**	NA
**Athletics**	718	**1116**	1430	1092	**1382**	1637	1352	**1567**	1781	1496	**1752**	2041	1704	**2018**	2415
**Basketball**	997	**1246**	1456	1386	**1563**	1724	1657	**1783**	1910	1843	**2003**	2180	2110	**2320**	2570
**Fencing**	NA	**NA**	NA	NA	**NA**	NA	NA	**NA**	NA	NA	**NA**	NA	NA	**NA**	NA
**Gymnastics**	NA	**NA**	NA	NA	**NA**	NA	NA	**NA**	NA	NA	**NA**	NA	NA	**NA**	NA
**Handball**	1038	**1289**	1498	1393	**1572**	1733	1639	**1768**	1897	1803	**1964**	2143	2038	**2247**	2498
**Hockey Rink**	NA	**NA**	NA	NA	**NA**	NA	NA	**NA**	NA	NA	**NA**	NA	NA	**NA**	NA
**Korfball**	NA	**NA**	NA	NA	**NA**	NA	NA	**NA**	NA	NA	**NA**	NA	NA	**NA**	NA
**Modern Pentathlon**	NA	**NA**	NA	NA	**NA**	NA	NA	**NA**	NA	NA	**NA**	NA	NA	**NA**	NA
**Motorsport**	NA	**NA**	NA	NA	**NA**	NA	NA	**NA**	NA	NA	**NA**	NA	NA	**NA**	NA
**Other combat sports**	604	**972**	1263	971	**1237**	1471	1226	**1421**	1615	1370	**1605**	1870	1578	**1869**	2237
**Rowing**	NA	**NA**	NA	NA	**NA**	NA	NA	**NA**	NA	NA	**NA**	NA	NA	**NA**	NA
**Rugby**	1128	**1361**	1555	1465	**1632**	1781	1699	**1820**	1939	1857	**2008**	2173	2083	**2278**	2510
**Sailing**	NA	**NA**	NA	NA	**NA**	NA	NA	**NA**	NA	NA	**NA**	NA	NA	**NA**	NA
**Soccer**	943	**1166**	1349	1227	**1388**	1532	1425	**1542**	1659	1552	**1696**	1857	1734	**1918**	2141
**Surf**	NA	**NA**	NA	NA	**NA**	NA	NA	**NA**	NA	NA	**NA**	NA	NA	**NA**	NA
**Swimming**	680	**904**	1091	1000	**1159**	1303	1222	**1336**	1450	1369	**1512**	1672	1581	**1767**	1992
**Tennis**	571	**936**	1223	915	**1181**	1414	1155	**1351**	1547	1288	**1521**	1786	1478	**1766**	2131
**Triathlon**	800	**958**	1091	1027	**1140**	1243	1184	**1266**	1349	1290	**1393**	1506	1442	**1574**	1733
**Volleyball**	1075	**1478**	1801	1510	**1800**	2058	1812	**2024**	2237	1991	**2248**	2539	2248	**2571**	2974
**Wrestling and Judo**	926	**1120**	1282	1223	**1361**	1486	1429	**1529**	1628	1571	**1697**	1835	1775	**1938**	2132

NA: data not presented for n < 8.

*(right arm + left arm + right leg + left leg)

Table 15 – Appendicular* fat-mass (kg) percentiles by sport and sex

Sport	0.05			0.25			Median			0.75			0.95		
	Low	Estimate	High	Low	Estimate	High	Low	Estimate	High	Low	Estimate	High	Low	Estimate	High
**Females**															
Archery and Shooting	NA	**NA**	NA	NA	**NA**	NA	NA	**NA**	NA	NA	**NA**	NA	NA	**NA**	NA
Athletics	2.20	**3.75**	5.57	3.53	**5.14**	7.07	4.90	**6.40**	8.34	5.79	**7.96**	11.59	7.35	**10.91**	18.58
Basketball	4.63	**6.22**	7.92	6.60	**8.17**	9.89	8.45	**9.88**	11.55	9.87	**11.94**	14.79	12.33	**15.69**	21.10
Fencing	NA	**NA**	NA	NA	**NA**	NA	NA	**NA**	NA	NA	**NA**	NA	NA	**NA**	NA
Gymnastics	2.35	**4.37**	6.81	3.85	**5.94**	8.53	5.42	**7.36**	9.98	6.34	**9.10**	14.06	7.94	**12.37**	23.02
Handball	NA	**NA**	NA	NA	**NA**	NA	NA	**NA**	NA	NA	**NA**	NA	NA	**NA**	NA
Hockey Rink	NA	**NA**	NA	NA	**NA**	NA	NA	**NA**	NA	NA	**NA**	NA	NA	**NA**	NA
Korfball	NA	**NA**	NA	NA	**NA**	NA	NA	**NA**	NA	NA	**NA**	NA	NA	**NA**	NA
Modern Pentathlon	NA	**NA**	NA	NA	**NA**	NA	NA	**NA**	NA	NA	**NA**	NA	NA	**NA**	NA
Motorsport	NA	**NA**	NA	NA	**NA**	NA	NA	**NA**	NA	NA	**NA**	NA	NA	**NA**	NA
Other combat sports	NA	**NA**	NA	NA	**NA**	NA	NA	**NA**	NA	NA	**NA**	NA	NA	**NA**	NA
Rowing	NA	**NA**	NA	NA	**NA**	NA	NA	**NA**	NA	NA	**NA**	NA	NA	**NA**	NA
Rugby	NA	**NA**	NA	NA	**NA**	NA	NA	**NA**	NA	NA	**NA**	NA	NA	**NA**	NA
Sailing	NA	**NA**	NA	NA	**NA**	NA	NA	**NA**	NA	NA	**NA**	NA	NA	**NA**	NA
Soccer	NA	**NA**	NA	NA	**NA**	NA	NA	**NA**	NA	NA	**NA**	NA	NA	**NA**	NA
Surf	NA	**NA**	NA	NA	**NA**	NA	NA	**NA**	NA	NA	**NA**	NA	NA	**NA**	NA
Swimming	4.19	**5.62**	7.06	5.59	**6.90**	8.28	6.82	**7.95**	9.26	7.63	**9.16**	11.31	8.96	**11.24**	15.07
Tennis	NA	**NA**	NA	NA	**NA**	NA	NA	**NA**	NA	NA	**NA**	NA	NA	**NA**	NA
Triathlon	1.01	**2.75**	5.46	2.17	**4.32**	7.56	3.69	**5.91**	9.47	4.62	**8.09**	16.10	6.40	**12.71**	34.56
Volleyball	3.15	**5.50**	8.31	5.16	**7.64**	10.67	7.28	**9.61**	12.69	8.66	**12.08**	17.89	11.11	**16.79**	29.33
Wrestling and Judo	2.34	**4.32**	6.78	4.00	**6.15**	8.84	5.81	**7.86**	10.63	6.99	**10.05**	15.45	9.12	**14.30**	26.45
**Males**															
Archery and Shooting	NA	**NA**	NA	NA	**NA**	NA	NA	**NA**	NA	NA	**NA**	NA	NA	**NA**	NA
Athletics	1.33	**2.07**	2.99	1.95	**2.74**	3.74	2.56	**3.34**	4.37	2.99	**4.06**	5.71	3.74	**5.39**	8.41
Basketball	2.68	**3.49**	4.38	3.95	**4.80**	5.75	5.17	**5.99**	6.95	6.24	**7.48**	9.10	8.19	**10.29**	13.42
Fencing	NA	**NA**	NA	NA	**NA**	NA	NA	**NA**	NA	NA	**NA**	NA	NA	**NA**	NA
Gymnastics	NA	**NA**	NA	NA	**NA**	NA	NA	**NA**	NA	NA	**NA**	NA	NA	**NA**	NA
Handball	2.35	**3.29**	4.38	3.68	**4.72**	5.93	5.02	**6.06**	7.32	6.20	**7.79**	10.00	8.39	**11.17**	15.64
Hockey Rink	NA	**NA**	NA	NA	**NA**	NA	NA	**NA**	NA	NA	**NA**	NA	NA	**NA**	NA
Korfball	NA	**NA**	NA	NA	**NA**	NA	NA	**NA**	NA	NA	**NA**	NA	NA	**NA**	NA
Modern Pentathlon	NA	**NA**	NA	NA	**NA**	NA	NA	**NA**	NA	NA	**NA**	NA	NA	**NA**	NA
Motorsport	NA	**NA**	NA	NA	**NA**	NA	NA	**NA**	NA	NA	**NA**	NA	NA	**NA**	NA
Other combat sports	1.55	**2.42**	3.50	2.34	**3.29**	4.46	3.12	**4.06**	5.28	3.70	**5.02**	7.04	4.72	**6.81**	10.63
Rowing	NA	**NA**	NA	NA	**NA**	NA	NA	**NA**	NA	NA	**NA**	NA	NA	**NA**	NA
Rugby	2.49	**3.70**	5.17	4.30	**5.75**	7.50	6.28	**7.82**	9.72	8.14	**10.62**	14.20	11.81	**16.51**	24.49
Sailing	NA	**NA**	NA	NA	**NA**	NA	NA	**NA**	NA	NA	**NA**	NA	NA	**NA**	NA
Soccer	1.63	**2.26**	2.97	2.41	**3.07**	3.83	3.16	**3.80**	4.57	3.77	**4.71**	5.99	4.87	**6.40**	8.84
Surf	NA	**NA**	NA	NA	**NA**	NA	NA	**NA**	NA	NA	**NA**	NA	NA	**NA**	NA
Swimming	1.92	**2.55**	3.25	2.80	**3.46**	4.19	3.64	**4.26**	5.00	4.34	**5.26**	6.49	5.60	**7.12**	9.45
Tennis	1.76	**3.03**	4.74	2.84	**4.31**	6.27	3.97	**5.50**	7.60	4.82	**7.01**	10.62	6.37	**9.96**	17.19
Triathlon	1.76	**2.23**	2.72	2.42	**2.88**	3.39	3.02	**3.45**	3.94	3.52	**4.13**	4.92	4.37	**5.35**	6.78
Volleyball	2.37	**3.48**	4.77	3.49	**4.64**	6.03	4.55	**5.68**	7.09	5.36	**6.95**	9.26	6.78	**9.29**	13.60
Wrestling and Judo	1.88	**2.44**	3.05	2.73	**3.32**	3.97	3.55	**4.11**	4.76	4.25	**5.08**	6.17	5.52	**6.91**	8.98

NA: data not presented for n < 8.

*(right arm + left arm + right leg + left leg)

Table 16 – Appendicular* fat mass (%) percentiles by sport and sex

Sport	0.05			0.25			Median			0.75			0.95		
	Low	Estimate	High	Low	Estimate	High	Low	Estimate	High	Low	Estimate	High	Low	Estimate	High
**Females**															
Archery and Shooting	NA	**NA**	NA	NA	**NA**	NA	NA	**NA**	NA	NA	**NA**	NA	NA	**NA**	NA
Athletics	3.44	**12.09**	18.37	12.13	**17.95**	22.82	18.16	**22.03**	25.91	21.25	**26.10**	31.95	25.70	**31.97**	40.63
Basketball	15.53	**21.49**	26.24	23.50	**27.62**	31.24	29.04	**31.88**	34.72	32.52	**36.14**	40.26	37.52	**42.27**	48.23
Fencing	NA	**NA**	NA	NA	**NA**	NA	NA	**NA**	NA	NA	**NA**	NA	NA	**NA**	NA
Gymnastics	11.89	**21.06**	27.61	20.07	**26.34**	31.52	25.75	**30.00**	34.25	28.47	**33.66**	39.93	32.39	**38.94**	48.11
Handball	NA	**NA**	NA	NA	**NA**	NA	NA	**NA**	NA	NA	**NA**	NA	NA	**NA**	NA
Hockey Rink	NA	**NA**	NA	NA	**NA**	NA	NA	**NA**	NA	NA	**NA**	NA	NA	**NA**	NA
Korfball	NA	**NA**	NA	NA	**NA**	NA	NA	**NA**	NA	NA	**NA**	NA	NA	**NA**	NA
Modern Pentathlon	NA	**NA**	NA	NA	**NA**	NA	NA	**NA**	NA	NA	**NA**	NA	NA	**NA**	NA
Motorsport	NA	**NA**	NA	NA	**NA**	NA	NA	**NA**	NA	NA	**NA**	NA	NA	**NA**	NA
Other combat sports	NA	**NA**	NA	NA	**NA**	NA	NA	**NA**	NA	NA	**NA**	NA	NA	**NA**	NA
Rowing	NA	**NA**	NA	NA	**NA**	NA	NA	**NA**	NA	NA	**NA**	NA	NA	**NA**	NA
Rugby	NA	**NA**	NA	NA	**NA**	NA	NA	**NA**	NA	NA	**NA**	NA	NA	**NA**	NA
Sailing	NA	**NA**	NA	NA	**NA**	NA	NA	**NA**	NA	NA	**NA**	NA	NA	**NA**	NA
Soccer	NA	**NA**	NA	NA	**NA**	NA	NA	**NA**	NA	NA	**NA**	NA	NA	**NA**	NA
Surf	NA	**NA**	NA	NA	**NA**	NA	NA	**NA**	NA	NA	**NA**	NA	NA	**NA**	NA
Swimming	16.28	**21.63**	25.73	22.17	**25.87**	29.07	26.26	**28.82**	31.39	28.58	**31.77**	35.48	31.91	**36.01**	41.36
Tennis	NA	**NA**	NA	NA	**NA**	NA	NA	**NA**	NA	NA	**NA**	NA	NA	**NA**	NA
Triathlon	0.00	**11.59**	20.96	9.76	**18.93**	26.23	18.16	**24.04**	29.90	21.82	**29.14**	38.30	27.10	**36.48**	50.38
Volleyball	12.41	**20.98**	27.25	20.86	**26.72**	31.63	26.74	**30.72**	34.68	29.78	**34.71**	40.55	34.16	**40.46**	49.00
Wrestling and Judo	5.97	**17.20**	25.32	16.95	**24.54**	30.86	24.58	**29.65**	34.72	28.44	**34.76**	42.35	33.98	**42.11**	53.33
**Males**															
Archery and Shooting	NA	**NA**	NA	NA	**NA**	NA	NA	**NA**	NA	NA	**NA**	NA	NA	**NA**	NA
Athletics	0.00	**4.46**	9.03	3.79	**8.18**	11.74	7.89	**10.76**	13.63	9.78	**13.34**	17.73	12.49	**17.05**	23.64
Basketball	5.84	**9.25**	12.02	11.04	**13.37**	15.45	14.65	**16.24**	17.84	17.03	**19.11**	21.45	20.46	**23.24**	26.65
Fencing	NA	**NA**	NA	NA	**NA**	NA	NA	**NA**	NA	NA	**NA**	NA	NA	**NA**	NA
Gymnastics	NA	**NA**	NA	NA	**NA**	NA	NA	**NA**	NA	NA	**NA**	NA	NA	**NA**	NA
Handball	3.05	**7.91**	11.76	9.78	**13.08**	15.97	14.46	**16.68**	18.90	17.39	**20.28**	23.58	21.61	**25.45**	30.31
Hockey Rink	NA	**NA**	NA	NA	**NA**	NA	NA	**NA**	NA	NA	**NA**	NA	NA	**NA**	NA
Korfball	NA	**NA**	NA	NA	**NA**	NA	NA	**NA**	NA	NA	**NA**	NA	NA	**NA**	NA
Modern Pentathlon	NA	**NA**	NA	NA	**NA**	NA	NA	**NA**	NA	NA	**NA**	NA	NA	**NA**	NA
Motorsport	NA	**NA**	NA	NA	**NA**	NA	NA	**NA**	NA	NA	**NA**	NA	NA	**NA**	NA
Other combat sports	1.29	**7.36**	11.69	7.00	**11.09**	14.47	10.96	**13.68**	16.40	12.89	**16.27**	20.36	15.67	**20.00**	26.07
Rowing	NA	**NA**	NA	NA	**NA**	NA	NA	**NA**	NA	NA	**NA**	NA	NA	**NA**	NA
Rugby	3.46	**9.32**	14.01	11.88	**15.86**	19.36	17.73	**20.40**	23.08	21.45	**24.94**	28.94	26.80	**31.48**	37.36
Sailing	NA	**NA**	NA	NA	**NA**	NA	NA	**NA**	NA	NA	**NA**	NA	NA	**NA**	NA
Soccer	2.13	**5.98**	8.96	6.95	**9.56**	11.82	10.30	**12.05**	13.80	12.28	**14.53**	17.15	15.14	**18.11**	21.97
Surf	NA	**NA**	NA	NA	**NA**	NA	NA	**NA**	NA	NA	**NA**	NA	NA	**NA**	NA
Swimming	4.00	**7.53**	10.34	8.87	**11.28**	13.39	12.25	**13.88**	15.52	14.38	**16.49**	18.90	17.43	**20.23**	23.76
Tennis	0.72	**9.49**	15.63	8.52	**14.42**	19.26	13.94	**17.86**	21.78	16.46	**21.29**	27.20	20.08	**26.23**	35.00
Triathlon	4.00	**6.83**	9.10	7.98	**9.93**	11.64	10.75	**12.08**	13.40	12.51	**14.23**	16.17	15.05	**17.32**	20.15
Volleyball	2.43	**7.84**	11.83	8.01	**11.68**	14.76	11.89	**14.35**	16.80	13.93	**17.01**	20.68	16.86	**20.85**	26.27
Wrestling and Judo	3.71	**6.90**	9.47	8.54	**10.71**	12.64	11.89	**13.36**	14.84	14.09	**16.01**	18.19	17.25	**19.83**	23.01

NA: data not presented for n < 8.

*(right arm + left arm + right leg + left leg)

Table 17 – Appendicular* fat-free mass (kg) percentiles by sport and sex

Sport	0.05			0.25			Median			0.75			0.95		
	Low	Estimate	High	Low	Estimate	High	Low	Estimate	High	Low	Estimate	High	Low	Estimate	High
**Females**															
Archery and Shooting	NA	**NA**	NA	NA	**NA**	NA	NA	**NA**	NA	NA	**NA**	NA	NA	**NA**	NA
Athletics	14.62	**18.40**	21.47	18.62	**21.40**	23.88	21.41	**23.48**	25.55	23.08	**25.56**	28.34	25.49	**28.56**	32.34
Basketball	14.70	**17.06**	19.03	17.97	**19.68**	21.23	20.25	**21.51**	22.76	21.78	**23.33**	25.04	23.98	**25.95**	28.31
Fencing	NA	**NA**	NA	NA	**NA**	NA	NA	**NA**	NA	NA	**NA**	NA	NA	**NA**	NA
Gymnastics	9.91	**13.26**	15.99	13.17	**15.65**	17.88	15.44	**17.31**	19.20	16.75	**18.97**	21.47	18.65	**21.36**	24.73
Handball	NA	**NA**	NA	NA	**NA**	NA	NA	**NA**	NA	NA	**NA**	NA	NA	**NA**	NA
Hockey Rink	NA	**NA**	NA	NA	**NA**	NA	NA	**NA**	NA	NA	**NA**	NA	NA	**NA**	NA
Korfball	NA	**NA**	NA	NA	**NA**	NA	NA	**NA**	NA	NA	**NA**	NA	NA	**NA**	NA
Modern Pentathlon	NA	**NA**	NA	NA	**NA**	NA	NA	**NA**	NA	NA	**NA**	NA	NA	**NA**	NA
Motorsport	NA	**NA**	NA	NA	**NA**	NA	NA	**NA**	NA	NA	**NA**	NA	NA	**NA**	NA
Other combat sports	NA	**NA**	NA	NA	**NA**	NA	NA	**NA**	NA	NA	**NA**	NA	NA	**NA**	NA
Rowing	NA	**NA**	NA	NA	**NA**	NA	NA	**NA**	NA	NA	**NA**	NA	NA	**NA**	NA
Rugby	NA	**NA**	NA	NA	**NA**	NA	NA	**NA**	NA	NA	**NA**	NA	NA	**NA**	NA
Sailing	NA	**NA**	NA	NA	**NA**	NA	NA	**NA**	NA	NA	**NA**	NA	NA	**NA**	NA
Soccer	NA	**NA**	NA	NA	**NA**	NA	NA	**NA**	NA	NA	**NA**	NA	NA	**NA**	NA
Surf	NA	**NA**	NA	NA	**NA**	NA	NA	**NA**	NA	NA	**NA**	NA	NA	**NA**	NA
Swimming	13.10	**15.74**	17.91	16.20	**18.13**	19.86	18.36	**19.79**	21.22	19.71	**21.44**	23.37	21.66	**23.83**	26.47
Tennis	NA	**NA**	NA	NA	**NA**	NA	NA	**NA**	NA	NA	**NA**	NA	NA	**NA**	NA
Triathlon	12.26	**15.80**	18.67	15.47	**18.12**	20.49	17.70	**19.73**	21.77	18.97	**21.35**	24.00	20.80	**23.67**	27.21
Volleyball	12.89	**16.82**	20.02	17.08	**19.96**	22.54	19.99	**22.14**	24.29	21.74	**24.32**	27.20	24.26	**27.46**	31.39
Wrestling and Judo	10.51	**14.29**	17.36	14.47	**17.24**	19.72	17.23	**19.30**	21.36	18.87	**21.35**	24.12	21.23	**24.30**	28.09
**Males**															
Archery and Shooting	NA	**NA**	NA	NA	**NA**	NA	NA	**NA**	NA	NA	**NA**	NA	NA	**NA**	NA
Athletics	21.01	**25.94**	29.78	25.39	**28.98**	32.13	28.44	**31.10**	33.76	30.07	**33.22**	36.81	32.42	**36.26**	41.19
Basketball	22.64	**25.94**	28.71	27.63	**29.98**	32.11	31.09	**32.78**	34.48	33.46	**35.59**	37.94	36.86	**39.63**	42.93
Fencing	NA	**NA**	NA	NA	**NA**	NA	NA	**NA**	NA	NA	**NA**	NA	NA	**NA**	NA
Gymnastics	NA	**NA**	NA	NA	**NA**	NA	NA	**NA**	NA	NA	**NA**	NA	NA	**NA**	NA
Handball	22.43	**26.11**	29.15	27.47	**30.09**	32.46	30.97	**32.86**	34.75	33.26	**35.63**	38.25	36.57	**39.61**	43.29
Hockey Rink	NA	**NA**	NA	NA	**NA**	NA	NA	**NA**	NA	NA	**NA**	NA	NA	**NA**	NA
Korfball	NA	**NA**	NA	NA	**NA**	NA	NA	**NA**	NA	NA	**NA**	NA	NA	**NA**	NA
Modern Pentathlon	NA	**NA**	NA	NA	**NA**	NA	NA	**NA**	NA	NA	**NA**	NA	NA	**NA**	NA
Motorsport	NA	**NA**	NA	NA	**NA**	NA	NA	**NA**	NA	NA	**NA**	NA	NA	**NA**	NA
Other combat sports	17.19	**22.41**	26.47	22.15	**25.91**	29.19	25.59	**28.34**	31.08	27.48	**30.77**	34.53	30.20	**34.27**	39.49
Rowing	NA	**NA**	NA	NA	**NA**	NA	NA	**NA**	NA	NA	**NA**	NA	NA	**NA**	NA
Rugby	22.18	**25.96**	29.09	27.53	**30.21**	32.64	31.24	**33.17**	35.11	33.71	**36.13**	38.82	37.26	**40.39**	44.16
Sailing	NA	**NA**	NA	NA	**NA**	NA	NA	**NA**	NA	NA	**NA**	NA	NA	**NA**	NA
Soccer	21.77	**24.87**	27.40	25.58	**27.81**	29.80	28.23	**29.85**	31.47	29.90	**31.89**	34.12	32.30	**34.82**	37.93
Surf	NA	**NA**	NA	NA	**NA**	NA	NA	**NA**	NA	NA	**NA**	NA	NA	**NA**	NA
Swimming	19.13	**22.33**	24.97	23.51	**25.79**	27.85	26.56	**28.20**	29.85	28.56	**30.61**	32.89	31.44	**34.08**	37.28
Tennis	15.77	**22.03**	26.87	21.45	**25.96**	29.89	25.40	**28.70**	31.99	27.50	**31.43**	35.94	30.52	**35.36**	41.62
Triathlon	19.38	**21.87**	23.95	22.84	**24.63**	26.24	25.24	**26.54**	27.84	26.83	**28.45**	30.24	29.13	**31.20**	33.70
Volleyball	25.78	**30.70**	34.59	30.83	**34.38**	37.51	34.35	**36.94**	39.54	36.37	**39.50**	43.05	39.29	**43.19**	48.11
Wrestling and Judo	19.07	**22.10**	24.64	23.61	**25.77**	27.73	26.76	**28.32**	29.87	28.90	**30.86**	33.03	31.99	**34.53**	37.56

NA: data not presented for n < 8.

*(right arm + left arm + right leg + left leg)

Table 18 – Appendicular* lean soft tissue (kg) percentiles by sport and sex

Sport	0.05			0.25			Median			0.75			0.95		
	Low	Estimate	High	Low	Estimate	High	Low	Estimate	High	Low	Estimate	High	Low	Estimate	High
**Females**															
Archery and Shooting	NA	**NA**	NA	NA	**NA**	NA	NA	**NA**	NA	NA	**NA**	NA	NA	**NA**	NA
Athletics	14.01	**17.46**	20.31	17.72	**20.27**	22.58	20.29	**22.23**	24.16	21.87	**24.18**	26.73	24.15	**26.99**	30.44
Basketball	13.77	**15.97**	17.81	16.85	**18.45**	19.90	19.00	**20.17**	21.35	20.45	**21.90**	23.49	22.53	**24.38**	26.57
Fencing	NA	**NA**	NA	NA	**NA**	NA	NA	**NA**	NA	NA	**NA**	NA	NA	**NA**	NA
Gymnastics	9.31	**12.45**	15.03	12.41	**14.75**	16.86	14.56	**16.34**	18.14	15.84	**17.94**	20.29	17.67	**20.24**	23.39
Handball	NA	**NA**	NA	NA	**NA**	NA	NA	**NA**	NA	NA	**NA**	NA	NA	**NA**	NA
Hockey Rink	NA	**NA**	NA	NA	**NA**	NA	NA	**NA**	NA	NA	**NA**	NA	NA	**NA**	NA
Korfball	NA	**NA**	NA	NA	**NA**	NA	NA	**NA**	NA	NA	**NA**	NA	NA	**NA**	NA
Modern Pentathlon	NA	**NA**	NA	NA	**NA**	NA	NA	**NA**	NA	NA	**NA**	NA	NA	**NA**	NA
Motorsport	NA	**NA**	NA	NA	**NA**	NA	NA	**NA**	NA	NA	**NA**	NA	NA	**NA**	NA
Other combat sports	NA	**NA**	NA	NA	**NA**	NA	NA	**NA**	NA	NA	**NA**	NA	NA	**NA**	NA
Rowing	NA	**NA**	NA	NA	**NA**	NA	NA	**NA**	NA	NA	**NA**	NA	NA	**NA**	NA
Rugby	NA	**NA**	NA	NA	**NA**	NA	NA	**NA**	NA	NA	**NA**	NA	NA	**NA**	NA
Sailing	NA	**NA**	NA	NA	**NA**	NA	NA	**NA**	NA	NA	**NA**	NA	NA	**NA**	NA
Soccer	NA	**NA**	NA	NA	**NA**	NA	NA	**NA**	NA	NA	**NA**	NA	NA	**NA**	NA
Surf	NA	**NA**	NA	NA	**NA**	NA	NA	**NA**	NA	NA	**NA**	NA	NA	**NA**	NA
Swimming	12.44	**14.91**	16.97	15.39	**17.21**	18.85	17.44	**18.80**	20.16	18.75	**20.39**	22.21	20.63	**22.69**	25.17
Tennis	NA	**NA**	NA	NA	**NA**	NA	NA	**NA**	NA	NA	**NA**	NA	NA	**NA**	NA
Triathlon	11.68	**14.99**	17.71	14.73	**17.23**	19.49	16.86	**18.79**	20.72	18.09	**20.35**	22.85	19.87	**22.59**	25.90
Volleyball	12.30	**15.90**	18.86	16.19	**18.85**	21.24	18.90	**20.90**	22.89	20.55	**22.94**	25.60	22.93	**25.89**	29.50
Wrestling and Judo	9.92	**13.42**	16.27	13.63	**16.21**	18.52	16.22	**18.16**	20.09	17.78	**20.10**	22.67	20.03	**22.90**	26.38
**Males**															
Archery and Shooting	NA	**NA**	NA	NA	**NA**	NA	NA	**NA**	NA	NA	**NA**	NA	NA	**NA**	NA
Athletics	19.88	**24.57**	28.21	24.04	**27.45**	30.43	26.93	**29.46**	31.98	28.48	**31.46**	34.87	30.70	**34.34**	39.03
Basketball	21.40	**24.52**	27.14	26.11	**28.33**	30.35	29.38	**30.98**	32.58	31.61	**33.63**	35.85	34.83	**37.44**	40.56
Fencing	NA	**NA**	NA	NA	**NA**	NA	NA	**NA**	NA	NA	**NA**	NA	NA	**NA**	NA
Gymnastics	NA	**NA**	NA	NA	**NA**	NA	NA	**NA**	NA	NA	**NA**	NA	NA	**NA**	NA
Handball	21.21	**24.69**	27.57	25.97	**28.45**	30.69	29.28	**31.07**	32.86	31.45	**33.68**	36.17	34.57	**37.44**	40.93
Hockey Rink	NA	**NA**	NA	NA	**NA**	NA	NA	**NA**	NA	NA	**NA**	NA	NA	**NA**	NA
Korfball	NA	**NA**	NA	NA	**NA**	NA	NA	**NA**	NA	NA	**NA**	NA	NA	**NA**	NA
Modern Pentathlon	NA	**NA**	NA	NA	**NA**	NA	NA	**NA**	NA	NA	**NA**	NA	NA	**NA**	NA
Motorsport	NA	**NA**	NA	NA	**NA**	NA	NA	**NA**	NA	NA	**NA**	NA	NA	**NA**	NA
Other combat sports	16.37	**21.29**	25.11	21.03	**24.57**	27.66	24.26	**26.85**	29.43	26.04	**29.13**	32.67	28.59	**32.41**	37.33
Rowing	NA	**NA**	NA	NA	**NA**	NA	NA	**NA**	NA	NA	**NA**	NA	NA	**NA**	NA
Rugby	20.80	**24.42**	27.42	25.92	**28.50**	30.81	29.48	**31.33**	33.18	31.84	**34.16**	36.73	35.24	**38.24**	41.85
Sailing	NA	**NA**	NA	NA	**NA**	NA	NA	**NA**	NA	NA	**NA**	NA	NA	**NA**	NA
Soccer	20.62	**23.56**	25.95	24.23	**26.34**	28.23	26.74	**28.27**	29.81	28.32	**30.21**	32.32	30.59	**32.99**	35.93
Surf	NA	**NA**	NA	NA	**NA**	NA	NA	**NA**	NA	NA	**NA**	NA	NA	**NA**	NA
Swimming	18.28	**21.30**	23.79	22.42	**24.57**	26.50	25.29	**26.84**	28.39	27.18	**29.11**	31.26	29.89	**32.38**	35.40
Tennis	14.86	**20.89**	25.52	20.31	**24.65**	28.41	24.10	**27.27**	30.42	26.10	**29.88**	34.21	28.99	**33.64**	39.66
Triathlon	18.46	**20.84**	22.81	21.74	**23.44**	24.98	24.01	**25.25**	26.48	25.52	**27.06**	28.76	27.69	**29.66**	32.03
Volleyball	24.28	**28.96**	32.66	29.07	**32.44**	35.42	32.39	**34.87**	37.34	34.31	**37.29**	40.66	37.07	**40.77**	45.45
Wrestling and Judo	17.96	**20.84**	23.25	22.29	**24.34**	26.19	25.30	**26.77**	28.24	27.34	**29.20**	31.25	30.28	**32.69**	35.57

NA: data not presented for n < 8.

*(right arm + left arm + right leg + left leg)

Table 19 – Appendicular* lean soft tissue index (kg/m^2) percentiles by sport and sex

Sport	0.05			0.25			Median			0.75			0.95		
	Low	Estimate	High	Low	Estimate	High	Low	Estimate	High	Low	Estimate	High	Low	Estimate	High
**Females**															
**Archery and Shooting**	NA	**NA**	NA	NA	**NA**	NA	NA	**NA**	NA	NA	**NA**	NA	NA	**NA**	NA
**Athletics**	5.32	**6.25**	7.13	6.25	**7.05**	7.85	6.99	**7.66**	8.39	7.47	**8.33**	9.39	8.23	**9.39**	11.03
**Basketball**	4.91	**5.44**	5.94	5.62	**6.06**	6.49	6.17	**6.53**	6.91	6.57	**7.04**	7.59	7.19	**7.84**	8.69
**Fencing**	NA	**NA**	NA	NA	**NA**	NA	NA	**NA**	NA	NA	**NA**	NA	NA	**NA**	NA
**Gymnastics**	4.50	**5.21**	5.87	5.14	**5.74**	6.34	5.64	**6.14**	6.69	5.95	**6.58**	7.34	6.43	**7.25**	8.40
**Handball**	NA	**NA**	NA	NA	**NA**	NA	NA	**NA**	NA	NA	**NA**	NA	NA	**NA**	NA
**Hockey Rink**	NA	**NA**	NA	NA	**NA**	NA	NA	**NA**	NA	NA	**NA**	NA	NA	**NA**	NA
**Korfball**	NA	**NA**	NA	NA	**NA**	NA	NA	**NA**	NA	NA	**NA**	NA	NA	**NA**	NA
**Modern Pentathlon**	NA	**NA**	NA	NA	**NA**	NA	NA	**NA**	NA	NA	**NA**	NA	NA	**NA**	NA
**Motorsport**	NA	**NA**	NA	NA	**NA**	NA	NA	**NA**	NA	NA	**NA**	NA	NA	**NA**	NA
**Other combat sports**	NA	**NA**	NA	NA	**NA**	NA	NA	**NA**	NA	NA	**NA**	NA	NA	**NA**	NA
**Rowing**	NA	**NA**	NA	NA	**NA**	NA	NA	**NA**	NA	NA	**NA**	NA	NA	**NA**	NA
**Rugby**	NA	**NA**	NA	NA	**NA**	NA	NA	**NA**	NA	NA	**NA**	NA	NA	**NA**	NA
**Sailing**	NA	**NA**	NA	NA	**NA**	NA	NA	**NA**	NA	NA	**NA**	NA	NA	**NA**	NA
**Soccer**	NA	**NA**	NA	NA	**NA**	NA	NA	**NA**	NA	NA	**NA**	NA	NA	**NA**	NA
**Surf**	NA	**NA**	NA	NA	**NA**	NA	NA	**NA**	NA	NA	**NA**	NA	NA	**NA**	NA
**Swimming**	5.11	**5.69**	6.22	5.75	**6.23**	6.71	6.25	**6.64**	7.06	6.58	**7.08**	7.67	7.09	**7.75**	8.64
**Tennis**	NA	**NA**	NA	NA	**NA**	NA	NA	**NA**	NA	NA	**NA**	NA	NA	**NA**	NA
**Triathlon**	4.54	**5.43**	6.28	5.30	**6.07**	6.85	5.90	**6.55**	7.28	6.27	**7.08**	8.10	6.84	**7.91**	9.46
**Volleyball**	4.77	**5.54**	6.26	5.55	**6.20**	6.85	6.16	**6.70**	7.29	6.56	**7.24**	8.09	7.17	**8.10**	9.40
**Wrestling and Judo**	4.48	**5.34**	6.16	5.33	**6.07**	6.82	6.01	**6.64**	7.33	6.46	**7.26**	8.26	7.15	**8.25**	9.83
**Males**															
**Archery and Shooting**	NA	**NA**	NA	NA	**NA**	NA	NA	**NA**	NA	NA	**NA**	NA	NA	**NA**	NA
**Athletics**	6.22	**7.43**	8.65	7.34	**8.44**	9.59	8.24	**9.21**	10.30	8.85	**10.06**	11.56	9.82	**11.42**	13.64
**Basketball**	6.47	**7.07**	7.64	7.40	**7.91**	8.42	8.13	**8.56**	9.00	8.70	**9.25**	9.89	9.59	**10.36**	11.32
**Fencing**	NA	**NA**	NA	NA	**NA**	NA	NA	**NA**	NA	NA	**NA**	NA	NA	**NA**	NA
**Gymnastics**	NA	**NA**	NA	NA	**NA**	NA	NA	**NA**	NA	NA	**NA**	NA	NA	**NA**	NA
**Handball**	6.97	**7.70**	8.38	7.99	**8.61**	9.23	8.79	**9.31**	9.86	9.40	**10.07**	10.85	10.35	**11.27**	12.44
**Hockey Rink**	NA	**NA**	NA	NA	**NA**	NA	NA	**NA**	NA	NA	**NA**	NA	NA	**NA**	NA
**Korfball**	NA	**NA**	NA	NA	**NA**	NA	NA	**NA**	NA	NA	**NA**	NA	NA	**NA**	NA
**Modern Pentathlon**	NA	**NA**	NA	NA	**NA**	NA	NA	**NA**	NA	NA	**NA**	NA	NA	**NA**	NA
**Motorsport**	NA	**NA**	NA	NA	**NA**	NA	NA	**NA**	NA	NA	**NA**	NA	NA	**NA**	NA
**Other combat sports**	5.94	**7.04**	8.13	7.02	**8.00**	9.03	7.88	**8.75**	9.72	8.48	**9.57**	10.91	9.42	**10.88**	12.89
**Rowing**	NA	**NA**	NA	NA	**NA**	NA	NA	**NA**	NA	NA	**NA**	NA	NA	**NA**	NA
**Rugby**	6.94	**7.72**	8.47	8.09	**8.76**	9.43	9.00	**9.57**	10.17	9.70	**10.45**	11.31	10.81	**11.86**	13.19
**Sailing**	NA	**NA**	NA	NA	**NA**	NA	NA	**NA**	NA	NA	**NA**	NA	NA	**NA**	NA
**Soccer**	6.85	**7.64**	8.40	7.86	**8.54**	9.22	8.65	**9.23**	9.84	9.24	**9.97**	10.83	10.14	**11.15**	12.43
**Surf**	NA	**NA**	NA	NA	**NA**	NA	NA	**NA**	NA	NA	**NA**	NA	NA	**NA**	NA
**Swimming**	6.27	**6.93**	7.56	7.21	**7.78**	8.33	7.95	**8.42**	8.92	8.51	**9.12**	9.83	9.38	**10.22**	11.30
**Tennis**	5.93	**7.13**	8.34	7.05	**8.14**	9.30	7.95	**8.93**	10.02	8.57	**9.79**	11.30	9.55	**11.18**	13.44
**Triathlon**	6.16	**6.79**	7.37	7.06	**7.59**	8.11	7.76	**8.20**	8.67	8.29	**8.86**	9.52	9.12	**9.91**	10.91
**Volleyball**	6.76	**7.76**	8.73	7.83	**8.71**	9.61	8.67	**9.44**	10.27	9.27	**10.23**	11.38	10.20	**11.48**	13.19
**Wrestling and Judo**	6.68	**7.36**	8.00	7.73	**8.31**	8.88	8.55	**9.04**	9.55	9.19	**9.83**	10.56	10.20	**11.10**	12.22

NA: data not presented for n < 8.

*(right arm + left arm + right leg + left leg)

Table 20 – Trunk bone mineral content (g) percentiles by sport and sex

Sport	0.05			0.25			Median			0.75			0.95		
	Low	Estimate	High	Low	Estimate	High	Low	Estimate	High	Low	Estimate	High	Low	Estimate	High
**Females**															
**Archery and Shooting**	NA	**NA**	NA	NA	**NA**	NA	NA	**NA**	NA	NA	**NA**	NA	NA	**NA**	NA
**Athletics**	190	**395**	554	407	**551**	676	557	**659**	761	642	**767**	911	764	**923**	1128
**Basketball**	267	**426**	555	490	**600**	699	644	**721**	799	744	**843**	953	888	**1017**	1176
**Fencing**	NA	**NA**	NA	NA	**NA**	NA	NA	**NA**	NA	NA	**NA**	NA	NA	**NA**	NA
**Gymnastics**	89	**301**	463	294	**443**	572	437	**542**	648	513	**641**	790	622	**783**	995
**Handball**	NA	**NA**	NA	NA	**NA**	NA	NA	**NA**	NA	NA	**NA**	NA	NA	**NA**	NA
**Hockey Rink**	NA	**NA**	NA	NA	**NA**	NA	NA	**NA**	NA	NA	**NA**	NA	NA	**NA**	NA
**Korfball**	NA	**NA**	NA	NA	**NA**	NA	NA	**NA**	NA	NA	**NA**	NA	NA	**NA**	NA
**Modern Pentathlon**	NA	**NA**	NA	NA	**NA**	NA	NA	**NA**	NA	NA	**NA**	NA	NA	**NA**	NA
**Motorsport**	NA	**NA**	NA	NA	**NA**	NA	NA	**NA**	NA	NA	**NA**	NA	NA	**NA**	NA
**Other combat sports**	NA	**NA**	NA	NA	**NA**	NA	NA	**NA**	NA	NA	**NA**	NA	NA	**NA**	NA
**Rowing**	NA	**NA**	NA	NA	**NA**	NA	NA	**NA**	NA	NA	**NA**	NA	NA	**NA**	NA
**Rugby**	NA	**NA**	NA	NA	**NA**	NA	NA	**NA**	NA	NA	**NA**	NA	NA	**NA**	NA
**Sailing**	NA	**NA**	NA	NA	**NA**	NA	NA	**NA**	NA	NA	**NA**	NA	NA	**NA**	NA
**Soccer**	NA	**NA**	NA	NA	**NA**	NA	NA	**NA**	NA	NA	**NA**	NA	NA	**NA**	NA
**Surf**	NA	**NA**	NA	NA	**NA**	NA	NA	**NA**	NA	NA	**NA**	NA	NA	**NA**	NA
**Swimming**	236	**372**	480	395	**491**	575	506	**574**	642	573	**657**	752	668	**776**	912
**Tennis**	NA	**NA**	NA	NA	**NA**	NA	NA	**NA**	NA	NA	**NA**	NA	NA	**NA**	NA
**Triathlon**	53	**244**	389	228	**363**	479	349	**445**	541	412	**528**	663	502	**647**	838
**Volleyball**	218	**416**	569	426	**566**	687	571	**670**	768	653	**774**	913	770	**924**	1122
**Wrestling and Judo**	178	**389**	551	396	**544**	672	548	**652**	757	632	**760**	908	753	**915**	1126
**Males**															
**Archery and Shooting**	NA	**NA**	NA	NA	**NA**	NA	NA	**NA**	NA	NA	**NA**	NA	NA	**NA**	NA
**Athletics**	407	**602**	757	590	**733**	859	717	**823**	929	788	**914**	1057	890	**1044**	1239
**Basketball**	581	**700**	800	766	**851**	928	894	**955**	1016	983	**1060**	1144	1110	**1210**	1329
**Fencing**	NA	**NA**	NA	NA	**NA**	NA	NA	**NA**	NA	NA	**NA**	NA	NA	**NA**	NA
**Gymnastics**	NA	**NA**	NA	NA	**NA**	NA	NA	**NA**	NA	NA	**NA**	NA	NA	**NA**	NA
**Handball**	587	**716**	824	769	**861**	944	895	**961**	1028	979	**1062**	1155	1100	**1207**	1336
**Hockey Rink**	NA	**NA**	NA	NA	**NA**	NA	NA	**NA**	NA	NA	**NA**	NA	NA	**NA**	NA
**Korfball**	NA	**NA**	NA	NA	**NA**	NA	NA	**NA**	NA	NA	**NA**	NA	NA	**NA**	NA
**Modern Pentathlon**	NA	**NA**	NA	NA	**NA**	NA	NA	**NA**	NA	NA	**NA**	NA	NA	**NA**	NA
**Motorsport**	NA	**NA**	NA	NA	**NA**	NA	NA	**NA**	NA	NA	**NA**	NA	NA	**NA**	NA
**Other combat sports**	406	**583**	723	581	**709**	822	702	**797**	891	771	**884**	1013	870	**1010**	1187
**Rowing**	NA	**NA**	NA	NA	**NA**	NA	NA	**NA**	NA	NA	**NA**	NA	NA	**NA**	NA
**Rugby**	582	**698**	794	749	**832**	906	866	**925**	984	944	**1018**	1101	1056	**1152**	1268
**Sailing**	NA	**NA**	NA	NA	**NA**	NA	NA	**NA**	NA	NA	**NA**	NA	NA	**NA**	NA
**Soccer**	553	**672**	769	704	**789**	866	809	**871**	934	876	**953**	1039	973	**1071**	1190
**Surf**	NA	**NA**	NA	NA	**NA**	NA	NA	**NA**	NA	NA	**NA**	NA	NA	**NA**	NA
**Swimming**	361	**482**	582	533	**618**	695	652	**713**	774	731	**808**	894	845	**945**	1065
**Tennis**	321	**494**	631	484	**610**	721	597	**690**	784	660	**771**	897	750	**887**	1060
**Triathlon**	372	**455**	523	491	**550**	603	573	**615**	658	628	**681**	740	707	**776**	859
**Volleyball**	642	**829**	979	842	**977**	1098	981	**1081**	1180	1064	**1184**	1320	1183	**1333**	1520
**Wrestling and Judo**	543	**651**	742	709	**786**	856	825	**880**	936	904	**974**	1052	1019	**1110**	1218

NA: data not presented for n < 8.

Table 21 – Trunk fat mass (kg) percentiles by sport and sex

Sport	0.05			0.25			Median			0.75			0.95		
	Low	Estimate	High	Low	Estimate	High	Low	Estimate	High	Low	Estimate	High	Low	Estimate	High
**Females**															
Archery and Shooting	NA	**NA**	NA	NA	**NA**	NA	NA	**NA**	NA	NA	**NA**	NA	NA	**NA**	NA
Athletics	1.74	**2.55**	3.44	2.48	**3.28**	4.18	3.17	**3.90**	4.79	3.63	**4.64**	6.13	4.42	**5.96**	8.73
Basketball	2.90	**3.79**	4.75	4.04	**4.93**	5.89	5.10	**5.91**	6.85	5.92	**7.09**	8.64	7.35	**9.20**	12.06
Fencing	NA	**NA**	NA	NA	**NA**	NA	NA	**NA**	NA	NA	**NA**	NA	NA	**NA**	NA
Gymnastics	1.02	**1.95**	3.21	1.77	**2.84**	4.28	2.61	**3.69**	5.22	3.18	**4.79**	7.69	4.24	**6.99**	13.40
Handball	NA	**NA**	NA	NA	**NA**	NA	NA	**NA**	NA	NA	**NA**	NA	NA	**NA**	NA
Hockey Rink	NA	**NA**	NA	NA	**NA**	NA	NA	**NA**	NA	NA	**NA**	NA	NA	**NA**	NA
Korfball	NA	**NA**	NA	NA	**NA**	NA	NA	**NA**	NA	NA	**NA**	NA	NA	**NA**	NA
Modern Pentathlon	NA	**NA**	NA	NA	**NA**	NA	NA	**NA**	NA	NA	**NA**	NA	NA	**NA**	NA
Motorsport	NA	**NA**	NA	NA	**NA**	NA	NA	**NA**	NA	NA	**NA**	NA	NA	**NA**	NA
Other combat sports	NA	**NA**	NA	NA	**NA**	NA	NA	**NA**	NA	NA	**NA**	NA	NA	**NA**	NA
Rowing	NA	**NA**	NA	NA	**NA**	NA	NA	**NA**	NA	NA	**NA**	NA	NA	**NA**	NA
Rugby	NA	**NA**	NA	NA	**NA**	NA	NA	**NA**	NA	NA	**NA**	NA	NA	**NA**	NA
Sailing	NA	**NA**	NA	NA	**NA**	NA	NA	**NA**	NA	NA	**NA**	NA	NA	**NA**	NA
Soccer	NA	**NA**	NA	NA	**NA**	NA	NA	**NA**	NA	NA	**NA**	NA	NA	**NA**	NA
Surf	NA	**NA**	NA	NA	**NA**	NA	NA	**NA**	NA	NA	**NA**	NA	NA	**NA**	NA
Swimming	2.61	**3.48**	4.39	3.52	**4.34**	5.23	4.33	**5.06**	5.92	4.89	**5.90**	7.28	5.84	**7.36**	9.82
Tennis	NA	**NA**	NA	NA	**NA**	NA	NA	**NA**	NA	NA	**NA**	NA	NA	**NA**	NA
Triathlon	1.04	**2.20**	3.87	1.90	**3.27**	5.22	2.90	**4.31**	6.43	3.57	**5.69**	9.79	4.81	**8.47**	17.91
Volleyball	2.45	**3.84**	5.45	3.71	**5.14**	6.85	4.95	**6.30**	8.03	5.80	**7.73**	10.71	7.29	**10.35**	16.19
Wrestling and Judo	1.72	**2.76**	3.99	2.64	**3.72**	5.04	3.55	**4.59**	5.92	4.18	**5.65**	7.98	5.28	**7.63**	12.26
**Males**															
Archery and Shooting	NA	**NA**	NA	NA	**NA**	NA	NA	**NA**	NA	NA	**NA**	NA	NA	**NA**	NA
Athletics	1.42	**2.16**	2.91	1.95	**2.62**	3.36	2.43	**3.00**	3.70	2.68	**3.43**	4.61	3.09	**4.17**	6.32
Basketball	1.43	**2.07**	2.82	2.40	**3.13**	3.98	3.44	**4.17**	5.05	4.37	**5.55**	7.23	6.16	**8.38**	12.13
Fencing	NA	**NA**	NA	NA	**NA**	NA	NA	**NA**	NA	NA	**NA**	NA	NA	**NA**	NA
Gymnastics	NA	**NA**	NA	NA	**NA**	NA	NA	**NA**	NA	NA	**NA**	NA	NA	**NA**	NA
Handball	1.66	**2.59**	3.70	2.90	**3.98**	5.27	4.28	**5.37**	6.73	5.47	**7.24**	9.93	7.79	**11.14**	17.37
Hockey Rink	NA	**NA**	NA	NA	**NA**	NA	NA	**NA**	NA	NA	**NA**	NA	NA	**NA**	NA
Korfball	NA	**NA**	NA	NA	**NA**	NA	NA	**NA**	NA	NA	**NA**	NA	NA	**NA**	NA
Modern Pentathlon	NA	**NA**	NA	NA	**NA**	NA	NA	**NA**	NA	NA	**NA**	NA	NA	**NA**	NA
Motorsport	NA	**NA**	NA	NA	**NA**	NA	NA	**NA**	NA	NA	**NA**	NA	NA	**NA**	NA
Other combat sports	0.99	**1.91**	3.07	1.71	**2.71**	3.98	2.50	**3.46**	4.77	3.00	**4.41**	6.99	3.89	**6.26**	12.10
Rowing	NA	**NA**	NA	NA	**NA**	NA	NA	**NA**	NA	NA	**NA**	NA	NA	**NA**	NA
Rugby	1.07	**1.96**	3.20	2.40	**3.68**	5.38	4.21	**5.70**	7.72	6.04	**8.84**	13.56	10.16	**16.62**	30.51
Sailing	NA	**NA**	NA	NA	**NA**	NA	NA	**NA**	NA	NA	**NA**	NA	NA	**NA**	NA
Soccer	1.52	**2.16**	2.86	2.25	**2.90**	3.62	2.96	**3.55**	4.26	3.48	**4.35**	5.61	4.41	**5.84**	8.32
Surf	NA	**NA**	NA	NA	**NA**	NA	NA	**NA**	NA	NA	**NA**	NA	NA	**NA**	NA
Swimming	0.79	**1.31**	1.97	1.51	**2.16**	2.97	2.37	**3.05**	3.94	3.14	**4.32**	6.17	4.73	**7.11**	11.79
Tennis	0.88	**2.20**	4.18	1.83	**3.43**	5.77	3.02	**4.67**	7.23	3.78	**6.36**	11.97	5.23	**9.92**	24.69
Triathlon	1.88	**2.29**	2.70	2.41	**2.77**	3.15	2.85	**3.17**	3.52	3.18	**3.62**	4.17	3.72	**4.37**	5.33
Volleyball	1.68	**2.94**	4.46	2.82	**4.17**	5.83	4.03	**5.32**	7.02	4.85	**6.78**	10.05	6.34	**9.62**	16.83
Wrestling and Judo	1.14	**1.61**	2.15	1.84	**2.36**	2.95	2.57	**3.08**	3.68	3.21	**4.01**	5.15	4.41	**5.88**	8.32

NA: data not presented for n < 8.

Table 22 – Trunk fat mass (%) percentiles by sport and sex

Sport	0.05			0.25			Median			0.75			0.95		
	Low	Estimate	High	Low	Estimate	High	Low	Estimate	High	Low	Estimate	High	Low	Estimate	High
**Females**															
Archery and Shooting	NA	**NA**	NA	NA	**NA**	NA	NA	**NA**	NA	NA	**NA**	NA	NA	**NA**	NA
Athletics	4.56	**9.23**	12.70	9.28	**12.49**	15.21	12.57	**14.76**	16.95	14.31	**17.03**	20.23	16.82	**20.29**	24.96
Basketball	9.10	**13.07**	16.25	14.48	**17.23**	19.65	18.22	**20.12**	22.02	20.59	**23.01**	25.76	23.99	**27.17**	31.14
Fencing	NA	**NA**	NA	NA	**NA**	NA	NA	**NA**	NA	NA	**NA**	NA	NA	**NA**	NA
Gymnastics	0.12	**8.30**	14.16	7.72	**13.27**	17.87	13.01	**16.72**	20.44	15.58	**20.18**	25.72	19.28	**25.14**	33.32
Handball	NA	**NA**	NA	NA	**NA**	NA	NA	**NA**	NA	NA	**NA**	NA	NA	**NA**	NA
Hockey Rink	NA	**NA**	NA	NA	**NA**	NA	NA	**NA**	NA	NA	**NA**	NA	NA	**NA**	NA
Korfball	NA	**NA**	NA	NA	**NA**	NA	NA	**NA**	NA	NA	**NA**	NA	NA	**NA**	NA
Modern Pentathlon	NA	**NA**	NA	NA	**NA**	NA	NA	**NA**	NA	NA	**NA**	NA	NA	**NA**	NA
Motorsport	NA	**NA**	NA	NA	**NA**	NA	NA	**NA**	NA	NA	**NA**	NA	NA	**NA**	NA
Other combat sports	NA	**NA**	NA	NA	**NA**	NA	NA	**NA**	NA	NA	**NA**	NA	NA	**NA**	NA
Rowing	NA	**NA**	NA	NA	**NA**	NA	NA	**NA**	NA	NA	**NA**	NA	NA	**NA**	NA
Rugby	NA	**NA**	NA	NA	**NA**	NA	NA	**NA**	NA	NA	**NA**	NA	NA	**NA**	NA
Sailing	NA	**NA**	NA	NA	**NA**	NA	NA	**NA**	NA	NA	**NA**	NA	NA	**NA**	NA
Soccer	NA	**NA**	NA	NA	**NA**	NA	NA	**NA**	NA	NA	**NA**	NA	NA	**NA**	NA
Surf	NA	**NA**	NA	NA	**NA**	NA	NA	**NA**	NA	NA	**NA**	NA	NA	**NA**	NA
Swimming	9.54	**13.42**	16.41	13.88	**16.57**	18.90	16.89	**18.76**	20.63	18.63	**20.96**	23.65	21.12	**24.11**	27.98
Tennis	NA	**NA**	NA	NA	**NA**	NA	NA	**NA**	NA	NA	**NA**	NA	NA	**NA**	NA
Triathlon	0.00	**7.83**	15.01	6.59	**13.51**	19.17	12.86	**17.46**	22.06	15.75	**21.41**	28.33	19.91	**27.10**	37.35
Volleyball	8.62	**14.47**	18.85	14.50	**18.54**	21.97	18.58	**21.36**	24.15	20.75	**24.19**	28.23	23.88	**28.26**	34.11
Wrestling and Judo	2.54	**9.56**	14.71	9.55	**14.33**	18.34	14.43	**17.64**	20.86	16.95	**20.96**	25.73	20.57	**25.72**	32.74
**Males**															
Archery and Shooting	NA	**NA**	NA	NA	**NA**	NA	NA	**NA**	NA	NA	**NA**	NA	NA	**NA**	NA
Athletics	3.87	**7.09**	9.20	6.55	**8.66**	10.31	8.42	**9.75**	11.09	9.19	**10.84**	12.95	10.31	**12.41**	15.64
Basketball	1.46	**5.10**	8.00	6.95	**9.39**	11.53	10.77	**12.38**	13.99	13.23	**15.37**	17.81	16.76	**19.66**	23.30
Fencing	NA	**NA**	NA	NA	**NA**	NA	NA	**NA**	NA	NA	**NA**	NA	NA	**NA**	NA
Gymnastics	NA	**NA**	NA	NA	**NA**	NA	NA	**NA**	NA	NA	**NA**	NA	NA	**NA**	NA
Handball	0.13	**5.67**	9.96	7.67	**11.36**	14.54	12.91	**15.31**	17.72	16.09	**19.27**	22.96	20.67	**24.96**	30.50
Hockey Rink	NA	**NA**	NA	NA	**NA**	NA	NA	**NA**	NA	NA	**NA**	NA	NA	**NA**	NA
Korfball	NA	**NA**	NA	NA	**NA**	NA	NA	**NA**	NA	NA	**NA**	NA	NA	**NA**	NA
Modern Pentathlon	NA	**NA**	NA	NA	**NA**	NA	NA	**NA**	NA	NA	**NA**	NA	NA	**NA**	NA
Motorsport	NA	**NA**	NA	NA	**NA**	NA	NA	**NA**	NA	NA	**NA**	NA	NA	**NA**	NA
Other combat sports	0.00	**5.78**	9.95	5.17	**9.27**	12.46	9.20	**11.70**	14.20	10.94	**14.13**	18.23	13.44	**17.62**	24.03
Rowing	NA	**NA**	NA	NA	**NA**	NA	NA	**NA**	NA	NA	**NA**	NA	NA	**NA**	NA
Rugby	0.00	**3.95**	9.19	6.74	**11.21**	15.05	13.37	**16.26**	19.13	17.45	**21.30**	25.76	23.31	**28.56**	35.31
Sailing	NA	**NA**	NA	NA	**NA**	NA	NA	**NA**	NA	NA	**NA**	NA	NA	**NA**	NA
Soccer	3.62	**6.68**	9.01	7.31	**9.37**	11.13	9.87	**11.23**	12.60	11.34	**13.10**	15.16	13.46	**15.79**	18.85
Surf	NA	**NA**	NA	NA	**NA**	NA	NA	**NA**	NA	NA	**NA**	NA	NA	**NA**	NA
Swimming	0.00	**3.63**	6.60	5.02	**7.58**	9.78	8.66	**10.32**	11.98	10.86	**13.06**	15.62	14.04	**17.01**	20.86
Tennis	0.00	**6.29**	13.44	4.63	**11.87**	17.33	11.45	**15.75**	20.04	14.16	**19.63**	26.86	18.06	**25.21**	36.68
Triathlon	5.45	**7.33**	8.82	8.00	**9.29**	10.41	9.77	**10.65**	11.52	10.88	**12.01**	13.30	12.48	**13.96**	15.85
Volleyball	1.99	**7.68**	11.65	7.61	**11.35**	14.39	11.51	**13.91**	16.30	13.42	**16.46**	20.21	16.17	**20.14**	25.82
Wrestling and Judo	1.71	**4.52**	6.75	5.90	**7.79**	9.44	8.81	**10.06**	11.31	10.68	**12.33**	14.22	13.37	**15.60**	18.41

NA: data not presented for n < 8.

Table 23 – Trunk fat-free mass (kg) percentiles by sport and sex

Sport	0.05			0.25			Median			0.75			0.95		
	Low	Estimate	High	Low	Estimate	High	Low	Estimate	High	Low	Estimate	High	Low	Estimate	High
**Females**															
Archery and Shooting	NA	**NA**	NA	NA	**NA**	NA	NA	**NA**	NA	NA	**NA**	NA	NA	**NA**	NA
Athletics	17.13	**18.43**	19.75	19.71	**21.01**	22.33	21.51	**22.81**	24.12	23.30	**24.60**	25.92	25.87	**27.18**	28.50
Basketball	18.71	**19.61**	20.50	21.30	**22.18**	23.07	23.09	**23.98**	24.86	24.88	**25.77**	26.66	27.46	**28.35**	29.24
Fencing	NA	**NA**	NA	NA	**NA**	NA	NA	**NA**	NA	NA	**NA**	NA	NA	**NA**	NA
Gymnastics	13.20	**14.71**	16.23	15.79	**17.28**	18.81	17.58	**19.08**	20.60	19.38	**20.87**	22.39	21.95	**23.45**	24.98
Handball	NA	**NA**	NA	NA	**NA**	NA	NA	**NA**	NA	NA	**NA**	NA	NA	**NA**	NA
Hockey Rink	NA	**NA**	NA	NA	**NA**	NA	NA	**NA**	NA	NA	**NA**	NA	NA	**NA**	NA
Korfball	NA	**NA**	NA	NA	**NA**	NA	NA	**NA**	NA	NA	**NA**	NA	NA	**NA**	NA
Modern Pentathlon	NA	**NA**	NA	NA	**NA**	NA	NA	**NA**	NA	NA	**NA**	NA	NA	**NA**	NA
Motorsport	NA	**NA**	NA	NA	**NA**	NA	NA	**NA**	NA	NA	**NA**	NA	NA	**NA**	NA
Other combat sports	NA	**NA**	NA	NA	**NA**	NA	NA	**NA**	NA	NA	**NA**	NA	NA	**NA**	NA
Rowing	NA	**NA**	NA	NA	**NA**	NA	NA	**NA**	NA	NA	**NA**	NA	NA	**NA**	NA
Rugby	NA	**NA**	NA	NA	**NA**	NA	NA	**NA**	NA	NA	**NA**	NA	NA	**NA**	NA
Sailing	NA	**NA**	NA	NA	**NA**	NA	NA	**NA**	NA	NA	**NA**	NA	NA	**NA**	NA
Soccer	NA	**NA**	NA	NA	**NA**	NA	NA	**NA**	NA	NA	**NA**	NA	NA	**NA**	NA
Surf	NA	**NA**	NA	NA	**NA**	NA	NA	**NA**	NA	NA	**NA**	NA	NA	**NA**	NA
Swimming	16.54	**17.67**	18.78	19.12	**20.25**	21.36	20.92	**22.04**	23.15	22.71	**23.83**	24.95	25.28	**26.41**	27.53
Tennis	NA	**NA**	NA	NA	**NA**	NA	NA	**NA**	NA	NA	**NA**	NA	NA	**NA**	NA
Triathlon	15.13	**17.12**	18.44	17.72	**19.70**	21.01	19.51	**21.49**	22.80	21.30	**23.29**	24.60	23.88	**25.87**	27.18
Volleyball	17.76	**19.05**	20.38	20.34	**21.63**	22.96	22.14	**23.43**	24.75	23.93	**25.22**	26.54	26.51	**27.80**	29.13
Wrestling and Judo	16.19	**17.55**	18.89	18.77	**20.13**	21.46	20.57	**21.92**	23.26	22.36	**23.71**	25.05	24.93	**26.29**	27.63
**Males**															
Archery and Shooting	NA	**NA**	NA	NA	**NA**	NA	NA	**NA**	NA	NA	**NA**	NA	NA	**NA**	NA
Athletics	19.77	**24.20**	27.64	23.68	**26.91**	29.73	26.41	**28.79**	31.18	27.86	**30.68**	33.90	29.95	**33.39**	37.82
Basketball	22.75	**25.74**	28.24	27.24	**29.37**	31.31	30.36	**31.90**	33.44	32.49	**34.42**	36.56	35.55	**38.06**	41.05
Fencing	NA	**NA**	NA	NA	**NA**	NA	NA	**NA**	NA	NA	**NA**	NA	NA	**NA**	NA
Gymnastics	NA	**NA**	NA	NA	**NA**	NA	NA	**NA**	NA	NA	**NA**	NA	NA	**NA**	NA
Handball	22.83	**26.09**	28.79	27.25	**29.59**	31.70	30.33	**32.03**	33.72	32.35	**34.46**	36.80	35.26	**37.96**	41.23
Hockey Rink	NA	**NA**	NA	NA	**NA**	NA	NA	**NA**	NA	NA	**NA**	NA	NA	**NA**	NA
Korfball	NA	**NA**	NA	NA	**NA**	NA	NA	**NA**	NA	NA	**NA**	NA	NA	**NA**	NA
Modern Pentathlon	NA	**NA**	NA	NA	**NA**	NA	NA	**NA**	NA	NA	**NA**	NA	NA	**NA**	NA
Motorsport	NA	**NA**	NA	NA	**NA**	NA	NA	**NA**	NA	NA	**NA**	NA	NA	**NA**	NA
Other combat sports	17.19	**22.24**	26.17	21.98	**25.62**	28.79	25.31	**27.96**	30.61	27.13	**30.31**	33.94	29.75	**33.68**	38.73
Rowing	NA	**NA**	NA	NA	**NA**	NA	NA	**NA**	NA	NA	**NA**	NA	NA	**NA**	NA
Rugby	22.31	**26.06**	29.16	27.62	**30.28**	32.69	31.31	**33.22**	35.14	33.76	**36.16**	38.83	37.28	**40.39**	44.14
Sailing	NA	**NA**	NA	NA	**NA**	NA	NA	**NA**	NA	NA	**NA**	NA	NA	**NA**	NA
Soccer	21.32	**24.38**	26.88	25.08	**27.27**	29.24	27.69	**29.28**	30.89	29.33	**31.29**	33.50	31.69	**34.18**	37.25
Surf	NA	**NA**	NA	NA	**NA**	NA	NA	**NA**	NA	NA	**NA**	NA	NA	**NA**	NA
Swimming	20.34	**23.56**	26.21	24.72	**27.02**	29.08	27.76	**29.42**	31.08	29.76	**31.83**	34.12	32.63	**35.29**	38.50
Tennis	16.14	**21.65**	25.90	21.09	**25.07**	28.54	24.53	**27.45**	30.37	26.36	**29.83**	33.81	29.00	**33.26**	38.77
Triathlon	21.35	**23.43**	25.16	24.18	**25.68**	27.04	26.15	**27.24**	28.34	27.45	**28.81**	30.31	29.33	**31.06**	33.14
Volleyball	25.10	**29.47**	32.94	29.57	**32.73**	35.52	32.68	**35.00**	37.31	34.47	**37.26**	40.42	37.05	**40.52**	44.89
Wrestling and Judo	20.44	**23.36**	25.80	24.81	**26.88**	28.77	27.84	**29.34**	30.83	29.90	**31.79**	33.87	32.87	**35.32**	38.23

NA: data not presented for n < 8.

Table 24 – Trunk lean soft tissue (kg) percentiles by sport and sex

Sport	0.05			0.25			Median			0.75			0.95		
	Low	Estimate	High	Low	Estimate	High	Low	Estimate	High	Low	Estimate	High	Low	Estimate	High
**Females**															
Archery and Shooting	NA	**NA**	NA	NA	**NA**	NA	NA	**NA**	NA	NA	**NA**	NA	NA	**NA**	NA
Athletics	16.30	**17.59**	18.87	18.81	**20.08**	21.35	20.54	**21.82**	23.07	22.27	**23.55**	24.81	24.75	**26.04**	27.31
Basketball	18.00	**18.88**	19.75	20.50	**21.37**	22.24	22.24	**23.11**	23.96	23.96	**24.84**	25.70	26.44	**27.33**	28.20
Fencing	NA	**NA**	NA	NA	**NA**	NA	NA	**NA**	NA	NA	**NA**	NA	NA	**NA**	NA
Gymnastics	12.41	**13.88**	15.36	14.91	**16.37**	17.84	16.65	**18.10**	19.57	18.37	**19.83**	21.30	20.85	**22.32**	23.80
Handball	NA	**NA**	NA	NA	**NA**	NA	NA	**NA**	NA	NA	**NA**	NA	NA	**NA**	NA
Hockey Rink	NA	**NA**	NA	NA	**NA**	NA	NA	**NA**	NA	NA	**NA**	NA	NA	**NA**	NA
Korfball	NA	**NA**	NA	NA	**NA**	NA	NA	**NA**	NA	NA	**NA**	NA	NA	**NA**	NA
Modern Pentathlon	NA	**NA**	NA	NA	**NA**	NA	NA	**NA**	NA	NA	**NA**	NA	NA	**NA**	NA
Motorsport	NA	**NA**	NA	NA	**NA**	NA	NA	**NA**	NA	NA	**NA**	NA	NA	**NA**	NA
Other combat sports	NA	**NA**	NA	NA	**NA**	NA	NA	**NA**	NA	NA	**NA**	NA	NA	**NA**	NA
Rowing	NA	**NA**	NA	NA	**NA**	NA	NA	**NA**	NA	NA	**NA**	NA	NA	**NA**	NA
Rugby	NA	**NA**	NA	NA	**NA**	NA	NA	**NA**	NA	NA	**NA**	NA	NA	**NA**	NA
Sailing	NA	**NA**	NA	NA	**NA**	NA	NA	**NA**	NA	NA	**NA**	NA	NA	**NA**	NA
Soccer	NA	**NA**	NA	NA	**NA**	NA	NA	**NA**	NA	NA	**NA**	NA	NA	**NA**	NA
Surf	NA	**NA**	NA	NA	**NA**	NA	NA	**NA**	NA	NA	**NA**	NA	NA	**NA**	NA
Swimming	15.90	**16.99**	18.09	18.40	**19.49**	20.57	20.14	**21.22**	22.30	21.86	**22.95**	24.04	24.34	**25.44**	26.54
Tennis	NA	**NA**	NA	NA	**NA**	NA	NA	**NA**	NA	NA	**NA**	NA	NA	**NA**	NA
Triathlon	14.35	**16.01**	17.58	16.85	**18.50**	20.06	18.59	**20.23**	21.79	20.32	**21.96**	23.53	22.80	**24.45**	26.03
Volleyball	16.94	**18.22**	19.50	19.44	**20.71**	21.98	21.18	**22.44**	23.70	22.90	**24.17**	25.44	25.38	**26.66**	27.94
Wrestling and Judo	15.35	**16.69**	18.01	17.85	**19.18**	20.49	19.59	**20.91**	22.21	21.32	**22.64**	23.95	23.80	**25.13**	26.45
**Males**															
Archery and Shooting	NA	**NA**	NA	NA	**NA**	NA	NA	**NA**	NA	NA	**NA**	NA	NA	**NA**	NA
Athletics	19.59	**23.91**	27.27	23.41	**26.56**	29.32	26.07	**28.41**	30.74	27.49	**30.25**	33.40	29.54	**32.91**	37.23
Basketball	22.35	**25.20**	27.60	26.61	**28.65**	30.51	29.57	**31.05**	32.53	31.60	**33.45**	35.49	34.51	**36.90**	39.75
Fencing	NA	**NA**	NA	NA	**NA**	NA	NA	**NA**	NA	NA	**NA**	NA	NA	**NA**	NA
Gymnastics	NA	**NA**	NA	NA	**NA**	NA	NA	**NA**	NA	NA	**NA**	NA	NA	**NA**	NA
Handball	22.54	**25.61**	28.15	26.71	**28.91**	30.89	29.62	**31.21**	32.80	31.52	**33.50**	35.70	34.27	**36.81**	39.88
Hockey Rink	NA	**NA**	NA	NA	**NA**	NA	NA	**NA**	NA	NA	**NA**	NA	NA	**NA**	NA
Korfball	NA	**NA**	NA	NA	**NA**	NA	NA	**NA**	NA	NA	**NA**	NA	NA	**NA**	NA
Modern Pentathlon	NA	**NA**	NA	NA	**NA**	NA	NA	**NA**	NA	NA	**NA**	NA	NA	**NA**	NA
Motorsport	NA	**NA**	NA	NA	**NA**	NA	NA	**NA**	NA	NA	**NA**	NA	NA	**NA**	NA
Other combat sports	16.90	**21.88**	25.78	21.62	**25.22**	28.37	24.90	**27.54**	30.17	26.71	**29.86**	33.45	29.30	**33.19**	38.18
Rowing	NA	**NA**	NA	NA	**NA**	NA	NA	**NA**	NA	NA	**NA**	NA	NA	**NA**	NA
Rugby	22.00	**25.59**	28.57	27.06	**29.63**	31.94	30.58	**32.43**	34.28	32.92	**35.23**	37.79	36.29	**39.26**	42.85
Sailing	NA	**NA**	NA	NA	**NA**	NA	NA	**NA**	NA	NA	**NA**	NA	NA	**NA**	NA
Soccer	20.88	**23.85**	26.26	24.51	**26.64**	28.55	27.03	**28.59**	30.14	28.62	**30.53**	32.66	30.91	**33.33**	36.29
Surf	NA	**NA**	NA	NA	**NA**	NA	NA	**NA**	NA	NA	**NA**	NA	NA	**NA**	NA
Swimming	20.03	**23.14**	25.72	24.29	**26.51**	28.51	27.24	**28.85**	30.45	29.19	**31.19**	33.41	31.98	**34.55**	37.67
Tennis	15.84	**21.36**	25.63	20.81	**24.80**	28.28	24.27	**27.20**	30.12	26.11	**29.59**	33.58	28.76	**33.03**	38.55
Triathlon	20.81	**22.90**	24.64	23.68	**25.18**	26.54	25.67	**26.76**	27.86	26.99	**28.35**	29.85	28.89	**30.63**	32.71
Volleyball	25.55	**29.40**	32.45	29.44	**32.23**	34.70	32.14	**34.20**	36.27	33.71	**36.18**	38.97	35.96	**39.01**	42.86
Wrestling and Judo	19.91	**22.75**	25.13	24.16	**26.18**	28.02	27.11	**28.57**	30.03	29.12	**30.96**	32.98	32.01	**34.39**	37.23

NA: data not presented for n < 8.
